# T-bet Activates Th1 Genes through Mediator and the Super Elongation Complex

**DOI:** 10.1016/j.celrep.2016.05.054

**Published:** 2016-06-09

**Authors:** Arnulf Hertweck, Catherine M. Evans, Malihe Eskandarpour, Jonathan C.H. Lau, Kristine Oleinika, Ian Jackson, Audrey Kelly, John Ambrose, Peter Adamson, David J. Cousins, Paul Lavender, Virginia L. Calder, Graham M. Lord, Richard G. Jenner

**Affiliations:** 1 UCL Cancer Institute, University College London, 72 Huntley Street, W1T 4JF London, UK; 2 Department of Experimental Immunobiology and NIHR Comprehensive Biomedical Research Centre, Guy’s and St. Thomas’ Hospital and King’s College London, SE1 9RT London, UK; 3 UCL Institute of Ophthalmology, University College London, EC1V 9EL London, UK; 4 Department of Asthma, Allergy, and Respiratory Science, King’s College London, SE1 9RT London, UK; 5 Leicester Institute for Lung Health and Department of Infection, Immunity, and Inflammation, NIHR Leicester Respiratory Biomedical Research Unit, University of Leicester, LE3 9QP Leicester, UK

## Abstract

The transcription factor T-bet directs Th1 cell differentiation, but the molecular mechanisms that underlie this lineage-specific gene regulation are not completely understood. Here, we show that T-bet acts through enhancers to allow the recruitment of Mediator and P-TEFb in the form of the super elongation complex (SEC). Th1 genes are occupied by H3K4me3 and RNA polymerase II in Th2 cells, while T-bet-mediated recruitment of P-TEFb in Th1 cells activates transcriptional elongation. P-TEFb is recruited to both genes and enhancers, where it activates enhancer RNA transcription. P-TEFb inhibition and Mediator and SEC knockdown selectively block activation of T-bet target genes, and P-TEFb inhibition abrogates Th1-associated experimental autoimmune uveitis. T-bet activity is independent of changes in NF-κB RelA and Brd4 binding, with T-bet- and NF-κB-mediated pathways instead converging to allow P-TEFb recruitment. These data provide insight into the mechanism through which lineage-specifying factors promote differentiation of alternative T cell fates.

## Highlights

• Th1 genes and Th2 genes are associated with RNA pol II in the alternative lineage
• T-bet acts to recruit Mediator and the SEC to activate Th1 genes and eRNAs
• T-bet and NF-κB-dependent P-TEFb recruitment pathways converge at enhancers
• P-TEFb inhibition silences T-bet target genes and abrogates uveitis in vivo

## Graphical Abstract

**Figure:**
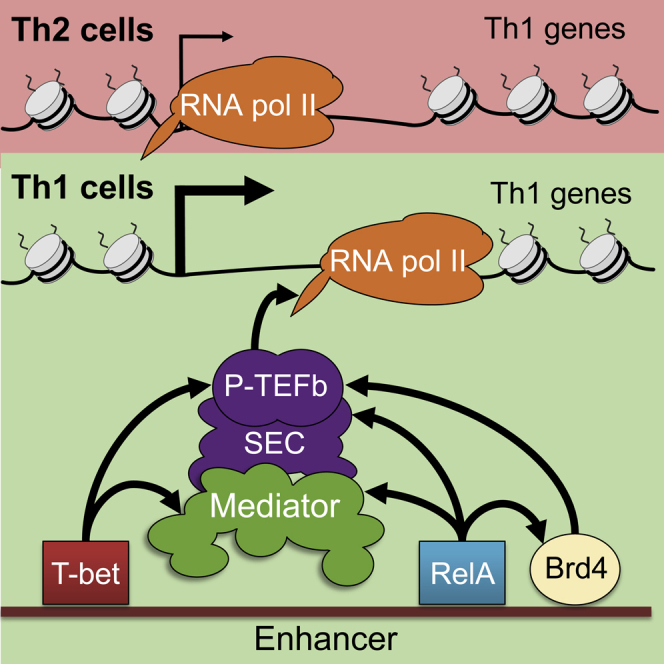

## Teaser

Lineage-specifying transcription factors control T helper cell fate choice, but the mechanisms underlying this are unclear. Hertweck et al. reveal that Th1 genes undergo transcriptional initiation in the alternative Th2 lineage and that the Th1 master regulator T-bet functions to recruit Mediator and the super-elongation complex to activate transcriptional elongation.

## Introduction

The differentiation of T helper cells into specialized effector lineages is a powerful model for understanding how master regulator transcription factors establish cell identity. Upon encounter with antigen, naive T cells can differentiate into one of several effector lineages (Zhu et al., 2010). The paradigm for the study of the T helper cell fate choice is the differentiation into either Th1 or Th2 lineages. Th1 cells activate cell-mediated immunity, essential to combat viral and intracellular bacterial infection, while Th2 cells orchestrate a humoral response to parasites. Inappropriate Th1 and Th2 responses are associated with autoimmunity and allergy, respectively. A degree of plasticity also exists between the different lineages and this may allow the immune response to be tuned as environmental cues vary (Murphy and Stockinger, 2010, O’Shea and Paul, 2010).

CD4 T cell fate choice is governed by a set of lineage-specifying transcription factors, which are activated differentially depending on the cytokine environment (Zhu et al., 2010). T-bet is necessary and sufficient for Th1 cell differentiation (Lazarevic et al., 2013, Szabo et al., 2000). How T-bet promotes Th1 differentiation has primarily been determined through the study of *Ifng.* T-bet activates *Ifng* by binding at the gene itself and to multiple enhancer elements spanning a 146 kb region up and downstream (Balasubramani et al., 2010, Hatton et al., 2006, Schoenborn et al., 2007, Shnyreva et al., 2004). T-bet has been reported to be necessary for recruitment of the NF-κB family member RelA (Balasubramani et al., 2010), the Setd7 H3K4 methyltransferase complex, and the H3K27 demethylase Kdm6b (Jmjd3; Miller et al., 2008) to *Ifng*, but the extent to which T-bet utilizes these mechanisms across the genome is unknown. T-bet also recruits p300 to the *Ifng* locus, but across the genome, only 17% of p300 binding sites in Th1 cells are dependent on T-bet, suggesting that it has a limited role in establishing the binding pattern of this co-factor (Vahedi et al., 2012).

Analysis of T-bet binding across the mouse and human genomes has revealed hundreds of immune regulatory genes at which T-bet binds across extended *cis*-regulatory regions similar to that at *Ifng* (Kanhere et al., 2012, Nakayamada et al., 2011, Zhu et al., 2012). Although T-bet binds to the promoters of thousands of genes, it only functions at the subset of genes associated with these extended regulatory regions (Kanhere et al., 2012).

Similar regions of dense transcription factor binding have been identified in a number of cell types and termed super-enhancers (Brown et al., 2014, Chapuy et al., 2013, Di Micco et al., 2014, Hnisz et al., 2013, Lovén et al., 2013, Whyte et al., 2013), transcription initiation platforms (Koch et al., 2011), or stretch enhancers (Parker et al., 2013). Super-enhancers are defined computationally as genomic regions that display unusually high levels of occupancy of a transcription factor or co-activator. Super-enhancers share similarities with locus control regions characterized by functional studies (Smith and Shilatifard, 2014), and genes associated with super-enhancers tend to be cell-type specific (Hnisz et al., 2013, Koch et al., 2011, Parker et al., 2013, Whyte et al., 2013). Thus, super-enhancer definition provides a useful tool for identifying a set of candidate regulatory loci important for the identity of the cell. As such, p300 binding has been used to identify super-enhancers and genes specific to Th1, Th2, and Th17 lineages (Vahedi et al., 2015).

In embryonic stem cells (ESCs), regions defined as super-enhancers are highly occupied by the co-activators Mediator, CBP, and p300, the BET protein Brd4, cohesin, and the Lsd1-NuRD complex (Hnisz et al., 2013, Whyte et al., 2013, Di Micco et al., 2014). The high levels of Brd4 binding at activated oncogenes in cancer cells (Chapuy et al., 2013, Lovén et al., 2013) and at proinflammatory genes in endothelial cells (Brown et al., 2014) and T cells (Peeters et al., 2015) renders them hyper-sensitive to transcriptional repression by BET-inhibitors, providing a potential therapeutic route for cancer and inflammatory diseases. However, the mechanisms by which T-bet functions at enhancers to activate Th1 gene expression are still unclear.

Using a combination of primary human T cells and mouse models, we report here that Th1 genes undergo transcriptional initiation in Th1 and Th2 cells and that T-bet activates Th1 genes through recruitment of Mediator and the super elongation complex, with Brd4 instead recruited in a parallel pathway dependent on NF-κB.

## Results

### Transcriptional Initiation at Th1 and Th2 Genes in the Opposing Cell Lineage

**Figure 1:**
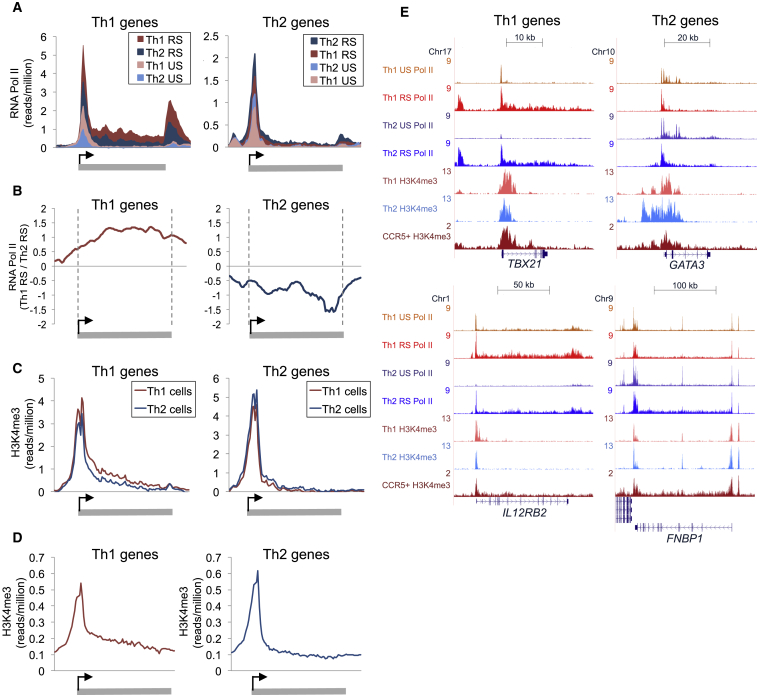
Transcriptional Initiation at Th1 and Th2 Genes in Both Lineages (A) Average number of ChIP-seq reads for RNA pol II (reads/million) across Th1 genes (left) and Th2 genes (right) in unstimulated (US) or restimulated (RS) human Th1 and Th2 cells. (B) Log2 ratio of RNA pol II across Th1 genes (left) and Th2 genes (right) between RS Th1 and Th2 cells. The difference in RNA pol II levels increases across the genes. (C) As in (A), except for H3K3me3 in Th1 and Th2 cells polarized for an extended period of time. (D) As in (A), except for H3K4me3 in CCR5+ Th1 memory cells. (E) ChIP-seq binding profiles for RNA pol II and H3K4me3 at example Th1 genes and Th2 genes in in vitro polarized Th1 and Th2 cells and in CCR5+ Th1 memory cells. See also Figure S1.

We hypothesized that profiling total RNA pol II occupancy by chromatin immunoprecipitation (ChIP) sequencing (ChIP-seq) in human Th1 and Th2 cells would provide insight into how T-bet functions to regulate gene expression. We first defined Th1 and Th2 genes as those expressed differentially across multiple samples, as done previously (see Wei et al., 2011, Hawkins et al., 2013, Stubbington et al., 2015). As expected, plotting ChIP-seq read density across Th1 genes in Th1 cells revealed occupancy of RNA pol II at transcription start sites (TSS), which increased upon restimulation (Figures 1A, 1E, and S1A–S1C; Table S1). RNA pol II was also present at Th1 genes in Th2 cells, albeit at a reduced level. Similarly, RNA pol II occupied Th2 genes in both Th1 and Th2 cells (Figures 1A, 1E, and S1A–S1C). Comparison of RNA pol II levels across Th1 genes between restimulated Th1 and Th2 cells revealed an increasing difference across the gene body, suggesting greater elongation efficiency in Th1 cells (Figure 1B). A corresponding effect was observed for Th2 genes, with RNA pol II showing increased elongation efficiency in Th2 cells (Figure 1B).

We sought to confirm transcriptional initiation of Th1 and Th2 genes in both lineages by measuring the initiation marker histone H3 trimethylated at lysine 4 (H3K4me3) in cells polarized for extended periods of time (28 days; Figures 1C, 1E, S1D, and S1E; Table S1). As we found for RNA pol II, H3K4me3 was present at Th1 and Th2 genes in both cell lineages. This was also apparent using an independent previously defined set of human Th1 and Th2 genes (Hawkins et al., 2013) (Figure S1G) and in murine Th1 and Th2 cells using our own and previously defined Th1 and Th2 gene sets (Wei et al., 2011, Stubbington et al., 2015) and our own and previously acquired H3K4me3 ChIP-seq data (Wei et al., 2009) (Figures S1H–S1J). To test whether this phenomenon also occurred in cells polarized in vivo, we performed ChIP-seq for H3K4me3 in human CCR5+ Th1 memory cells. This revealed the presence of H3K4me3 at both Th1 and Th2 genes in these cells, showing that this finding was not an artifact of in vitro polarization (Figures 1D, S1F, and S1G). Taken together with our RNA pol II binding data, these results show that transcription is initiated at Th1 and Th2 genes in both Th1 and Th2 cells and suggests that differential expression is primarily a function of differences in transcriptional elongation.

### Recruitment of P-TEFb to Th1 Genes and Super-Enhancers

We have previously shown that T-bet regulates gene expression by binding to multiple distal sites at extended *cis*-regulatory regions (Kanhere et al., 2012). We sought to determine whether these regions also fall into the definition of super-enhancers. Analysis of replicate T-bet ChIP-seq experiments using the ROSE algorithm (Hnisz et al., 2013) identified 374 super-enhancers in human Th1 cells (Figures S2A and S2B; Table S2). As expected, T-bet super-enhancers were associated with high levels of H3K27 acetylation (Figure S2C), CD4+ T cell-specific expression (Figure S2D), and functions related to the immune response (Figure S2E).

**Figure 2:**
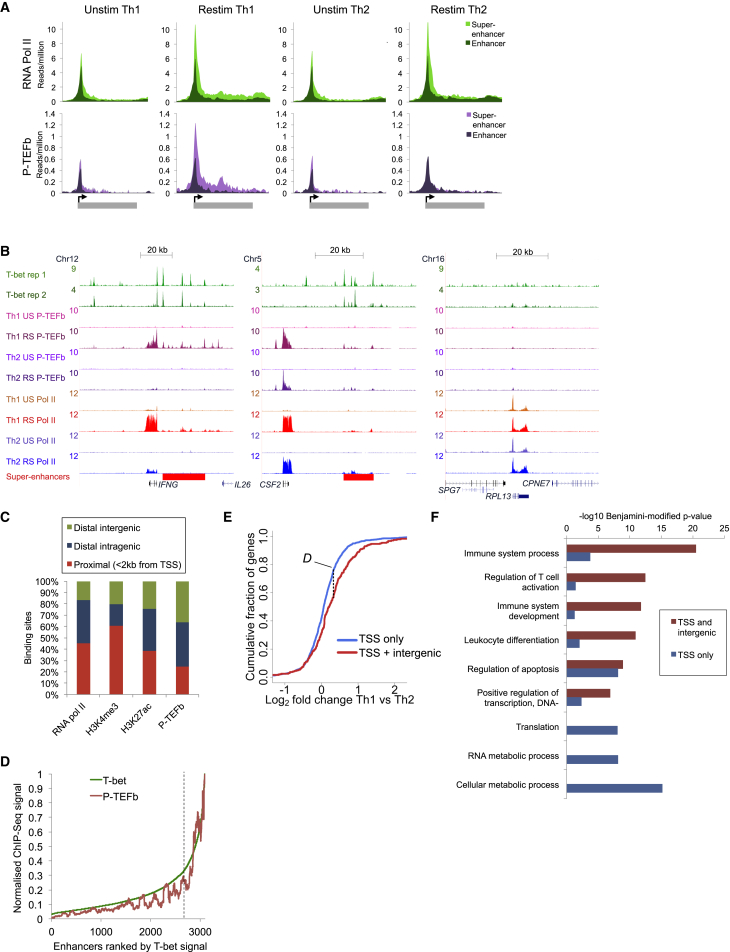
Extensive P-TEFb Binding at Super-Enhancers and Associated Genes (A) Average number of ChIP-seq reads for RNA pol II and P-TEFb in unstimulated (US) or restimulated (RS) human Th1 and Th2 cells. All genes are bound by RNA pol II in at least one condition and divided into those associated with a super-enhancer (n = 231) or a typical enhancer (n = 1,307). (B) T-bet, P-TEFb, and RNA pol II binding at *IFNG*, *CSF2* (associated with T-bet super-enhancers), and the housekeeping gene *RPL13*. (C) Percentage of sites for RNA pol II, H3K4me3, H3K27ac, and P-TEFb in Th1 cells that are proximal (<2 kb from TSS), distal intragenic, or distal intergenic. (D) Distribution of T-bet and P-TEFb ChIP-seq signals across 3,191 T-bet Th1 enhancers, ranked according to T-bet signal. The enhancers classified as T-bet super-enhancers are to the right of the vertical dashed line. The ChIP signals are shown as moving averages (window size of 100 bp). (E) Cumulative distribution frequency of gene expression in Th1 cells relative to Th2 cells for genes occupied by P-TEFb at the gene TSS only (blue, n = 970) or at the TSS and an intergenic site (red, n = 245) (*D* = 0.19, p = 7.47 × 10^−7^ [K-S test]). (F) Significance of the enrichment of biological process gene ontology categories in the set of genes occupied by P-TEFb at the gene TSS only (blue) or at the gene TSS and an intergenic site (red). See also Figure S2.

We next considered how T-bet function may be linked to lineage-specific transcriptional elongation and hypothesized that differential recruitment of the elongation factor P-TEFb may be involved. ChIP-seq for P-TEFb revealed increased recruitment in activated Th1 cells to genes with T-bet super-enhancers (Figures 2A, 2B, and S2F). In contrast, RNA pol II occupancy was similar at genes associated with super-enhancers and typical enhancers and was also comparable between Th1 and Th2 cells. Indeed, genes with super-enhancers exhibited significantly higher P-TEFb occupancy at their start sites compared to other active genes, even when considering the level of RNA pol II occupancy (p < 2 × 10^−16^ [K-S (Kolmogorov-Smirnov) test]; Figure S2G).

In addition to binding at genes, we unexpectedly found that P-TEFb also bound extensively to intergenic sites, with 35% of sites located outside of genes (Figures 2B and 2C), double that of RNA pol II (17%) and also greater than the active enhancer mark H3K27ac (24%). In intergenic regions, P-TEFb was localized to T-bet binding sites associated with H3K27ac (Figure S2H), with P-TEFb exhibiting particularly extensive binding at T-bet super-enhancers (Figures 2B and 2D). Indeed, 75% of genes associated with P-TEFb at the TSS and at an intergenic site were T-bet targets, compared with only 6% of genes where P-TEFb occupied the TSS only. The binding of P-TEFb to intergenic sites appeared to be functionally important; genes occupied by P-TEFb at the TSS and an intergenic site were overexpressed in Th1 cells relative to Th2 cells (Figure 2E). Furthermore, genes occupied by P-TEFb at the TSS and an intergenic site were enriched for functions related to the immune response, whereas, in comparison, genes only occupied by P-TEFb at the TSS had functions in cell metabolism and translation (Figure 2F). These data suggest that Th1 genes are regulated in a distinct manner involving P-TEFb recruitment to enhancers.

### Th1 Genes Are Hyper-Sensitive to Inhibition of Elongation

**Figure 3:**
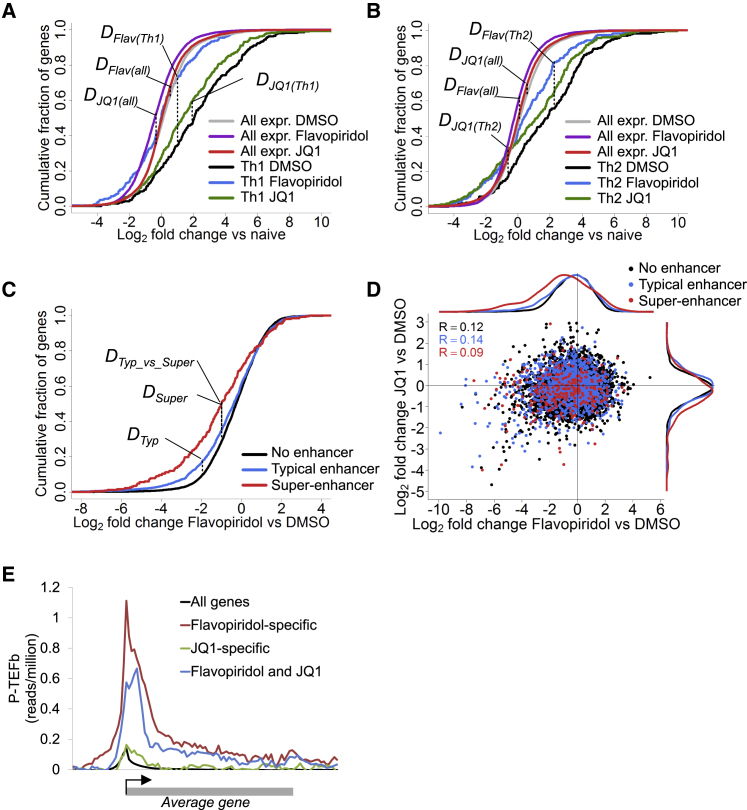
Th1 Genes Are Hyper-Sensitive to Inhibition of Transcriptional Elongation (A) Cumulative distribution frequency of gene expression changes in Th1 cells treated with DMSO, Flavopiridol (10 μM), or JQ1 (500 nM) relative to naive T cells. The genes are divided into Th1 genes (n = 291) or all expressed genes (n = 8,095). *D*_*Flav(all)*_ = 0.17, p < 2.2 × 10^−16^; *D*_*Flav(Th1)*_ = 0.39, p < 2.2 × 10^−16^; *D*_*JQ1(all)*_ = 0.053, p = 1.94 × 10^−10^; and *D*_*JQ1(Th1)*_ = 0.13, p = 0.011 (K-S test). (B) As in (A), except for Th2 genes (n = 228) or all expressed genes (n = 8,095) in Th2 cells. *D*_*Flav(all)*_ = 0.13, p < 2.2 × 10^−16^; *D*_*Flav(Th2)*_ = 0.26, p = 2.39 × 10^−7^; *D*_*JQ1(all)*_ = 0.054, p = 1.02 × 10^−10^; and *D*_*JQ1(Th2)*_ = 0.155, p = 0.92 × 10^−3^ (K-S test). (C) Expression changes of genes in Th1 cells in response to Flavopiridol versus DMSO control. Genes with typical T-bet enhancers (blue, n = 1,561), T-bet super-enhancers (red, n = 270), or neither (black, n = 6,264) are shown. *D*_*Typ*_ = 0.081, p = 1.21 × 10^−7^; *D*_*Super*_ = 0.22, p = 9.54 × 10^−12^; and *D*_*Typ_vs_Super*_ = 0.16, p = 1.55 × 10^−5^ (K-S test). (D) Scatterplot of changes in gene expression in Th1 cells in response to Flavopiridol versus changes in response to JQ1. The genes are divided as in (C). (E) Average ChIP-seq density for P-TEFb in human Th1 cells across all genes, genes repressed by both Flavopiridol and JQ1 (n = 232), and genes only repressed by either Flavopiridol (n = 109) or JQ1 (n = 128). See also Figure S3.

The high-level of P-TEFb binding at Th1 and Th2 genes argues that the switch from transcriptional initiation to elongation is a particularly critical control point in the expression of these genes. To test this, we measured gene expression in in vitro differentiated mouse Th1 and Th2 cells in the presence of the P-TEFb inhibitor Flavopiridol or the BET inhibitor JQ1, which inhibits P-TEFb recruitment (Brown et al., 2014, Chapuy et al., 2013, Di Micco et al., 2014, Lovén et al., 2013). We found that Th1 gene expression was significantly reduced by Flavopiridol and JQ1 (Figures 3A and S3A). The effect of Flavopiridol was much greater than that of JQ1, completely reversing the induction of Th1 genes. In contrast, other genes exhibiting the same expression levels were not substantially affected by either drug (Figures 3A and S3B). Similar results were observed for Th2 genes (Figure 3B). Thus, consistent with their high-levels of P-TEFb binding, Th1 and Th2 genes are hyper-sensitive to inhibition of transcriptional elongation compared to other expressed genes.

We next assessed whether the sensitivity of Th1 genes to elongation inhibition was related to super-enhancer function. We first identified the set of T-bet super-enhancers in mouse Th1 cells using replicate T-bet ChIP-seq data (Figure S3C; Table S2). Then, dividing genes into those associated with super-enhancers, typical enhancers, or neither, revealed that Flavopiridol blocked expression of genes associated with super-enhancers, but had little effect on those associated with typical enhancers (Figure 3C). Thus, super-enhancers show a specific requirement for P-TEFb to activate gene transcription.

Although JQ1 repressed a number of key Th1 genes (Figure S3E), the effect of JQ1 on Th1 gene expression was less striking than that of Flavopiridol (Figures 3D and S3E), suggesting that Brd4 does not play such a major role at these genes. Indeed, JQ1 and Flavopiridol had markedly different effects in Th1 cells, with a Pearson correlation co-efficient of only 0.12 (Figure 3D). This was also apparent with lower concentrations of each drug, indicating that this was not a reflection of differential off-target effects (Figure S3D). Flavopiridol was more specific for P-TEFb target genes than JQ1, as demonstrated by higher levels of P-TEFb binding at genes specifically repressed by Flavopiridol (Figures 3E and S3E; Table S3). These data suggest that Brd4 plays a relatively minor role in the recruitment of P-TEFb to T-bet target genes.

### T-bet Functions to Recruit P-TEFb to Genes and Enhancers in Activated Th1 Cells

**Figure 4:**
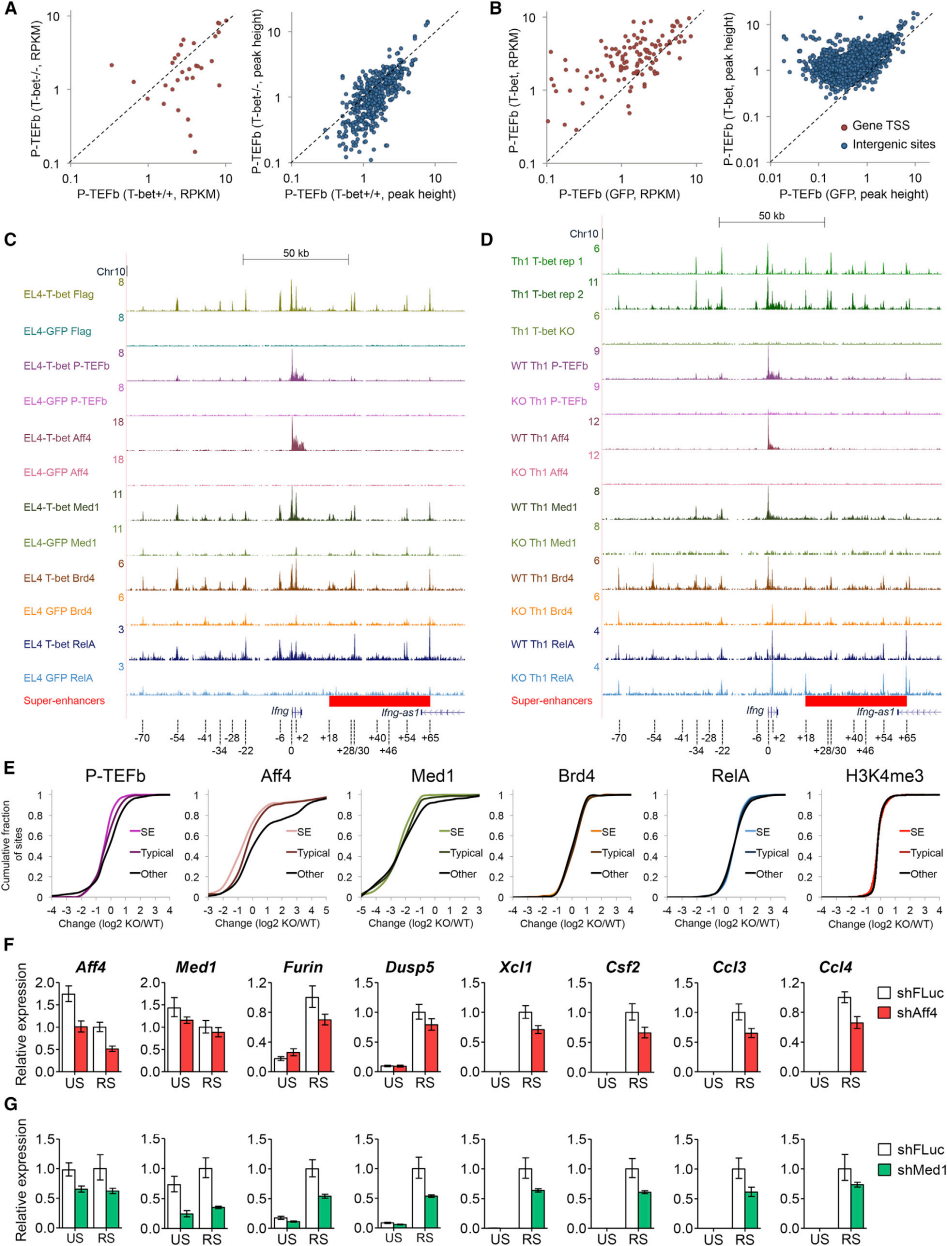
T-bet Is Necessary for Recruitment of Mediator, the SEC, and P-TEFb to Genes and Super-Enhancers (A) Scatterplot showing the read density (RPKM) for P-TEFb at super-enhancer-associated genes occupied by P-TEFb in primary mouse T-bet^+/+^ versus T-bet^−/−^ Th1 cells (left). A scatterplot showing the P-TEFb peak height (reads/million) at intergenic T-bet binding sites (p < 10^−9^) in primary mouse T-bet^+/+^ versus T-bet^−/−^ Th1 cells is on the right. (B) As in (A), except for activated EL4 cells stably expressing GFP (x axis) versus cells stably expressing T-bet and GFP (y axis). (C) ChIP-seq binding profiles for T-bet, P-TEFb, RelA, Brd4, Med1, and Aff4 in EL4 cells stably expressing GFP alone or EL4 cells stably expressing FLAG-T-bet and GFP, with both cell lines restimulated with PMA and ionomycin. The positions of transcription factor binding sites relative to the *Ifng* TSS are marked (to the nearest kb). (D) As in (C), except for WT and T-bet^−/−^ Th1 cells restimulated with PMA and ionomycin. (E) Cumulative distribution frequency of the change in P-TEFb, Aff4, Med1, Brd4, RelA, and H3K4me3 occupancy between T-bet^−/−^ and T-bet^+/+^ cells at super-enhancers and associated genes (SE), at typical enhancers and associated genes (Typical), and at other sites (Other). (F) Expression of *Aff4* and *Med1* and the T-bet target genes *Furin*, *Dusp5*, *Xcl1*, *Csf2*, *Ccl3*, and *Ccl4* relative to *Hprt* (mean and SD, n = 2 biological replicates) in unstimulated and restimulated Th1 cells transduced with retroviruses encoding shRNAs against luciferase (white) or Aff4 (red). (G) As in (F), except for shRNAs to luciferase (white) or Med1 (green) (mean and SD, n = 3 technical replicates). A replicate experiment with combined shRNA knock down of Med1 and Med17 is shown in Figure S4.

Given that P-TEFb bound with T-bet to super-enhancers and associated genes, we next asked whether T-bet functioned in the recruitment of P-TEFb. To test this, we first performed ChIP-seq for P-TEFb in restimulated CD4+ T cells purified from wild-type (WT) and T-bet^−/−^ mice cultured under Th1 conditions. We found that in the absence of T-bet, P-TEFb showed reduced binding to super-enhancers and to their associated genes (Figure 4A). Thus, T-bet is necessary for the high levels of P-TEFb recruitment observed at Th1 genes and enhancers.

To determine if T-bet was sufficient to recruit P-TEFb, we employed two murine EL4 cell lines, one that stably expresses T-bet and GFP and a control line expressing GFP alone (Kanhere et al., 2012). ChIP-seq for P-TEFb in both cell lines showed that P-TEFb occupancy increased at super-enhancers and their associated genes when T-bet was present (Figure 4B). We could also detect an interaction between T-bet and P-TEFb by co-immunoprecipitation (Figures S4A–S4C), consistent with a role for T-bet in P-TEFb recruitment. We conclude that T-bet functions to allow recruitment of P-TEFb to super-enhancers and associated genes in activated Th1 cells.

### T-bet Is Necessary for Recruitment of Mediator and the Super Elongation Complex

We next explored how T-bet functioned in the recruitment of P-TEFb to super-enhancers and their associated genes. P-TEFb can be recruited to genes through NF-κB (Barboric et al., 2001), the BET domain protein Brd4 (Jang et al., 2005, Yang et al., 2005), and by Mediator (Donner et al., 2010, Wang et al., 2013), which recruits P-TEFb as part of the super elongation complex (SEC) (Takahashi et al., 2011).

To test whether these mechanisms were in operation at T-bet super-enhancers, we measured the effect of T-bet expression in activated EL4 cells on the binding of RelA, Brd4, the Mediator subunit Med1, and the core SEC component Aff4. First focusing on *Ifng,* we found that T-bet induced binding of each protein to the promoter and to multiple distal regulatory elements that surround the gene (Figure 4C). To quantify the effect of T-bet on the binding of each factor across the genome, we measured the change in binding upon T-bet expression at all super-enhancers and associated genes compared to the change in binding at other sites across the genome and plotted the cumulative distribution frequencies (Figures S4D and S4E; Table S4). This revealed significant increases in P-TEFb, Aff4, Med1, and Brd4 occupancy at super-enhancers and their associated genes in EL4 cells when T-bet was present (p < 0.05, Mann-Whitney U test). Thus, we conclude that T-bet is sufficient for the recruitment of P-TEFb, Aff4, Med1, and Brd4 in activated cells.

We then asked whether T-bet was necessary for the recruitment of Aff4, Med1, Brd4, and RelA by measuring changes in their binding between restimulated WT and T-bet^−/−^ cells cultured under Th1 conditions. We also measured levels of the transcriptional initiation marker H3K4me3, to monitor this stage of gene activation. Comparing super-enhancers and their associated genes with other sites, we noted a marked deficiency in P-TEFb, Aff4, and Med1 recruitment in T-bet^−/−^ cells (p < 0.05, Mann-Whitney U test; Figures 4D, 4E, and S4F; Table S5). In contrast, recruitment of Brd4 and RelA were unaffected by T-bet deletion (p > 0.05; Figures 4E and S4F). Thus, T-bet is necessary for recruitment of Mediator and the SEC, but not for Brd4 and RelA binding. Consistent with a lack of a direct role for T-bet in RelA recruitment, a mutant form of T-bet (S508A), which does not interact with RelA (Hwang et al., 2005), had no effect on RelA binding at *Ifng* in EL4 cells (Figure S4G). H3K4me3 levels at super-enhancers and associated genes were also unaffected upon T-bet loss (Figure 4E), consistent with the presence of H3K4me3 at Th1 genes in Th2 cells (Figure 1) and confirming that T-bet does not act through super-enhancers to regulate this stage of gene activation.

Although T-bet deletion did not cause significant changes to Brd4 and RelA binding at super-enhancers compared to other sites, some binding events, such as the *Ifng* −54, −34 kb, and promoter sites, were dependent on T-bet (Figure 4D). To assess whether these T-bet-dependent sites were associated with P-TEFb recruitment, we identified the T-bet-dependent sites for each factor and measured the changes in P-TEFb binding at those locations (Figure S4H). We found that only T-bet-dependent recruitment of Med1 and Aff4 were associated with changes in P-TEFb occupancy. Thus, changes in Mediator and SEC binding, but not changes in Brd4 and RelA, are associated with T-bet-dependent P-TEFb recruitment.

To confirm whether Mediator and the SEC were important for the activation of T-bet target genes, we knocked down Med1 and Aff4 in mouse Th1 cells with small hairpin (sh)RNAs. We found that the expression of T-bet target genes such as *Ifng*, *Furin*, *Xcl1*, *Csf2, Ccl3*, and *Ccl4* were downregulated compared to the housekeeping gene *Hprt* (Figures 4F, 4G, S4I, and S4J). We conclude that T-bet operates through the Mediator-SEC pathway to allow recruitment of P-TEFb to super-enhancers and associated genes.

### RelA Is Necessary for Recruitment of P-TEFb, Mediator, Brd4, and the SEC to T-bet Target Genes

**Figure 5:**
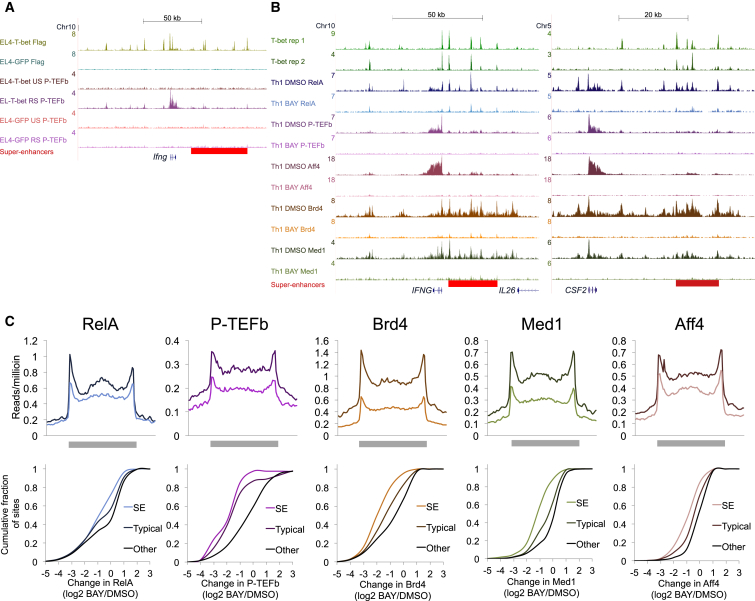
NF-κB Is Necessary for P-TEFb Recruitment to T-bet Target Genes and Super-Enhancers (A) ChIP-seq binding profiles for FLAG-T-bet and P-TEFb at the *Ifng* locus in EL4-GFP and EL4-T-bet cells with and without restimulation. (B) ChIP-seq binding profiles for RelA, P-TEFb, Brd4, Med1, and Aff4 at the *IFNG* locus in human Th1 cells with and without treatment with BAY 11-7082 (20 μM). (C) Average number of ChIP-seq reads for RelA, P-TEFb, Brd4, Med1, and Aff4 at T-bet super-enhancers in human Th1 cells with and without treatment with BAY 11-7082 (20 μM) (top). Cumulative distribution frequency of the change in transcriptional regulator binding (log_2_ BAY 11-7082 versus DMSO) for sites at super-enhancers and associated genes (SE), typical enhancers and associated genes, and at other sites bound by each factor (bottom). See also Figure S5.

The ability of T-bet to recruit P-TEFb also requires cell restimulation (Figures 2A, 2B, 5A, and S5A), suggesting that additional factors were also necessary. In other cell types, RelA can recruit P-TEFb to genes directly (Barboric et al., 2001), through Brd4 (Brown et al., 2014, Huang et al., 2009, Sharma et al., 2007), or through Mediator (van Essen et al., 2009, Wienerroither et al., 2015). In Th1 cells, RelA binds to, and is necessary for, activation of *Ifng* (Balasubramani et al., 2010, Sica et al., 1997). However, the role of NF-κB in P-TEFb recruitment and Th1 gene activation across the genome is unknown. To address this, we first performed ChIP-seq for RelA in human Th1 cells and found that it was associated with 75% of T-bet super-enhancers (Table S6). We then treated human Th1 cells with the IκB kinase inhibitor BAY 11-7082 and measured the change in RelA, P-TEFb, Brd4, Med1, and Aff4 recruitment by ChIP-seq. We found that IκB kinase inhibition significantly reduced the recruitment of all of these factors and that super-enhancers and their associated genes were particularly sensitive (p < 10^−7^, Mann-Whitney U test; Figures 5B, 5C, and S5B). Thus, in addition to T-bet, NF-κB has a central role in recruitment of the transcriptional elongation machinery to T-bet target genes and super-enhancers across the genome. Furthermore, the lack of change in RelA and Brd4 binding upon T-bet deletion (Figure 4E) indicates that these pathways operate independently and converge at super-enhancers to allow P-TEFb recruitment.

### T-bet and P-TEFb Function at Super-Enhancers to Activate Enhancer RNA Transcription

**Figure 6:**
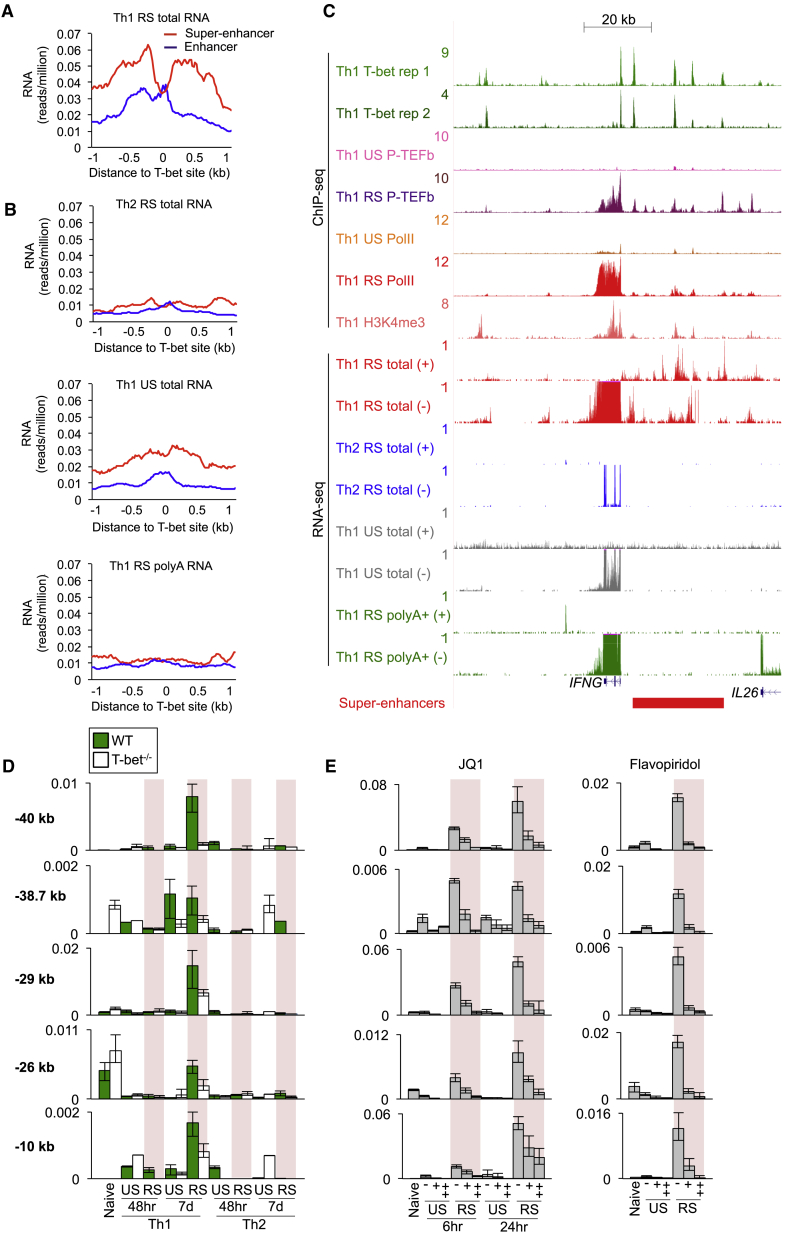
T-bet and P-TEFb-Dependent Production of Enhancer RNAs (A) Average total RNA-seq density (reads/million) in restimulated (RS) human Th1 cells at T-bet binding sites located within intergenic super-enhancers (n = 269) versus those within intergenic typical enhancers (n = 908). (B) As in (A), except for Th2 cells (same scale as A) (top). As in (A), except for unstimulated Th1 cells (middle). As in (A), except for mRNA in RS Th1 cells (bottom). (C) Total RNA and mRNA-seq data (reads/million) at *IFNG* showing production of non-poly-adenylated RNAs from the + (Watson) or − (Crick) strands in restimulated (RS) human Th1 cells. The read density at the *IFNG* gene extends beyond the maximum y axis value. The ChIP-seq binding profiles are shown above. (D) qRT-PCR for eRNAs (relative to *Hprt*, mean and SD, and n = 3 technical replicates) in WT and T-bet^−/−^ naive mouse T cells and unstimulated (US) or restimulated (RS) Th1 and Th2 cells polarized for 48 hr or 7 days. The eRNAs are labeled according to their position relative to the *Ifng* TSS. (E) As in (D), except for WT cells treated with 50 (+) or 500 nM (++) JQ1 (left) or treated with 1 (+) or 10 μM (++) Flavopiridol for 6 hr (right). See also Figure S6.

Given that P-TEFb bound extensively at super-enhancers and this was associated with increased requirement for P-TEFb function (Figure 3C), we sought to determine whether P-TEFb plays a role at super-enhancers themselves. In other cell types, some enhancers produce enhancer (e)RNAs that contribute to enhancer function (Lam et al., 2014, Natoli and Andrau, 2012). We therefore hypothesized that P-TEFb functioned at T-bet super-enhancers in the production of eRNAs. To test this, we performed total and poly-A+ RNA-seq in human Th1 and Th2 cells and, to control for the increased size of super-enhancers, compared the numbers of sequence reads around T-bet binding sites within intergenic super-enhancers versus typical enhancers. We found that eRNA transcription was higher at T-bet binding sites within super-enhancers compared to those at typical enhancers (Figures 6A and S6A). Furthermore, eRNAs transcribed from T-bet super-enhancers tended to be Th1-specific (Figures 6B and S6B). At *IFNG*, eRNAs were transcribed in Th1 cells from the super-enhancer upstream of the gene (Figures 6C and S6F); enhancers downstream of *IFNG* exhibited lower levels of P-TEFb occupancy and eRNA production. *IFNG* eRNAs displayed features previously ascribed to eRNAs, being transcribed bidirectionally, unspliced, and non-poly-adenylated (Figures 6C and S6F). eRNAs could also clearly be observed at super-enhancers associated with other key lineage-specific genes (Figure S6H; Table S7).

These data suggested that T-bet functions at super-enhancers to induce eRNA transcription. To test this, we measured expression of *Ifng* eRNAs in Th1 and Th2 cells from T-bet^−/−^ mice by quantitative (q)PCR. We found that the level of each eRNA tested was reduced in T-bet deficient cells, demonstrating that T-bet acts to induce eRNA transcription (Figure 6D). We next tested whether P-TEFb activity was required for *Ifng* eRNA production. Treatment of Th1 cells with JQ1 and Flavopiridol resulted in a marked reduction in eRNA levels, with Flavopiridol having the strongest effect (Figure 6E). Thus, P-TEFb functions at super-enhancers to activate eRNA transcription.

### Suppression of T-bet Function and Uveitis by P-TEFb Inhibition In Vivo

**Figure 7:**
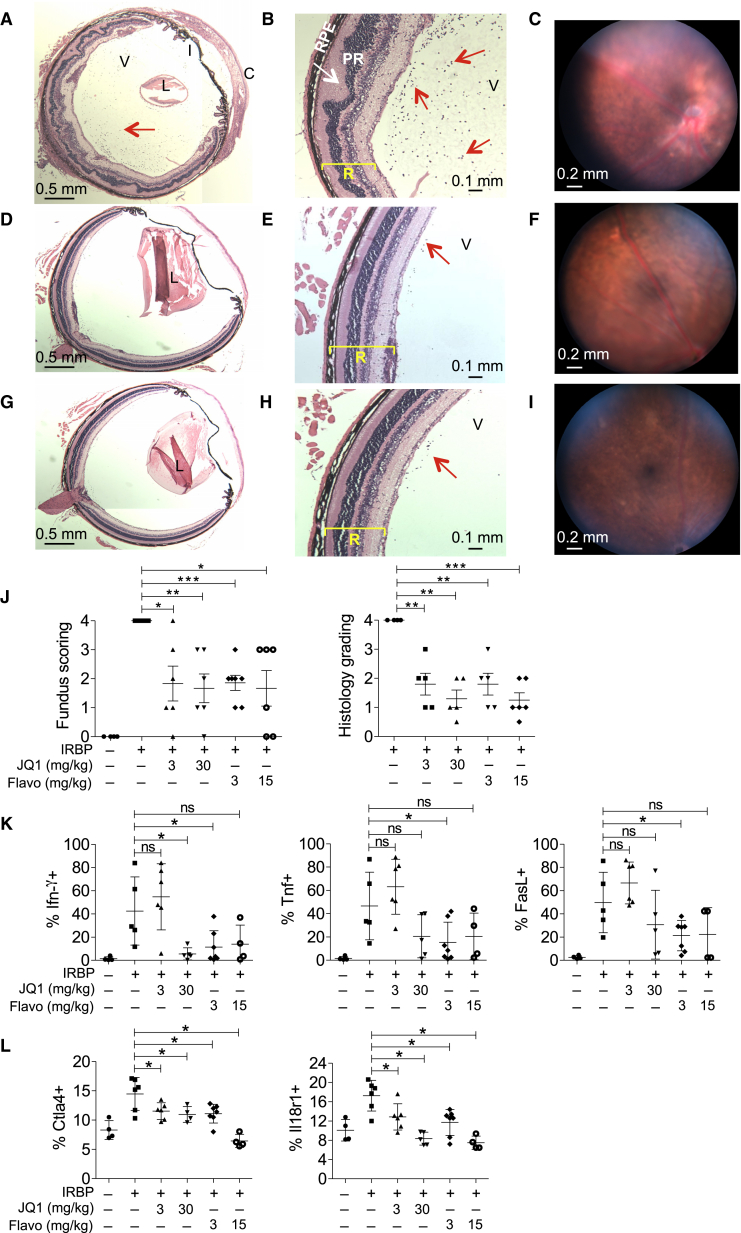
Alleviation of Uveitis by Inhibition of Transcriptional Elongation (A and B) Representative histopathology from an IRBP-immunized mouse treated with vehicle control (n = 6) showing severe EAU (grade 4) (choroid: C; iris: I; lens: L; vitreous: V, A; retinal layer: R; photoreceptors: PR; and retinal pigment epithelium: RPE, B) (yellow lines, retinal layers; red arrows, infiltrating cells; and white arrows, retina folding). Image in (A) is composed of two fields of view. (C) Representative in vivo fundoscopy of an IRBP-immunized mouse treated with vehicle control, exhibiting large confluent lesions with retinal atrophy. (D and E) As in (A) and (B), except for mice treated with JQ1 (30 mg/kg) for 5 days. (D) is composed of two fields of view. (F) As in (C), except for mice treated with JQ1 (30 mg/kg) for 5 days. Only very small, peripheral focal lesions were present. (G and H) As in (A) and (B), except for mice treated with Flavopiridol (15 mg/kg) for 5 days. (G) is composed of two fields of view. (I) As in (C), except for mice treated with Flavopiridol (15 mg/kg) for 5 days. (J) Mean (± SEM) fundoscopy scores for control mice and mice immunized with IRBP with and without treatment with JQ1 and Flavopiridol (left). The EAU retinal histology scores are shown (right) (^∗^p < 0.05, ^∗∗^p < 0.01, and ^∗∗∗^p < 0.005) (unpaired t test with Welch’s correction, two-tailed). (K) Percentage of IFNγ+, TNFα+, and FasL+ CD4+CD3+ T cells from the retina of non-immunized and IRBP-immunized mice treated with carrier, JQ1, or Flavopiridol (mean ± SD) (^∗^p < 0.05 and not significant: ns) (one-tailed Student’s t test). (L) As in (K), except for Il18r1+ and Ctla4+ CD4+CD3+ populations from the inguinal lymph nodes. See also Figure S7.

We sought to test the importance of P-TEFb in a Th1 response in vivo through use of a mouse experimental autoimmune uveitis (EAU) model, in which infiltration of interferon-γ-producing CD4+ T cells into the retina can be induced by immunization with interphotoreceptor retinoid-binding protein (IRBP) (Gardner et al., 2013). IRBP peptide was administered and, after 8 or 9 days, mice were treated with Flavopiridol (3 and 15 mg/kg) or JQ1 (3 and 30 mg/kg), and disease progression was scored by retinal fundoscopy and histology (Agarwal et al., 2012, Gardner et al., 2013). We found that both drugs significantly reduced disease severity. Immunized mice treated with carrier alone displayed severe EAU, characterized by disruption to retinal layers, diffuse retinal detachment, and folding, intense cellular infiltration and granulomatous lesions (histological score of 4; Figures 7A–7C and 7J). In contrast, mice treated with Flavopiridol and JQ1 showed reduced disease, with average histological scores between 1 and 2, minimal cell infiltration, and well-preserved photoreceptor layers (Figures 7D–7J, S7A, and S7B). We then sought to confirm that disease abrogation was reflected by a reduction in the expression of super-enhancer-associated Th1 genes. Flow cytometric analysis of CD4+ T cells sorted from the retina and lymph node revealed that expression of the super-enhancer-associated gene products Ifnγ, Tnf, Fasl, Il18r1, and Ctla4 were downregulated by Flavopiridol and JQ1 (Figures 7K, 7L, and S7C–S7G). We conclude that P-TEFb is required for Th1 gene expression in vivo and for Th1 cell-mediated immunopathology.

## Discussion

How T-bet regulates Th1 gene expression across the genome has been unclear. We show here that Th1 and Th2 genes undergo transcriptional initiation in a lineage-independent manner and that T-bet acts through extended regulatory regions (super-enhancers) to allow recruitment of Mediator and P-TEFb in the form of the SEC to activate Th1 gene expression. T-bet is necessary for P-TEFb recruitment, not just to gene promoters, but to super-enhancers themselves, where it functions to activate eRNA transcription. P-TEFb inhibition specifically downregulates Th1 genes and alleviates pathology in a Th1 cell-dependent uveitis model. T-bet is not required for RelA or Brd4 recruitment to most sites at Th1 genes. Instead, T-bet and NF-κB-dependent pathways converge at super-enhancers to allow P-TEFb recruitment. These data thus provide insight into the mechanisms of T-bet function during Th1 lineage-specification in human and mouse.

Mediator is a large multi-subunit complex that integrates signals from multiple transcription factors to modulate transcription, chromatin modification, and looping between promoters and enhancers (Carlsten et al., 2013). It is particularly important for transcription of genes related to cell type specification, but has not previously been shown to play a role in transcription of such genes in T cells. Mediator promotes transcriptional elongation by recruiting P-TEFb via Med23 (Wang et al., 2013) and the CDK8 submodule (Donner et al., 2010) or as part of the SEC via Med26 (Takahashi et al., 2011). The SEC allows rapid gene induction in ESCs (Lin et al., 2013), thus it may perform a similar role in CD4+ T cells.

Unlike other systems (Brown et al., 2014, Chapuy et al., 2013, Di Micco et al., 2014, Lovén et al., 2013, Peeters et al., 2015), T-bet does not operate by inducing large-scale changes in Brd4 binding. Instead, NF-κB activation is required for Brd4 recruitment in a parallel pathway. RelA has previously been shown to be required for *Ifng* expression downstream of the T cell receptor (TCR) (Balasubramani et al., 2010). We show here that RelA occupies the majority of T-bet bound super-enhancers and associated genes and, like T-bet, is necessary for recruitment of Mediator and the SEC, but is also necessary for Brd4 recruitment. However, there is no significant loss of RelA and Brd4 binding at T-bet super-enhancers and their associated genes compared to other sites in T-bet^−/−^ cells. Thus, T-bet and RelA operate through separate, but co-dependent pathways, that converge at super-enhancers to allow recruitment of P-TEFb to T-bet target genes. This model is consistent with the ability of enhancer clusters to integrate inputs from multiple signaling pathways (Spitz and Furlong, 2012) and may constitute a control mechanism to ensure that Th1 gene activation only occurs when multiple immunological signals are received.

How lineage-specifying factors function to promote differentiation toward a defined lineage, but also maintain the functional plasticity observed between effector subtypes has been a key unresolved issue. Epigenetic profiling has previously suggested that the establishment of bivalent chromatin at key lineage-specific transcription factors may be important (Wei et al., 2009). Our results reveal more generally that Th1 and Th2 genes remain associated with RNA pol II and H3K4me3 in the opposing lineage, and that T-bet acts to allow recruitment of Mediator and the SEC to activate transcriptional elongation. This mechanism may contribute to the functional plasticity observed between T helper cell subtypes.

We identified extensive association of P-TEFb with enhancers. Brd4 (Brown et al., 2014, Chapuy et al., 2013, Di Micco et al., 2014, Hnisz et al., 2013, Lovén et al., 2013, Zhang et al., 2012) and the elongation factor Ell3 (Lin et al., 2013) have previously been identified at enhancers, but the extent of P-TEFb binding at these sites has not previously been observed. Our results support the notion that P-TEFb functions at super-enhancers to activate eRNA transcription. Th1 cell eRNAs share similarities with those observed previously in other cell types, being predominantly non-poly-adenylated and transcribed bidirectionally (Lam et al., 2014, Natoli and Andrau, 2012). Previous reports disagree on whether eRNA production in other cell types requires transcriptional elongation, but the loss of eRNAs upon Flavopiridol and JQ1 treatment demonstrates that P-TEFb is required for eRNA transcription in Th1 cells.

Our study into the mechanisms through which T-bet directs Th1 lineage-specification suggests potential therapeutic avenues. BET inhibitors have previously been used to repress transcriptional elongation in immune cells and protect against inflammation in a number of models (Bandukwala et al., 2012, Brown et al., 2014, Mele et al., 2013, Nicodeme et al., 2010, Peeters et al., 2015). Although our experiments reveal a requirement for BET domain proteins for Th1 gene expression, Flavopiridol was the more selective for Th1 genes, consistent with the invariance in Brd4 binding upon T-bet deletion. Thus, direct inhibition of P-TEFb or Mediator may represent a more efficacious and specific means of targeting Th1 genes for the treatment of inflammatory and autoimmune conditions.

## Experimental Procedures

### Cells

Human and mouse naive T cells were isolated and cultured under Th1 and Th2 polarizing conditions for 13 days, as described (Kanhere et al., 2012). For H3K4me3 ChIP-seq, cells were differentiated for 28 days. Human CCR5+ Th1 memory T cells were purified as described (Messi et al., 2003). EL4-GFP and EL4-T-bet cells were described in Kanhere et al. (2012).

### Mice

C57BL/6 mice were purchased from Charles River Laboratories International. T-bet^−/−^ mice were purchased from Taconic. B10.RIII mice were obtained from GlaxoSmithKline. Mice were housed at the KCL Biological Service Unit (BSU) or at the UCL Institute of Ophthalmology BSU. Animal experiments were performed in accordance with the UK Animals (Scientific Procedures) Act 1986 (Home Office License Numbers PPL: 70/6792, 70/7869 and 70/7265).

### ChIP-Seq

ChIP was performed as described (Kanhere et al., 2012), except H3K4me3 ChIP was performed on native chromatin. All antibodies used and data sets generated are listed in the Supplemental Information. Libraries were constructed using standard Illumina protocols and were sequenced with an Illumina GAIIx or HiSeq 2500. Reads were filtered to remove adapters using fastq-mcf and for quality using seqkt and aligned to hg19 or mm9 with Bowtie2 (default settings). Consistency between replicates was assessed by irreproducible discovery rate (IDR) analysis; in each case, Np/Nt was less than 2, the standard reproducibility threshold used by the ENCODE project. Significantly enriched regions were identified with MACS v1.4 using a p value threshold of 10^−7^ unless indicated. Super-enhancers were identified with the ROSE algorithm (Lovén et al., 2013, Whyte et al., 2013, Hnisz et al., 2013). The significance of changes in transcription factor binding upon T-bet expression was assessed with a Mann-Whitney U test.

### Microarray Analysis

Total RNA was labeled using the two-color Low Input Quick Amp Labeling Kit and hybridized to SurePrint G3 DNA microarrays (Agilent). Differentially expressed genes were identified by rank-sum test (pfp < 0.05). Murine Th1 and Th2 genes and genes repressed by Flavopiridol and/or JQ1 were identified by applying fold-change expression thresholds. The significance of differences in expression between gene sets was assessed with a K-S test.

### Strand-Specific RNA-Seq

Poly-adenylated RNA was isolated with Oliogtex (QIAGEN). rRNA was depleted from total RNA with Ribo-Zero Gold (EpiCentre). Libraries were prepared using the Illumina Directional mRNA-Seq Sample Prep and the NEBNext Multiplex Small RNA Library Prep kits and then sequenced on an Illumina HiSeq 2500. RNA-seq reads were filtered for quality and to remove adapters, aligned to hg19 using TopHat2, and transcripts identified with Cufflinks v2.1.1 using default settings.

### EAU

B10.RIII mice were immunized subcutaneously with 300 μg IRBP_161–180_ (Cambridge Peptides) and monitored by fundoscopy on days 8–9 (Agarwal et al., 2012, Gardner et al., 2013). Flavopiridol (3 and 15 mg/kg) and JQ1 (3 and 30 mg/kg) were administered by daily intraperitoneal injection and disease progression scored by retinal fundoscopy and histology at days 14–15. Enucleated eyes were fixed, sectioned, stained with eosin, and counterstained with hematoxylin and graded (Agarwal et al., 2012). Single cell suspensions were prepared from retinas or inguinal lymph nodes for flow cytometry. The significance of changes in disease scoring and in the number of cells expressing T-bet target genes were assessed with a t test.

### shRNA Knockdown

Naive mouse CD4+ T cells were activated under Th1 polarizing conditions and transduced with pMY-Thy1.1-miR-30 retrovirus expressing shRNAs targeting firefly luciferase, Aff4, Med1, or Med17. After 3 days, Thy1.1+ cells were purified by magnetic cell sorting. On day 7, cells were either restimulated with 2 μg/ml anti-CD3/CD28 for 6 hr or left unstimulated.

## Author Contributions

R.G.J. and G.M.L. initiated the research program. A.H., C.M.E., M.E., P.A., P.L., D.J.C., V.L.C., G.M.L., and R.G.J. designed experiments. A.H., C.M.E., M.E., J.C.H.L., K.O., I.J., A.K., D.J.C., and R.G.J. performed experiments and interpreted data. R.G.J. led the bioinformatics work, with some analyses performed by A.H., C.M.E., and J.A. R.G.J. oversaw the research. A.H. and R.G.J. wrote the paper, with input from all authors.

## Accession Numbers

The accession number for the ChIP-seq, RNA-seq, and microarray data reported in this paper is GEO: GSE62486.

## Supplemental Information

Document S1. Supplemental Experimental Procedures and Figures S1–S7

Table S1. Th1 and Th2 Genes Identified by Gene Expression Profiling, Related to Figure 1 Worksheet 1 shows genes significantly more highly expressed in human Th1 or Th2 cells after 13 days polarization (Agilent human G3 arrays). Worksheet 2 shows genes significantly more highly expressed in human Th1 or Th2 cells after 28 days polarization (Affymetrix U133 plus 2 arrays).

Table S2. T-bet Super-Enhancers in Human and Mouse Th1 Cells, Related to Figure 2 The table shows coordinates of human and mouse T-bet super-enhancers and the closest RefSeq gene TSS. Some super-enhancers are associated with more than one gene (gene TSS lie within super-enhancers).

Table S3. Genes Exhibiting Differential Responses to JQ1 and Flavopiridol, Related to Figure 3 The table shows genes repressed in reactivated mouse Th1 cells upon by Flavopiridol, but not JQ1, JQ1 but not Flavopiridol, or by both compounds. Each gene set is provided in an individual tab of the table.

Table S4. P-TEFb, RelA, Med1, Aff4, Brd4, and H3K4me3 Binding Sites in EL4-T-bet and EL4-GFP Cells, Related to Figure 4 The number of sequencing reads at each binding site is given in the table, along with the position of the sites relative to murine T-bet super-enhancers and their associated genes (genes with closest TSS) and typical enhancers and their associated genes.

Table S5. P-TEFb, RelA, Med1, Aff4, and Brd4 Binding Sites in WT and T-bet^−/−^ Th1 Cells, Related to Figure 4 The number of sequencing reads at each binding site is given in the table, along with the position of the sites relative to murine T-bet super-enhancers and their associated genes (genes with closest TSS) and typical enhancers and their associated genes.

Table S6. P-TEFb, RelA, Med1, Aff4, and Brd4 Binding Sites in Restimulated Human Th1 Cells Treated with DMSO or BAY 11-7082, Related to Figure 5 The number of sequencing reads at each binding site is given in the table, along with the position of the sites relative to human T-bet super-enhancers and their associated genes (genes with closest TSS) and typical enhancers and their associated genes.

Table S7. T-bet Super-Enhancer eRNAs in Human Th1 and Th2 Cells, Related to Figure 6 The table shows coordinates of eRNAs identified from total RNA-seq in restimulated Th1 cells and their fragments per kilobase per million total reads (FPKM) values from total and poly-A+ RNA-seq in unstimulated (US) and restimulated (RS) Th1 and Th2 cells. The super-enhancer IDs correspond to the nearest RefSeq gene TSS. Some super-enhancers are associated with more than one gene and some genes are associated with more than one super-enhancer (delineated A and B). eRNA transcripts within each super-enhancer are numbered individually.

Document S2. Article plus Supplemental Information
