# Supplementary material for: T-bet Activates Th1 Genes through Mediator and the Super Elongation Complex
Source: Cell Rep. 2016 Jun 9;15(12):2756–70. doi: 10.1016/j.celrep.2016.05.054 (PMC4920892; doi:10.1016/j.celrep.2016.05.054)
Supplement: Document S1. Supplemental Experimental Procedures and Figures S1–S7 [file mmc1.pdf]

Cell Reports, Volume *15*

Supplemental Information

T-bet Activates Th1 Genes through Mediator and the Super Elongation Complex

Arnulf Hertweck, Catherine M. Evans, Malihe Eskandarpour, Jonathan C.H. Lau, Kristine Oleinika, Ian Jackson, Audrey Kelly, John Ambrose, Peter Adamson, David J. Cousins, Paul Lavender, Virginia L. Calder, Graham M. Lord, and Richard G. Jenner

Figure S1, related to Figure 1

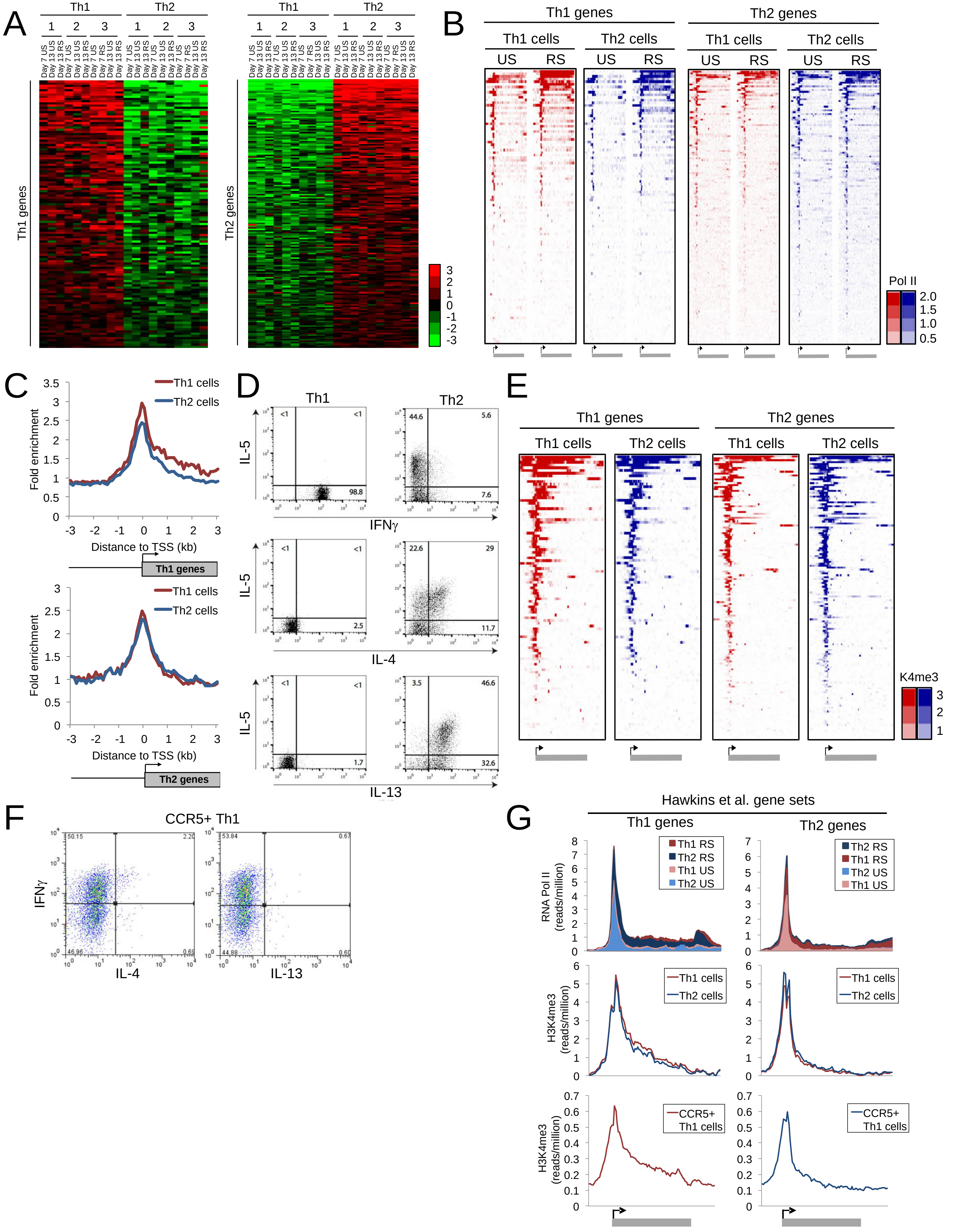

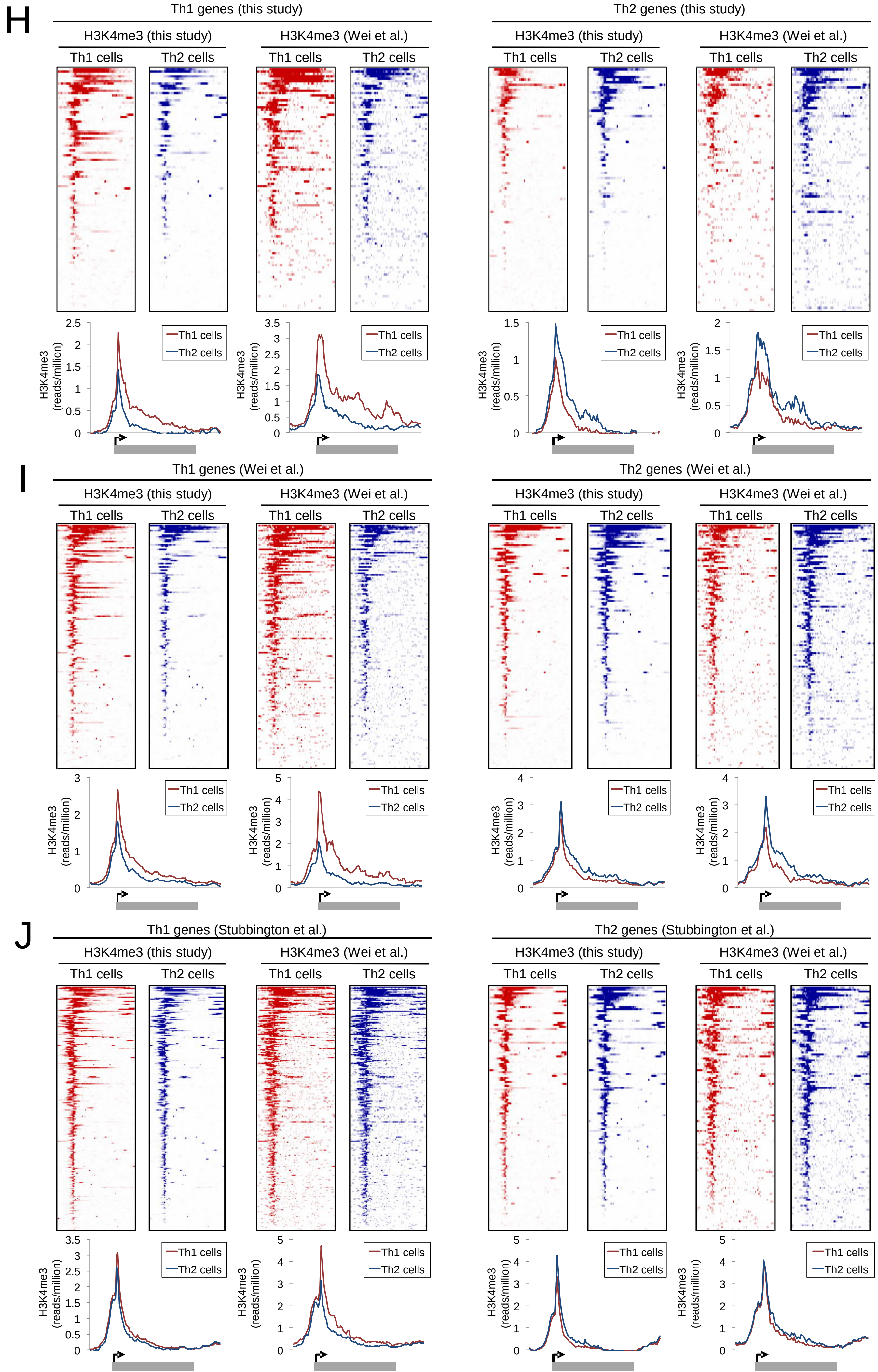
H
Th1 genes (this study)
Th2 genes (this study)
H3K4me3 (this study)
H3K4me3 (Wei et al.)
Th1 cells
Th2 cells
H3K4me3 (reads/million)
I
Th1 genes (Wei et al.)
Th2 genes (Wei et al.)
J
Th1 genes (Stubbington et al.)
Th2 genes (Stubbington et al.)

Figure S2, related to Figure 2

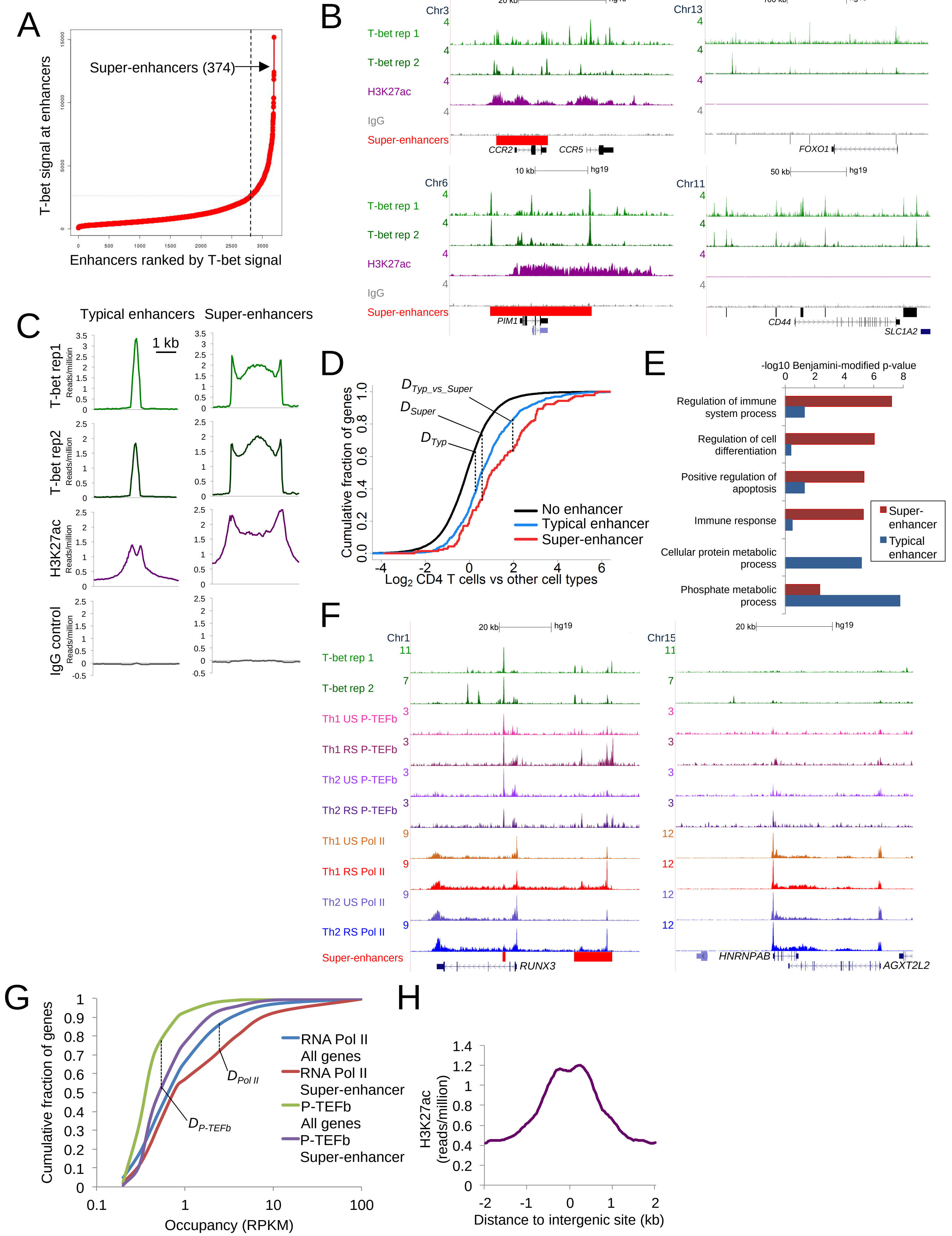

Figure S3, related to Figure 3

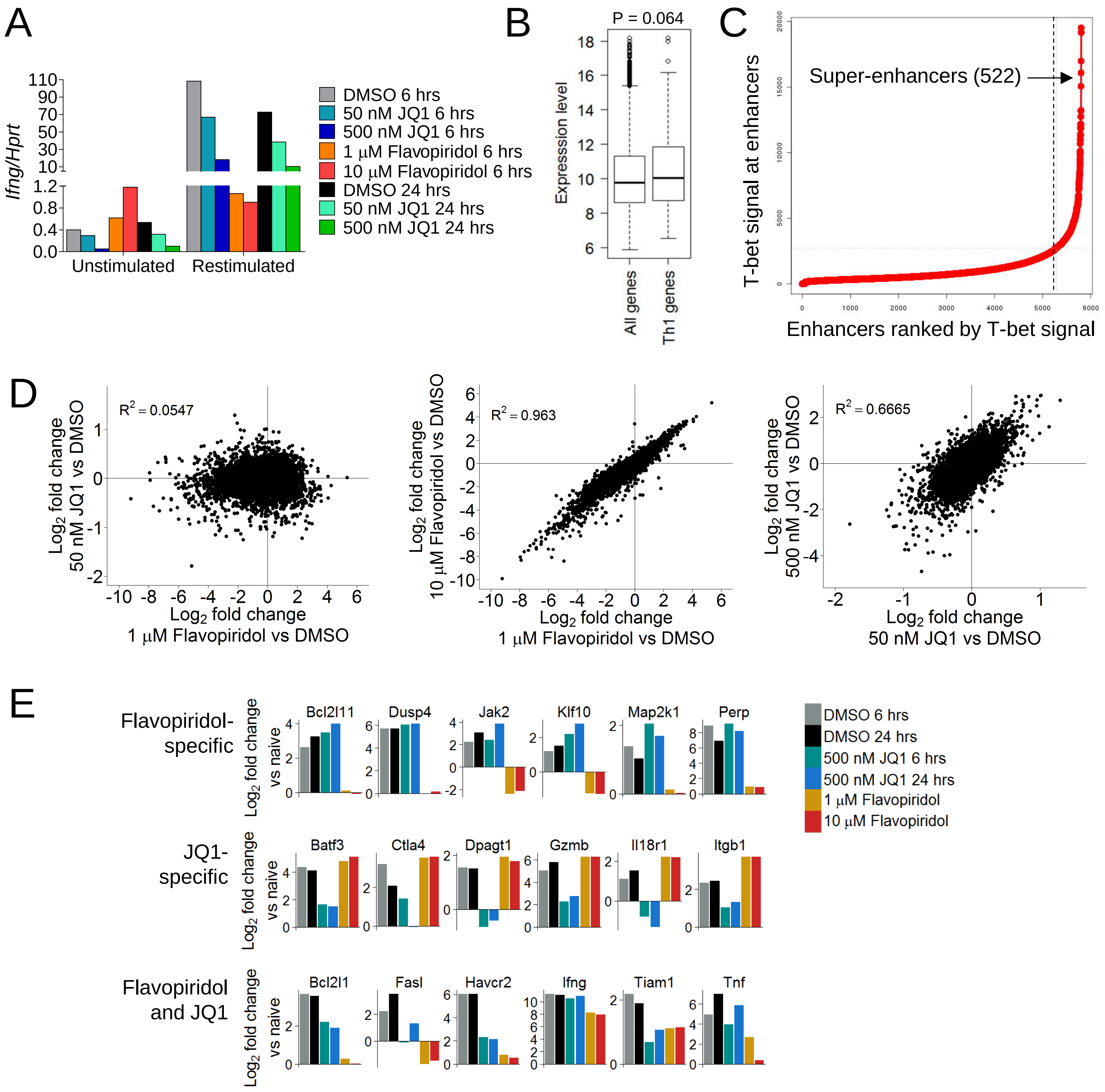

Figure S4, related to Figure 4

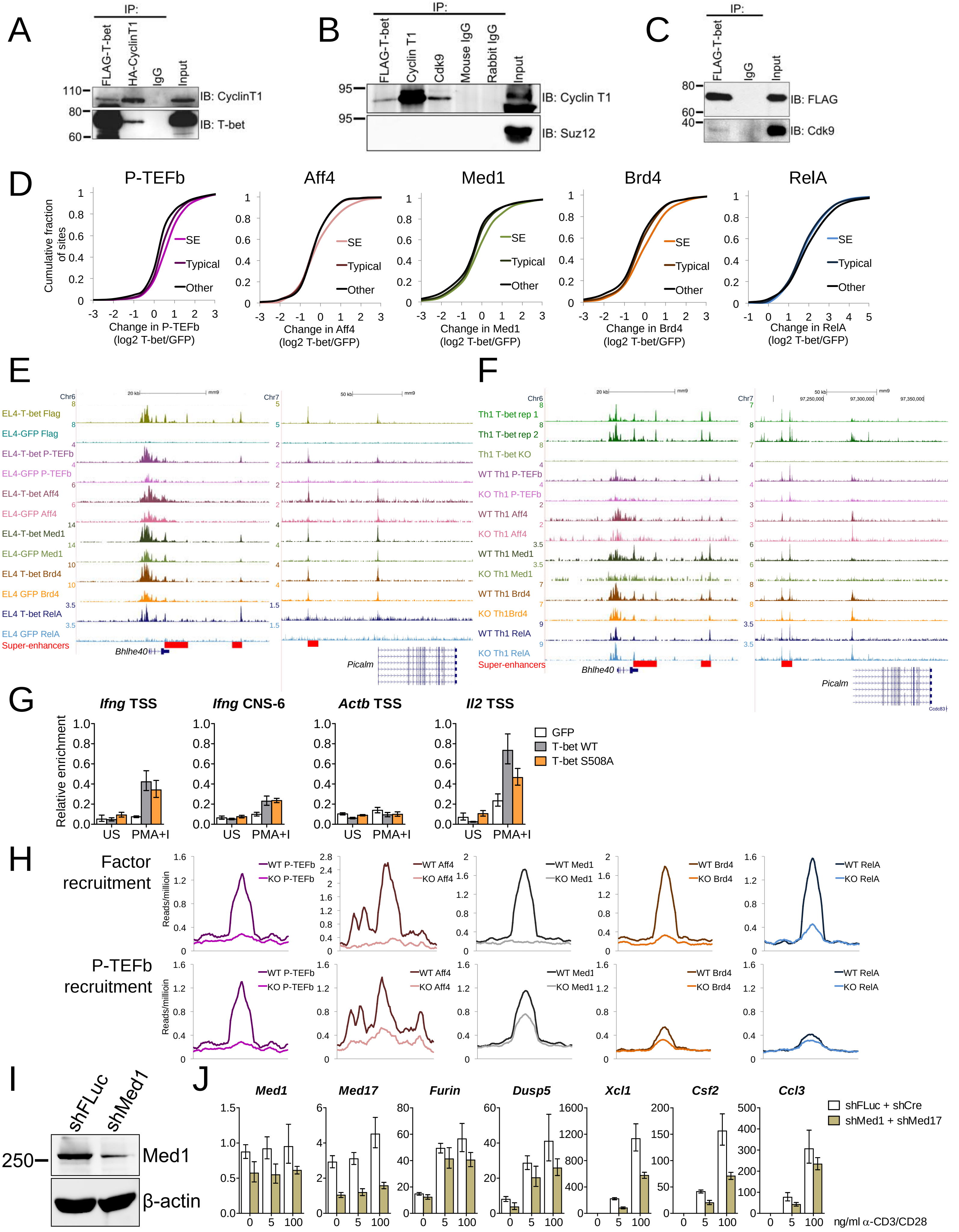

Figure S5, related to Figure 5

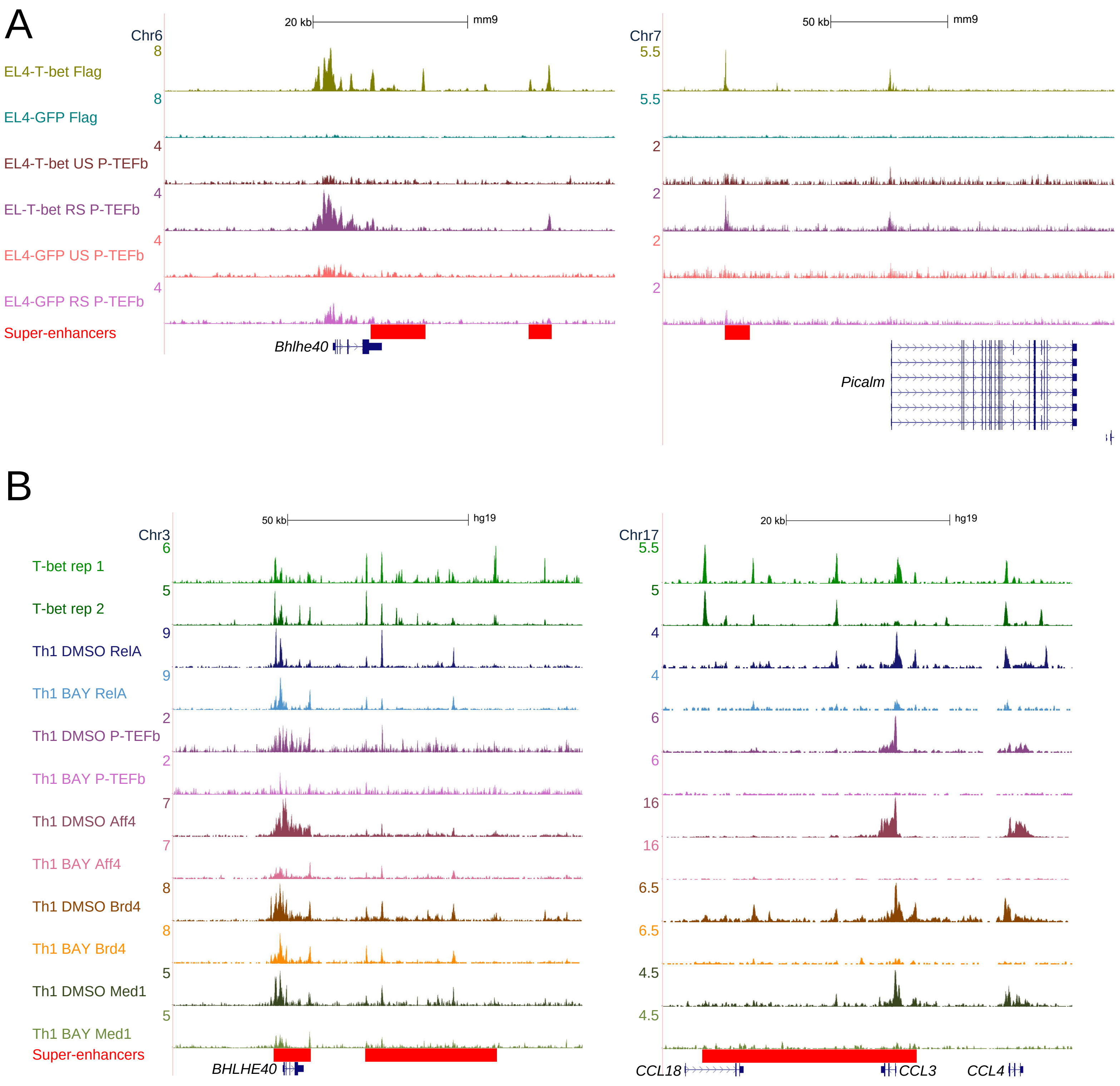

Figure S6, related to Figure 6

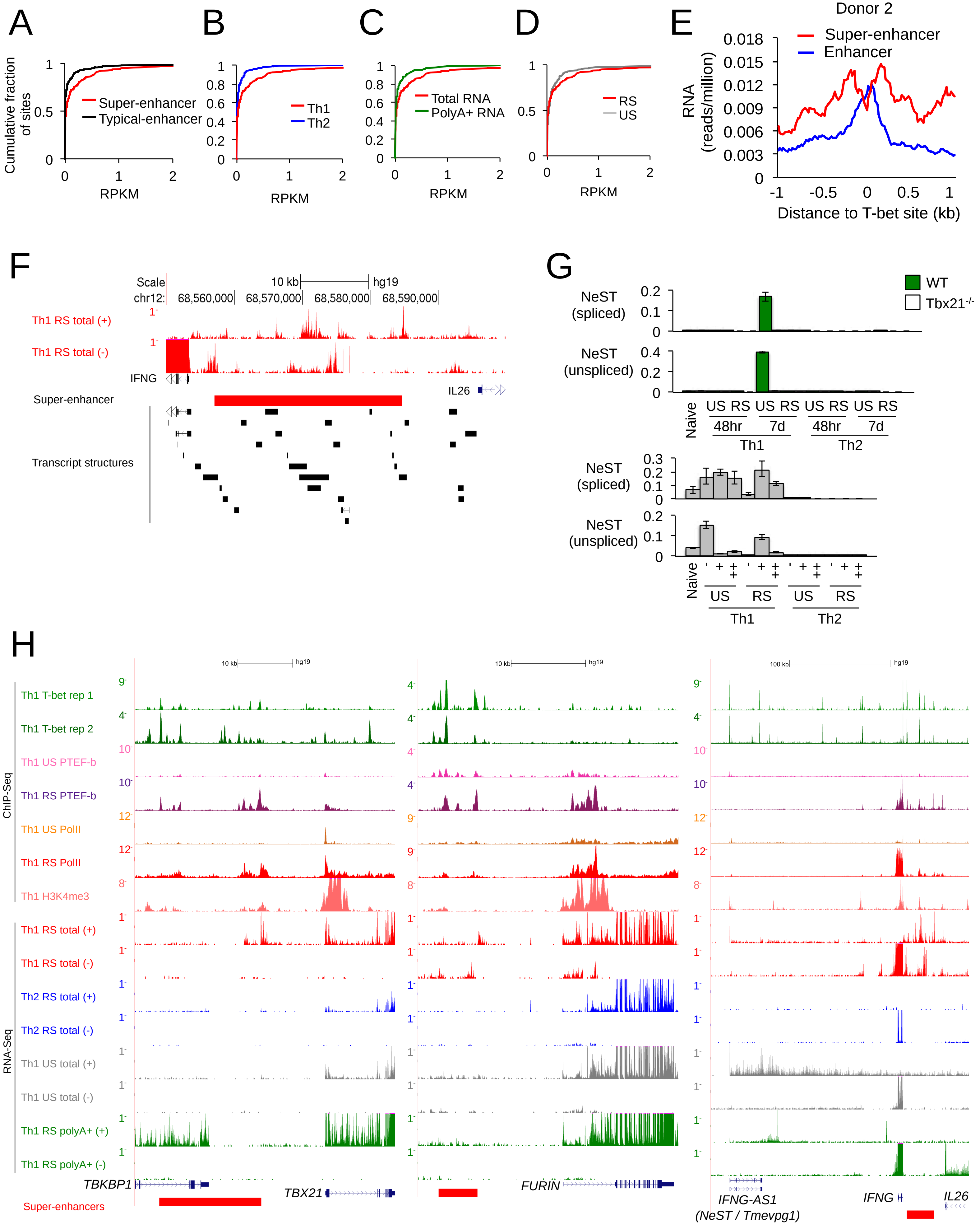

Figure S7, related to Figure 7

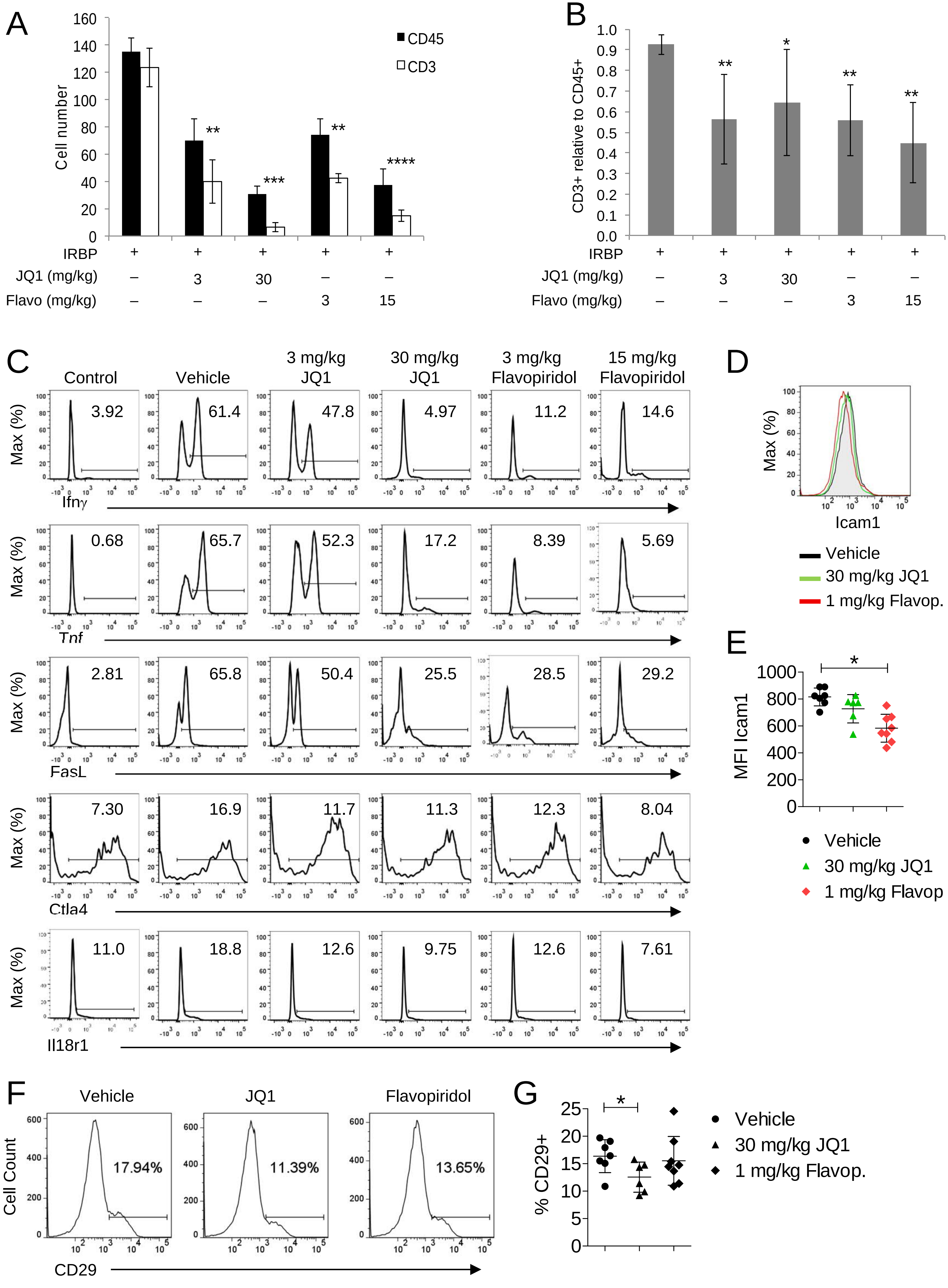

SUPPLEMENTAL FIGURE LEGENDS

Figure S1, related to Figure 1. RNA pol II and H3K4me3 in *in vitro* differentiated human and mouse Th1 and Th2 cells and *in vivo* polarized human CCR5+ cells.

A. Gene expression relative to naïve in Th1 and Th2 cells polarised *in vitro* from naïve cells purified from 3 donors and harvested at days 7 or 13 before (US) or after restimulation (RS) with anti-CD3/CD28 antibodies (n=10 Th1 samples, 10 Th2 samples). Genes are divided into those significantly more highly expressed in Th1 cells versus Th2 cells ("Th1 genes"; left panel, pfp<0.05, Rank sum test) or significantly more highly expressed in Th2 cells versus Th1 cells "Th2 genes"; right panel). All genes are also upregulated versus naïve cells by > 2-fold. Gene expression is represented by a color, with the scale (log2 ratio to naïve) shown at the far right.

B. Heat maps showing RNA pol II at each individual Th1 genes (left) and Th2 genes (right) that are averaged in Figure 1A. Each row represents a gene. The arrow under graph indicates transcriptional start site and the grey bar the gene body. RNA pol II occupancy is indicated by color intensity, according to the scale on the right.

C. Average enrichment profile of the initiation form of RNA pol II (detected by the 8WG16 antibody) at Th1 genes (top) and Th2 genes (bottom) in Th1 cells (red) and Th2 cells (blue). The plot shows average fold-enrichment (normalized signal from ChIP-enriched DNA divided by the signal from input DNA). The start and direction of transcription of the average gene is indicated by an arrow.

D. Staining for IFN-γ, IL4, IL5 and IL13 in human *in vitro* polarised Th1 and Th2 cells used for H3K4me3 ChIP-seq.

E. As B., except for H3K3me3 in unstimulated Th1 and Th2 cells.

F. Staining for IFN-γ, IL4 and IL13 in human CCR5+ memory Th1 cells purified from PBMC and used for H3K4me3 ChIP-seq.

G. Top: Average enrichment profile for RNA pol II in unstimulated (US) and restimulated (RS) Th1 and Th2 cells at Th1 and Th2 gene sets previously defined by Hawkins and colleagues (Hawkins et al., 2013). Middle: Average enrichment profile for H3K4me3 in Th1 and Th2 cells at Hawkins *et al.* Th1 and Th2 gene sets. Bottom: Average enrichment profile for H3K4me3 in CCR5+ Th1 cells at Hawkins *et al.* Th1 and Th2 gene sets.

H. Heat maps (top) and average enrichment profiles (bottom) for H3K4me3 across gene bodies +/- 2 kb in murine Th1 and Th2 cells at Th1 and Th2 gene sets identified in this study. Murine Th1 and Th2 H3K4me3 ChIP-seq data is from this study or from the study of Wei and colleagues (Wei et al., 2009).

I. As H., except at Th1 and Th2 gene sets derived from the RNA-seq data of Wei and colleagues (We et al., 2011).

J. As H., except at Th1 and Th2 gene sets previously defined by Stubbington and colleagues (Stubbington et al., 2015).

Figure S2, related to Figure 2. Identification of T-bet super-enhancers in human T cells and their association with P-TEFb.

A. Distribution of T-bet ChIP-seq signal (input-subtracted total reads) across 3,191 T-bet Th1 enhancers in humans from duplicate ChIP-seq experiments, generated by ROSE (Hnisz et al., 2013). T-bet occupancy is not evenly distributed across the enhancer regions, with a subset of enhancers to the right of the point of inflection (the 374 super-enhancers) containing especially high amounts of T-bet.

B. ChIP-seq binding profiles (reads/million, input subtracted) for T-bet and IgG control and for H3K27ac (total H3 subtracted) at example loci associated either with a super-enhancer (left, red bars) or typical enhancer (right, black bars). Scale bars are shown above each panel.

C. Average ChIP-seq density (reads/million, input subtracted) for T-bet, H3K27ac and IgG control at typical T-bet enhancers and super-enhancers in human Th1 cells.

D. Cumulative frequency distribution of log2 gene expression ratios (CD4 T cell versus average of all other cell types) for genes bound by T-bet at the TSS and associated with a typical T-bet enhancer (n=1041), T-bet super-enhancer (n=219) or neither enhancer type (n=12,221). Distances (*D*) are marked with black dashed lines. Typical enhancer vs all genes D_{Typ}=0.28, p<$2.2x10^{-16}$, Super-enhancers vs all genes D_{Super}=0.43, p<$2.2x10^{-16}$, Typical enhancer vs super enhancer $D_{Typ\_vs\_Super}$=0.19, p=$3.2x10^{-4}$ (Kolmogorov-Smirnov (K-S) test).

E. Significance (-log10 Benjamini-modified p-value) of the enrichment of biological process gene ontology categories in the set of genes associated with T-bet super-enhancers versus typical enhancers.

F. ChIP-seq binding profiles (reads/million, input subtracted) for T-bet, P-TEFb and RNA pol II in unstimulated (US) and restimulated (RS) Th1 and Th2 cells at the super-enhancer-associated gene *RUNX3* and the housekeeping gene *HNRNPAB.*

G. Cumulative frequency distributions of RNA pol II and P-TEFb occupancy (reads per kb per million total reads) in restimulated Th1 cells at genes associated with super-enhancers (n=322), compared with all genes (n=13,338). D_{PolII}=0.15, p=$2.4\text{x}10^{-6}$; $D_{P\text{-}TEFb}$=0.31, p<$2.2\text{x}10^{-16}$ (K-S test).

H. Average number of ChIP-seq reads for H3K27ac (reads/million, total H3 subtracted) in restimulated Th1 cells, centred on intergenic P-TEFb binding sites.

Figure S3, related to Figure 3. Gene expression profiling of murine Th1 and Th2 cells treated with JQ1 and Flavopiridol.

A. Quantitative RT-PCR for Ifng in Th1 cells treated with JQ1, Flavopiridol and DMSO control.

B. Absolute average expression (probe fluorescent intensity) between all expressed genes (n=8,095) and Th1-specific genes (n=291). P>0.05, unpaired Student's t-test.

C. Distribution of T-bet ChIP-seq signal (input-subtracted total reads) across 5,804 T-bet Th1 enhancers in mice from duplicate ChIP-seq experiments, generated by ROSE (Hnisz et al., 2013). T-bet occupancy is not evenly distributed across the enhancer regions, with a subset of enhancers to the right of the point of inflection (the 522 super-enhancers) containing especially high amounts of T-bet.

D. Left panel: Change in gene expression in Th1 cells treated with Flavopiridol (1 μM) versus JQ1 (50 nM). Middle panel: Change in gene expression in Th1 cells treated with 1 μM Flavopiridol versus 10 μM Flavopiridol. Right panel: Change in gene expression in restimulated Th1 cells treated with 50 nM JQ1 versus 500 nM JQ1.

E. Expression of selected genes (relative to naïve cells) in Th1 cells treated with DMSO, JQ1 or Flavopiridol. Genes are divided into those repressed by Flavopiridol only, JQ1 only or both Flavopiridol and JQ1.

Figure S4, related to Figure 4. T-bet is necessary for P-TEFb, Mediator and SEC recruitment to genes and enhancers in activated cells.

A. Co-immunoprecipitation of FLAG-T-bet and HA-cyclinT1 in 293 cells.

B. Co-immunoprecipitation of endogenous cyclinT1 with FLAG-T-bet in EL4 T cells. CyclinT1 is also precipitated by its P-TEFb partner CDK9, as expected. Suz12 is not precipitated by FLAG-T-bet or CDK9, and serves as a negative control.

C. Co-immunoprecipitation of endogenous cyclinT1 with FLAG-T-bet in primary mouse Th0 cells.

D. Cumulative distribution frequency of the change in P-TEFb, Aff4, Med1, Brd4, and RelA occupancy between EL4-T-bet and EL4-GFP cells at super-enhancers and associated genes (SE), at typical enhancers and associated genes (Typical) and at other sites (Other).

E. ChIP-seq binding profiles for T-bet, P-TEFb, RelA, Brd4, Med1 and Aff4 in EL4 cells stably expressing GFP alone or expressing T-bet and GFP and stimulated with PMA and ionomycin.

F. As B., except for WT and T-bet$^{-/-}$ Th1 cells restimulated with PMA and ionomycin.

G. RelA binding (versus input, mean and SD, n=3 technical replicates) at the indicated genomic locations in PMA + ionomycin stimulated EL4 cells expressing wild-type T-bet, T-bet S508A or GFP alone.

H. Top: Average binding profiles of P-TEFb, Aff4, Med1, Brd4 or RelA at the set of T-bet dependent sites for each factor in WT (darker colour) and KO (T-bet$^{-/-}$, lighter colour) cells. Bottom: Average P-TEFb binding profiles at the same locations shown above.

I Med1 and β-actin protein levels in Th1 cells transduced with retroviruses encoding shRNAs targeting luciferase and *Med1*. Due to a lack of specific antibodies, Med17 and Aff4 knockdown were confirmed at the RNA level only (Figures 4F and S4J).

J. Expression of *Med1* and *Med17* and the super-enhancer-associated genes *Furin, Dusp5, Xcl1, Csf2* and *Ccl3* relative to *Hprt* (mean and SD, n=3 technical replicates) in unstimulated and restimulated Th1 cells transduced with retroviruses encoding shRNAs targeting luciferase and Cre (white) or *Med1* and *Med17* (brown).

Figure S5, related to Figure 5. Restimulation and NF-κB activity are required for recruitment of the elongation machinery to T-bet target genes.

A. ChIP-seq binding profiles for P-TEFb at the *Bhlhe40* and *Picalm* loci in EL4-GFP and EL4-T-bet cells with and without restimulation with PMA and ionomycin.

B. ChIP-seq binding profiles for RelA, P-TEFb, Brd4, Med1 and Aff4 at the *BHLHE40* and *CCL3*/*CCL4* loci in restimulated human Th1 cells with and without treatment with BAY 11-7082 (20 µM).

Figure S6, related to Figure 6. eRNA transcription from super-enhancers at Th1 genes.

A. Cumulative frequency distribution of reads per kilobase per million total reads (RPKM) for total RNA in restimulated Th1 cells within T-bet binding sites and regions spanning 1 kb up- and downstream. T-bet binding sites are divided into those located within intergenic super-enhancers (n=269) or those within intergenic typical-enhancers (n=908). D=0.21, $P<10^{-3}$ (K-S test).

B. As A., except comparing total RNA reads around T-bet peaks within intergenic super-enhancers between restimulated Th1 and restimulated Th2 cells. D=0.21, $p<10^{-3}$ (K-S test).

C. As A., except comparing total and mRNA RNA reads around T-bet peaks within intergenic super-enhancers. D=0.17, p=0.001 (K-S test).

D. As A., except comparing total RNA reads around T-bet peaks within intergenic super-enhancers between unstimulated Th1 and restimulated Th1 cells. D=0.12, p=0.041 (K-S test).

E. As Figure 6A, except for total RNA purified from restimulated Th1 cells from a second donor.

F. Transcripts assembled by Cufflinks at the *IFNG* super-enhancer.

G. Top: Quantitative RT-PCR for the spliced and unspliced forms of NeST (Ifng-as1, Tmevpg1) relative to Hprt (mean and SD, n=3 technical replicates) in WT and T-bet-/- naïve mouse T cells and US and RS Th1 and Th2 cells polarised for 48 hrs or 7 days. Bottom: As Top, except for WT cells treated with 50 (+) or 500 nM (++) JQ1 (left) or treated with 1 (+) or 10 µM (++) Flavopiridol for 6 hours (right).

H. Total RNA and mRNA-seq data (reads/million) at *TBX21*, *FURIN* and *IFNG-AS1/IFNG* showing production of non-poly-adenylated RNAs from super-enhancers at Th1 genes. The strandedness of the RNA is indicated by + (Watson strand) or – (Crick strand). ChIP-seq binding profiles are shown above and are aligned with the RNA-seq data. The positions of super-enhancers are shown as red bars below.

Figure S7, related to Figure 7. **Reduction in retinal T cell infiltration and expression of inflammatory genes in CD4+ cells in mice treated with JQ1 and Flavopiridol.**

A. Number of CD3+ and CD45+ cells (mean and SD, n≥4) in three separate retinal fields in mice treated with carrier control, JQ1 or Flavopriridol. ** p<0.01, *** p<0. 001, **** p<0.0001 (2-sided t-test).

B. Number of CD3+ cells relative to the number of CD45+ cells in the samples described in A. * p<0.05, ** p<0.01 (2-sided t-test).

C. Representative cytofluorimetric analysis of retinal CD3+CD4+ T cells (for IFNγ, TNFα and FasL) and CD3+CD4+ T cells purified from the inguinal lymph node (for Ctla4 and Il18r1) from non-immunised and IRBP-immunised mice treated with carrier, JQ1 or Flavopiridol. Cells were permeabilized and stained intracellularly for detection of IFNγ, TNFα and Ctla4. Numbers adjacent to outlined areas indicate the percentage of cells.

D. Representative cytofluorimetric analysis of Icam1 expression on CD3+CD4+ T cells purified from the inguinal lymph node after treatment with vehicle only, JQ1 (30 mg/kg) or Flavopiridol (1mg /kg).

E. Summary and quantification of cytofluorimetric data shown in (D). Each symbol represents an individual animal; horizontal lines indicate the mean and error bars denote SD. MFI, median florescence intensity. * p<0.05 (2-sided t-test).

F. Representative cytofluorimetric analysis of CD29 surface expression on CD4+ T cells purified from the inguinal lymph node after treatment with vehicle only, JQ1 (30 mg/kg) or Flavopiridol (1mg /kg).

G. Summary and quantification of cytofluorimetric data shown in (F). Each symbol represents an individual animal; horizontal lines indicate the mean and error bars denote SD. * p<0.05 (2-sided t-test).

SUPPLEMENTAL TABLES

Table S1. Th1 and Th2 genes identified by gene expression profiling (xls file), related to Figure 1

Worksheet 1: Genes significantly more highly expressed in human Th1 or Th2 cells after 13 days polarization (Agilent human G3 arrays). Worksheet 2: Genes significantly more highly expressed in human Th1 or Th2 cells after 28 days polarization (Affymetrix U133 plus 2 arrays).

Table S2. T-bet super-enhancers in human and mouse Th1 cells (xls file), related to Figure 2

Coordinates of human and mouse T-bet super-enhancers and the closest RefSeq gene TSS. Some super-enhancers are associated with more than one gene (gene TSS lie within super-enhancers).

Table S3. Genes exhibiting differential responses to JQ1 and Flavopiridol (xls file), related to Figure 3

Genes repressed in reactivated mouse Th1 cells upon by Flavopiridol but not JQ1, JQ1 but not Flavopiridol or by both compounds. Each gene set is provided in an individual tab of the table.

Table S4. P-TEFb, RelA, Med1, Aff4, Brd4 and H3K4me3 binding sites in EL4-T-bet and EL4-GFP cells (xls file), related to Figure 4

The number of sequencing reads at each binding site is given, along with the position of the sites relative to murine T-bet super-enhancers and their associated genes (genes with closest TSS), and typical enhancers and their associated genes.

Table S5. P-TEFb, RelA, Med1, Aff4 and Brd4 binding sites in WT and T-bet-/- Th1 cells (xls file), related to Figure 4

The number of sequencing reads at each binding site is given, along with the position of the sites relative to murine T-bet super-enhancers and their associated genes (genes with closest TSS), and typical enhancers and their associated genes.

Table S6. P-TEFb, RelA, Med1, Aff4 and Brd4 binding sites in restimulated human Th1 cells treated with DMSO or BAY 11-7082 (xls file), related to Figure 5

The number of sequencing reads at each binding site is given, along with the position of the sites relative to human T-bet super-enhancers and their associated genes (genes with closest TSS), and typical enhancers and their associated genes.

Table S7. T-bet super-enhancer eRNAs in human Th1 and Th2 cells (xls file), related to Figure 6

Coordinates of eRNAs identified in from total RNA-seq in restimulated Th1 cells and their fragments per kilobase per million total reads (FPKM) values from total and poly-A+ RNA-seq in unstimulated (US) and restimulated (RS) Th1 and Th2 cells. Super-enhancer IDs correspond to the nearest RefSeq gene TSS. Some super-enhancers are associated with more than one gene and some genes are associated with more than one super-enhancer (delineated A, B). eRNA transcripts within each super-enhancer are numbered individually.

SUPPLEMENTAL EXPERIMENTAL PROCEDURES

Cells

Purified T cells were obtained from buffy coats (National Blood Service) or blood samples from healthy volunteers. Informed consent was obtained from all subjects, and blood was collected and processed with the approval of and in accordance with the King's College Ethics Committee guidelines (06/Q0705/20). Naïve human CD4+ T-cells (CD4+CD45RA+CD45RO-CD25-CCR7+) were isolated by negative immunomagnetic selection (Miltenyi Biotec) followed by sorting using a FACSAria II (BD Bioscience). Cells were activated for 72 hours by plate bound anti-CD3 and anti-CD28 antibodies (2 µg/ml, BD Pharmingen) and were then cultured for 10 days with rhIL-2 (Biolegend, 10ng/ml). Conditions for T cell polarisation were: rhIL-12 (10 ng/ml, Biolegend) and anti-IL-4 (10 µg/ml, R&D) for Th1, rhIL-4 (10 ng/ml, Biolegend) and anti-IFN-γ (10 µg/ml, R&D) for Th2. For ChIP, cells were formaldehyde crosslinked on day 13 either before (unstimulated) or after (restimulated) treatment with PMA and ionomycin. For gene expression microarray analysis and RNA-seq, samples were taken from naïve cells and Th1 and Th2 cells before and after restimulation with anti-CD3 and anti-CD28 antibodies on days 7 and 13 after purification and this was performed for 3 separate donors (sample size standard for differential gene expression analysis). For experiments testing the role of NF-κB, cells were restimulated for 5 hours with PMA/ionomycin in the presence of 20 µM BAY 11-7082 (Calbiochem) or DMSO before fixation.

For H3K4me3 ChIP-seq, CD4+ naive T-cells were differentiated into either Th1 or Th2 cells *in vitro* for 28 days, as described (Cousins et al., 2002). Cells were activated for 4 hrs with 5 ng/ml PMA (Sigma) and 500 ng/ml ionomycin (CN Biosciences) and assessed for intracellular cytokine staining.

Naïve murine CD4+ T-cells (CD4+CD25-CD62LhighCD44low) were purified from pooled spleen and lymph node cell suspensions of WT and T-bet$^{-/-}$ mice by immunomagnetic selection (Miltenyi Biotec) followed by sorting. Cells were activated for 72 hours with plate-bound anti-CD3 and anti-CD28 monoclonal antibodies (both 2 µg/ml) and then cultured for 5 days in the presence of 20 ng/ml IL-2 (Biolegend). Conditions for T cell polarisation were: 20 ng/ml IL-12

(eBioscience) and 5 μg/ml anti-IL-4 (BioXCell) for Th1 or 20 ng/ml IL-4 (eBioscience) and 10 μg/ml anti-IFN-γ (BioXCell) for Th2. For gene expression analysis, cells were harvested before and after restimulation with anti-CD3/CD28 at 48 hours and 7 days.

Human CCR5+ memory Th1 cells were enriched with CD4 microbeads (Miltenyi Biotech) and CCR5+ Th1 (anti-CCR5, BD Biosciences) effector memory cells isolated by flow cytometry, activated with plate-bound anti-CD3 and anti-CD28 for 4 days, and expanded in media containing IL-2, as described (Messi et al., 2003).

EL4-GFP and EL4-T-bet cells were described in (Kanhere et al., 2012). EL4 cells expressing a form of T-bet insufficient in interaction with RelA were generated as described previously (Kanhere et al. 2012) using the coding region of T-bet containing a S508A point mutation (Hwang et al. 2005). Cells were either incubated with DMSO (unstimulated control) or stimulated with PMA (50 ng/ml) and ionomycin (1 μM) for 5 hours before formaldehyde crosslinking.

Mice used to harvest cells for ChIP and *in vitro* gene expression analysis

Wild type (WT) C57BL/6 mice were purchased from Charles River Laboratories International (Margate, UK). T-bet$^{-/-}$ mice (on a C57BL/6 background) were purchased from Taconic (Ejby, Denmark). Mice were bred in the Biological Services Unit at KCL (UK Home Office project license PPL/70/6792) or at Charles River Laboratories International (Margate, UK) analyzed between 6 and 12 weeks of age.

ChIP-seq

Sample preparation

ChIP was performed as described (Kanhere et al., 2012). Cells were crosslinked by the addition of one-tenth volume of fresh 11% formaldehyde solution for 20 minutes at room temperature before the reaction was quenched by addition of glycine. Cells were rinsed twice with 1xPBS and flash frozen in liquid nitrogen. Cells were lysed with non-ionic detergent, nuclei washed and then lysed with ionic detergent. For RNA pol II ChIP, an alternative set of lysis and wash buffers were

used (Rahl et al., 2010). Cells were sonicated on ice to solubilize and shear crosslinked DNA (24W for 10 x 30 second pulses using a Misonix Sonicator 3000). The resulting whole cell extract was cleared by centrifugation and then incubated overnight at 4°C with 100 µl of Dynal Protein G magnetic beads that had been pre-incubated with 10 µg of purified antibody or, for the case of T-bet, 10 µl of purified serum (see table below). Beads were washed 6 times with RIPA buffer and 1 time with TE containing 50 mM NaCl. Bound complexes were eluted from the beads by heating at 65°C with occasional vortexing and crosslinks then reversed in IP and input DNA by overnight incubation at 65°C. IP and input DNA were then purified by treatment with RNase A, proteinase K and phenol:chloroform extraction followed by ethanol precipitation.

H3K4me3 ChIP was performed on native chromatin. Chromatin was prepared by using the protocol of Feil and colleagues (http://www.epigenome-noe.net/researchtools/protocol.php?protid=2) with some minor modifications. Mono and dinucleosomal chromatin was recovered from nuclei treated with micrococcal nuclease (10U/µl for 7 mins) and chromatin quality assessed by agarose gel elecrophoresis and semi-quantitated using a nanodrop. ChIP for H3K4me3 and total H3 was performed with Protein G beads (Active Motif). DNA was purified by phenol/chloroform phase separation, ethanol precipitation, followed by clean up with Qiagen PCR purification columns.

Libraries were constructed from ChIP and input DNA by standard Illumina protocols, except that DNA in the range 150-350bp was gel-purified after PCR-amplification. The libraries were quantified using a Qubit and Agilent bioanalyzer, pooled and subjected to 35 or 50 bp single-end read sequencing with an Illumina GAIIx or HiSeq 2500 sequencer.

ChIP-seq datasets used in this study.

US – not restimulated before crosslinking, RS – restimulated with PMA/ionomycin before crosslinking.

Species	***Cells***	***Condition***	***Factor***	***Antibody***	***Accession (if previously published)***
Human	Th1	US	T-bet	9856 (custom) (Jenner et al., 2009)	GSM776557 GSM776555
Human	Th1	US	T-bet	SY4530 (custom)	
Human	Th1	US	Rabbit IgG	Abcam 46540	
Human	Th1	RS	H3K27ac	Abcam ab4729	
Human	Th2	RS	H3K27ac	Abcam ab4729	
Human	Th1	US	H3K4me3	Abcam ab8580	
Human	Th2	US	H3K4me3	Abcam ab8580	
Human	CCR5+ memory Th1	US	H3K4me3	Abcam ab8580	
Human	Th1	US	RNA pol II	Santa-Cruz N-20 (sc-899)	
Human	Th1	RS	RNA pol II	As above	
Human	Th2	US	RNA pol II	As above	
Human	Th2	RS	RNA pol II	As above	
Human	Th1	US	P-TEFb	Pool of CyclinT1 T-18 (sc-8127), C-20 (sc-8128), CDK9 H-169 (sc-8338) and CDK9 C-20 (sc-484)	
Human	Th1	RS	P-TEFb	As above	
Human	Th2	US	P-TEFb	As above	
Human	Th2	RS	P-TEFb	As above	
Human	Th1	RS and DMSO	RelA	Santa Cruz C-20 (sc-372). Replicated with Abcam ab7970	
Human	Th1	RS and BAY 11-7082	RelA	As above	
Human	Th1	RS and DMSO	P-TEFb	Pool of CyclinT1 T-18 (sc-8127), C-20 (sc-8128), CDK9 H-169 (sc-8338) and CDK9 C-20 (sc-484)	
Human	Th1	RS and BAY 11-7082	P-TEFb	As above	
Human	Th1	RS and DMSO	Aff4	Bethyl Laboratories (A302-539A)	
Human	Th1	RS and BAY 11-7082	Aff4	As above	
Human	Th1	RS and DMSO	Brd4	Bethyl Laboratories (A301-985A100)	
Human	Th1	RS and BAY 11-7082	Brd4	As above	
Human	Th1	RS and DMSO	Med1	Bethyl Laboratories (A300-793A)	
Human	Th1	RS and BAY 11-7082	Med1	As above	
Mouse	Th1	RS	T-bet	9856 (custom) (Jenner et al., 2009)	GSM998272 GSM998271

Mouse	Th1	RS	T-bet	SY4530 (custom)	GSM836124
Mouse	T-bet$^{-/-}$ Th1	RS	T-bet	9856 (custom) (Jenner et al., 2009)	GSM998273
Mouse	Th1	?	H3K4me3	Abcam ab8580	
Mouse	Th2	?	H3K4me3	Abcam ab8580	
Mouse	T-bet$^{-/-}$ Th1		H3K4me3	Abcam ab8580	
Mouse	Th1		H3K4me3		GSM361999
Mouse	Th2		H3K4me3		GSM362001
Mouse	WT Th1	RS	P-TEFb	CDK9 H-169 (sc-8338)	
Mouse	WT Th2	RS	P-TEFb	As above	
Mouse	T-bet$^{-/-}$ Th1	RS	P-TEFb	As above	
Mouse	EL4+GFP	US	P-TEFb	Pool of CyclinT1 T-18 (sc-8127), C-20 (sc-8128), CDK9 H-169 (sc-8338) and CDK9 C-20 (sc-484)	
Mouse	EL4+GFP	RS	P-TEFb	As above	
Mouse	EL4+T-bet	US	P-TEFb	As above	
Mouse	EL4+T-bet	RS	P-TEFb	As above	
Mouse	WT Th1	RS	Brd4	Bethyl Laboratories (A301-985A100)	
Mouse	T-bet$^{-/-}$ Th1	RS	Brd4	As above	
Mouse	EL4+GFP	RS	Brd4	As above	
Mouse	EL4+T-bet	RS	Brd4	As above	
Mouse	WT Th1	RS	RelA	Santa Cruz C-20 (sc-372)	
Mouse	T-bet$^{-/-}$ Th1	RS	RelA	As above	
Mouse	EL4+GFP	RS	RelA	As above	
Mouse	EL4+T-bet	RS	RelA	As above	
Mouse	WT Th1	RS	Med1	Bethyl Laboratories (A300-793A)	
Mouse	T-bet$^{-/-}$ Th1	RS	Med1	As above	
Mouse	EL4+GFP	RS	Med1	As above	
Mouse	EL4+T-bet	RS	Med1	As above	
Mouse	WT Th1	RS	Aff4	Bethyl Laboratories (A302-539A)	
Mouse	T-bet$^{-/-}$ Th1	RS	Aff4	As above	
Mouse	EL4+GFP	RS	Aff4	As above	
Mouse	EL4+T-bet	RS	Aff4	As above	
Mouse	EL4+GFP	RS	FLAG	Sigma (M2, F1804)	
Mouse	EL4+T-bet	RS	FLAG	As above	

A note about pooling antibodies for ChIP-seq

We used a pool of CDK9 and cyclin T1 antibodies for some P-TEFb ChIP-seq experiments. CDK9 and CyclinT1 are not known to function independently of P-TEFb and thus antibodies to both factors only detect P-TEFb with a single binding profile. Pooling antibodies to multiple subunits of the same factor maximizes the signal, and has been used previously by other investigators (eg. Zeitlinger et al., 2007). Pooled monoclonal antibodies in immunoprecipitations enable the formation of multimeric complexes, like polyclonal antibodies, but are more specific than polyclonal antibodies ("Using Antibodies" by Ed Harlow and David Lane).

A note about the Super-elongation complex

The super-elongation complex (SEC) has been extensively characterized (He et al., 2011; Lin et al., 2011; Lin et al., 2013; Lin et al., 2010; Luo et al., 2012; Mueller et al., 2009). Aff4 is a core, essential subunit of the complex, is not known to be present in other complexes, and has previously been used as a super-elongation complex marker in ChIP-seq (eg. Lin et al., 2011 and Luo et al., 2012).

ChIP-seq data replication

We assessed the quality and reproducibility of our dataset in three ways. Firstly, we performed biological replicate ChIP-seq experiments for T-bet, RNA pol II, P-TEFb, Aff4, Brd4, Med1 and RelA (Santa Cruz sc-372 and Abcam ab7970) in human Th1 cells and for T-bet and Aff4 (Bethyl A302-539A and that used in Lin et al., 2010, kind gift from Ali Shalatifard) in mouse WT and T-bet-/- Th1 cells. We assessed the consistency between the replicates by irreproducible discovery rate (IDR) analysis (Li et al., 2011) and found that, in each case, Np/Nt was less than 2, which is the standard reproducibility threshold used by the ENCODE project (Landt et al., 2012). Secondly, for every ChIP-seq experiment, the specific enrichment of binding sites, along with their T-bet or NF-kB dependence was validated by ChIP-qPCR. Finally, ChIP-seq for T-bet, P-TEFb, RelA, Brd4, Med1 and Aff4 was performed in human Th1, mouse Th1 and mouse EL4-T-bet cells, and in each case, the factors displayed the same characteristic binding profiles regardless of the cell type or species, further confirming the robustness of the data.

ChIP-Chip for the initiation form of RNA polymerase II

ChIP was performed for the initiation form of RNA pol II using the antibody 8WG16 (Abcam ab817) using our standard protocol. ChIP and input DNA were then amplified, labelled and hybridised to custom oligonucleotide microarrays (Agilent) covering 8 kb (approximately 4 kb upstream and 4 kb downstream) around the transcription start site of 18,450 Ref-Seq-annotated human genes, as described (Jenner et al., 2009). Fold enrichment of DNA in ChIP versus input was calculated using a previously described analysis pipeline (Jenner et al., 2009).

ChIP-seq data analysis

Reads (in fastq files) were filtered to remove adapters using fastq-mcf and for quality using seqkt and aligned to the human (hg19) or mouse genome (mm9) with Bowtie2 (default settings). Bigwig files for visualization in the UCSC genome browser were generated using a custom pipeline; duplicate reads were first removed, coverage calculated with genomeCoverageBed and tag density calculated in 10bp windows. unionBedGraphs was then used to subtract input (or total histone H3 for histone ChIPs) signals and bigwig files generated using bedGraphToBigWig. Regions of significant enrichment were identified using MACS version 1.4 (Zhang et al., 2008) using input or total histone H3 as background, with the setting --keep-dup=1. A p-value threshold of 10^{-7} was used, unless stated otherwise. Binding sites within 2 kb of any RefSeq gene transcription start site (TSS) were identified using closestBed and considered as proximal binding sites and associated with that gene. Other binding sites were divided into those within a RefSeq gene (intragenic) and those outside of a gene (intergenic) using intersectBed.

Numbers of proximal, intergenic and intragenic binding sites ($p<10^{-7}$, associated with Figure 2C). The relative distribution of P-TEFb binding sites is also maintained at more stringent MACS p-value thresholds.

Factor or histone modification	***Proximal (<2kb from TSS)***	***Distal intragenic***	***Distal intergenic***	***Total sites***
**RNA pol II**	11731	9813	4261	25805
**H3K4me3**	14687	4485	4936	24108
**H3K27ac**	11467	11194	7216	29877
**P-TEFb**	1865	2131	2147	6113

Average binding profiles

Average binding profiles (in reads/million) across sets of genes or enhancers were generated with ngsplot (https://code.google.com/p/ngsplot/ (Shen et al., 2014)) and reads from input or control total H3 ChIPs subtracted. To control for differences in ChIP efficiency when comparing average profiles between ChIPs of the same factor in different cells, average reads/million across a set of genes were then normalized by the average signal across all genes. The relative levels of RNA pol II at Th1 and Th2 genes between Th1 and Th2 cells were calculated by dividing the two ngsplot average profiles.

To determine change in P-TEFb binding at intergenic sites between WT and T-bet$^{-/-}$ cells (Figure 4A), intergenic sites were first defined by MACS as those with $p<10^{-9}$ in WT Th1 cells and located outside of RefSeq genes and satellites and simple repeats (RepeatMasker). Then, ngsplot was used to calculate P-TEFb binding profiles across each site and the maximum value (the peak height) identified. The same analyses method was used to determine change in P-TEFb binding at intergenic sites between stimulated EL4-GFP and EL4-T-bet cells (Figure 4B), except that the set of intergenic P-TEFb sites in stimulated EL4-T-bet cells was used.

Counts at specific genes or proximal regions

The numbers of reads at specific genes were counted using featureCounts (http://bioinf.wehi.edu.au/featureCounts/ (Liao et al., 2014)) and converted to reads per kilobase per million (RPKM) using the size of the feature and the total number of aligned reads. The number of reads in the input sample were then subtracted. To compare P-TEFb with RNA pol II (Figure S2G), reads across the longest variant of each RNA pol II or P-TEFb-bound gene were counted. To compare P-TEFb binding in the presence or absence of T-bet (Figure 4A-B), reads were counted over regions spanning -100 to +500bp relative to TSS that were considered bound by P-TEFb by MACS ($p<10^{-7}$).

Identification of super-enhancers

Super-enhancers are genomic regions that exhibit high levels of binding by a particular transcriptional regulator. They were first identified by Whyte and colleagues (2013) and Loven and colleagues (2013) for Med1. Super-enhancers have also been identified by the binding of single site-specific transcription factors, for example PU.1 in pro B-cells and MyoD in myotubes by Whyte et al. Super-enhancers bound by T-bet in Th1 cells were identified with the ROSE algorithm (Loven et al., 2013; Whyte et al., 2013; Hnsiz et al., 2013; https://bitbucket.org/young_computation/rose).

The ROSE algorithm seeks to identify regions of the genome with high levels of binding by a particular transcriptional regulator. All the sites bound by the factor of interest are identified by a standard tool, such as MACS, and then those sites lying within 12.5 kb of one another are grouped together to identify bound regions (not including sites that lie within 2.5 kb of gene TSS). Then the numbers of sequencing reads for each binding site (typical enhancer) or grouped binding sites (super-enhancer) are counted. The regions are then ordered by the number of ChIP sequencing reads ("binding") they contain and the number of reads plotted against rank. This produces a curve with an exponential profile whereby a minority of genomic regions have many more sequencing reads (and thus binding of the transcriptional regulator) than others. The threshold used by the ROSE algorithm to define super-enhancers is the point of inflexion in the relationship between enhancer rank and transcription regulator occupancy (measured by ChIP-seq reads), as shown in Figure S2A (for human) and S3C (for mouse).

Human T-bet. For human, T-bet binding sites were identified from two biological replicate ChIPs, performed using two independent antibodies, using MACS, which identified 10,358 sites at $p<10^{-7}$ for one replicate and 18,219 sites $p<10^{-9}$ for the other (after removal of a small number of peaks at satellites using intersectBed). IntersectBed was then used to identify binding sites identified in both replicates (4,821 sites) and ROSE employed with its default settings to identify enhancers by stitching together binding sites located within 12.5 kb of each other that were at least 2.5 kb from RefSeq-annotated transcription start sites. This resulted in identification of 3191 enhancers. Super-enhancers were then identified by ROSE as those with highest number of reads in the two T-bet replicate ChIP datasets (using a merged Bam file), in comparison to input

sequencing data, as described in (Whyte et al., 2013). The plot of sequencing reads for each stitched T-bet enhancer is shown in Figure S2A. This resulted in identification of 374 T-bet super-enhancers and 2,817 typical enhancers in human Th1 cells. Enhancers were deemed to be associated with the nearest gene TSS (Whyte et al., 2013), identifying 357 genes. To increase confidence in our gene assignment further for Figures S2D and S2E, we used the closest genes that also had a proximal T-bet binding site (MACS $p<10^{-7}$, from Bam files merged from both replicate ChIPs). For humans, this identified 219 genes associated with super-enhancers and 1041 genes associated with typical enhancers.

Mouse T-bet. For mouse, T-bet binding sites were identified from two biological replicate ChIPs, one from our own lab and one from (Nakayamada et al., 2011) (accession GSM836124 at GEO), each performed using different antibodies. MACS identified 13,644 ($p<10^{-7}$) and 32,770 ($p<10^{-9}$) binding sites, respectively. More sites were identified for GSM836124 because no input dataset was available for MACS to use as background. 10,094 sites were present in both datasets (after removal of satellites) and ROSE identified 5282 typical enhancers and 522 super-enhancers (Figure S3C). Mapping these to the closest TSS identified 471 genes.

Measuring changes in transcription regulator occupancy

Changes in the binding of transcriptional regulators between WT and T-bet-/- Th1 cells or between EL4-T-bet and EL4-GFP cells were measured using a cumulative distribution frequency analysis. For each transcriptional regulator, binding sites were identified by MACS and filtered to remove any overlapping satellites, ENCODE blacklist regions (https://sites.google.com/site/anshulkundaje/projects/blacklists) or sites for which at >25% corresponded to a simple repeat. For mouse, sites on the Y chromosome were removed because of differences between the ratio of male and female cells in the different cell lysates. MAnorm (Shao et al., 2012) was then used to merge peaks between the two samples under comparison (eg. merge the peaks for Aff4 between WT and T-bet KO). Using BEDtools, we then identified the binding sites for each factor that overlapped T-bet super-enhancers or their associated (closest) genes (“SE” sites), binding sites that didn’t fall into this category but overlapped typical T-bet enhancers (stitched enhancers not classified as super-enhancers by ROSE and their closest genes (“Typical” sites) and then any other binding site outside of these T-bet-associated regions

("Other" sites). Read numbers under each merged peak were converted to reads per million total reads and log2 ratios calculated (T-bet$^{-/-}$ vs WT, EL4-T-bet vs EL4-GFP or BAY 11-7082 vs DMSO). The cumulative distribution frequency of these changes within each set of sites was then plotted. Thus, for the T-bet$^{-/-}$ vs WT analysis, the further the "SE" and "Typical" lines are shifted to the left relative to the "Other" line, the greater the loss of the factor at T-bet target sites compared to non-T-bet target sites (at which one expects binding to remain constant upon T-bet loss). The significance of the differences in log2 ratios between the different binding site sets (super vs other; typical vs other) were estimated with R using a Mann-Whitney U test and corrected for multiple hypothesis testing by multiplying by the number of transcriptional regulators under test for each cell type.

MAnorm was also used to identify sites at which transcriptional regulators were significantly depleted in T-bet$^{-/-}$ cells versus WT Th1 cells with a threshold of $p<0.001$ and with a fold-change of at least 3-fold. The binding profile of each factor and of P-TEFb at these sites was then plotted with ngsplot (Figure S4H).

Gene expression microarray analysis

Human Th1 and Th2 cells

Total RNA was purified with Trizol (Life Technologies) from naïve and *in vitro* polarised Th1 and Th2 cells (3 different donors) at days 7 and 13 after purification before and after restimulation. RNA was DNase-treated using DNA-free (Life Technologies) and integrity verified using an Agilent Bioanalyzer. Fifty ng of RNA was labelled with Cy3 and RNA from a common reference RNA pool (Stratagene) was labelled with Cy5 using Agilent's Low input Quick Amp 2 Color Labelling kits and hybridized together to Agilent Human gene expression G3 DNA microarrays, following the standard protocol. Arrays were scanned on an Agilent High Resolution C scanner and images were quantified using Agilent Feature Extraction Software (version 10.7). Genes significantly higher expressed across all human Th1 cell samples versus all Th2 cell samples, or vice versa, were identified by rank sum test (pfp<0.05) using RankProdIt (http://strep-microarray.sbs.surrey.ac.uk/RankProducts/ (Laing and Smith, 2010)). Th1 and Th2

gene sets were also filtered to only include those at least 2-fold upregulated versus naïve cells in all Th1 samples or all Th2 samples, respectively.

We also polarized human CD4+ T cells for 28 days and performed gene expression microarray analysis and H3K4me3 ChIP-seq. RNA was purified from cells with and without restimulation from 3 donors and labeled and hybridized to GeneChip U133 plus 2 arrays according to the manufacturer's instructions (Affymetrix). Robust multichip average preprocessing was performed and genes significantly overexpressed in Th1 or Th2 cells were identified using the Partek ANOVA model (>2-fold difference, $p<0.05$ including FDR).

To provide an independent data set, we also plotted RNA pol II and H3K4me3 ChIP-seq data at Th1 and Th2 genes from Tables S4A in (Hawkins et al., 2013) (Figure S1G).

In vitro treatment with Flavopiridol and JQ1 and microarray analysis

Mouse wild-type naive CD4+ T cells were cultured under Th1 and Th2 polarizing conditions. On day 6 of polarization, $4x10^6$ cells were transferred in medium complemented with 50 nM and 500 nM JQ1 (kindly provided by Jay Bradner) or 1% DMSO vehicle control. On day 7 of polarisation previously untreated cells were cultured in the presence of 50 nM and 500 nM JQ1, 1 μM and 10 μM Flavopiridol or 1% DMSO vehicle control for 2 hrs. These cells and the cells incubated with the drugs for 22 hrs were reactivated with plate-bound αCD3/CD28 (2 μg/ml each) or left unstimulated for 4 hrs. Total RNA was purified with TRIsure (Bioline), DNase-treated using DNA-free (Life Technologies) and integrity verified using an Agilent Bioanalyzer. Two hundred ng total RNA isolated from mouse Th1 and Th2 cells treated with Flavopiridol, JQ1 or DMSO was labeled with Cy3 with the two color Low Input Quick Amp Labeling Kit (Agilent) according to the manufacturer's instructions (with naïve T cell reference RNA labeled with Cy5) and both hybridized together to SurePrint G3 Mouse GE 8x60K microarrays (Agilent). Arrays were scanned as before. Median raw intensity values were background corrected using a normal-exponential convolution model and log2 transformed expression ratios (Cy3 channel vs trimmed mean of Cy5 channel across all arrays) were loess normalised. Expression ratios were first averaged for identical probes and then for identical genes. Genes with probe signal intensities less than 40% higher than the 95th percentile of the negative control probes were removed from the analysis and the remaining 8095 genes were considered to be expressed across the data set.

Lineage-specific genes and groups of genes repressed by JQ1 and/or Flavopiridol in reactivated Th1 cells were defined using the following thresholds: Th1-specific: Th1 DMSO 6 hrs vs Th2 DMSO 6hrs >= 2 (n =291); Th2-specific: Th1 DMSO 6hrs vs Th2 DMSO 6 hrs <= 2 (n= 180); Flavopiridol-specific genes: DMSO 6 and 24 hrs vs naïve >= 2, 1 µM and 10 µM Flavopiridol < 2, 1 µM and 10 µM Flavopiridol vs DMSO 6hrs <= 2, 50 nM and 500 nM JQ1 6hrs vs DMSO 6 hrs >= 1 (n= 126); JQ1-specific genes: DMSO 6 and 24 hrs vs naïve >= 2, 500 nM JQ1 6hrs vs DMSO 6hrs <= 1.5, 500 nM JQ1 24hrs vs DMSO 24hrs <= 1.5, 1 µM and 10 µM Flavopiridol vs DMSO 6hrs >= 1 (n= 147), genes repressed by Flavopiridol and JQ1: DMSO 6 and 24 hrs vs naïve >= 2, 1 µM and 10 µM Flavopiridol vs DMSO 6hrs <= 2, 500 nM JQ1 6hrs vs DMSO 6hrs <= 1.5, 500 nM JQ1 24hrs vs DMSO 24hrs <= 1.5 (n= 247). Significance of differences in gene expression between groups of genes presented in the cumulative distribution frequency plots was determined by a two-tailed Kolmogorov-Smirnov (K-S) test.

Comparison of CD4+ T cell gene expression to other cell-types

Data profiling gene expression across 79 human cell and tissue types was obtained (Su et al., 2004) and gene expression in CD4+ T cells calculated relative the median level across all cell types. These values were then plotted as a cumulative distribution for genes associated with a T-bet super-enhancer and proximal binding site and genes associated with a typical T-bet enhancer and proximal binding site and the significance of the difference between them estimated using a K-S test.

Strand-specific RNA-seq

Sample preparation

Total RNA purified from naïve CD4+ cells and day 13 resting and restimulated Th1 and Th2 cells (2 donors) was obtained from samples also used for microarray analysis. Poly-adenylated RNA was purified from 1 µg of total RNA using the Qiagen Oliogtex kit and ribosomal RNA was depleted from 1 µg of total RNA using Ribo-Zero Gold (EpiCentre), as per manufacturer’s instructions. 5’ cap structures were removed with tobacco acid pyrophosphatase (TAP, Life Technologies) and RNA fragmented with potassium acetate (100 mM) and magnesium acetate (30 mM) at $94^{o}C$ for 3 mins. RNA was then repaired with Antarctic phosphatase and PNK,

according to the instructions in the Illumina Directional mRNA-seq Sample Prep Guide. Libraries were generated using the NEBNext Multiplex Small RNA Library Prep Set for Illumina kit, with 14 cycles of PCR amplification, and products between 130bp and 350bp were gel purified. Libraries were quantified by qPCR (Library Quantification Kit, Kapa Biosystems) and average fragment size determined on an Agilent 2100 Bioanalyzer using DNA HS assays. Libraries were sequenced on an Illumina HiSeq (50bp paired-end).

Processing and alignment of RNA-seq data

Reads (in fastq files) were filtered to remove adapters using fastq-mcf (-S, -t 0.0001, -l 20) and for quality using seqkt trimfq (-q 0.01) and aligned to human genome (hg19) with TopHat2 (v2.0.9, --b2-very-sensitive, -g 2, -p 4, --library-type fr-secondstrand, default setting for –mate-inner-dist and --mate-std-dev). Bigwig files for visualization of the data in the UCSC Genome browser were generated from TopHat2 accepted hits bam files using Samtools to select for strand specific reads, which were then converted into Bedgraph summaries using Bedtools genomecov, prior to final conversion to BigWig format using bedGraphToBigWig from UCSC. Transcripts were reconstructed using Cufflinks v2.1.1, with default settings (masking for rRNA, snRNA, Mt_rRNA, snoRNA and miRNA listed in Gencode v19 gene annotation). Output from Cufflinks was filtered to remove transcripts overlapping known gene structures. Putative eRNAs were identified from the remaining list using Bedtools intersect to select for mono-exonic transcripts overlapping T-bet super-enhancers.

Quantification of RNA production at enhancers

RNA-seq read coverage at each enhancer was calculated using featureCounts (http://bioinf.wehi.edu.au/featureCounts/ (Liao et al., 2014)). Read count was normalized to the total number of aligned reads per library and size of the T-bet binding site (kb). A 1 kb region up and downstream was appended to each enhancer coordinate to capture RNA-seq reads pertaining to the enhancer but extending beyond the T-bet boundary. Significance of differences in read number between T-bet sites in super-enhancers and enhancers or between different RNA samples was estimated using a K-S test.

Average RNA-seq read profiles around T-bet binding sites (identified in both replicates) that lied within super-enhancers or typical enhancers were calculated using ngsplot (https://code.google.com/p/ngsplot/ (Shen et al., 2014)). Enhancers overlapping multi-copy non-coding RNAs (tRNA, rRNA and snRNA, coordinates from RepeatMasker) as well as those within 2 kb of known protein-coding and non-coding genes (annotated by RefSeq and Gencode v19) were removed using intersectBed to ensure that previously annotated classes of RNAs were not being measured.

For comparison of FPKMs of individual eRNAs between Th1 and Th2 conditions, eRNA coordinates were defined in the Th1 RS total RNA library using Cufflinks. Reads corresponding to cufflink transcripts from comparison conditions were included if transcripts showed at least 1% overlap. Transcripts overlapping repeats (tRNA, rRNA and snRNA) or RefSeq annotated genes were removed from analysis. Putative eRNAs overlapping Ensembl genes but not annotated by RefSeq were assessed individually and manually removed if evidence of annotated gene transcription existed in our libraries. Multi-exonic transcripts were also excluded.

Processing of published RNA-seq data

Data from Wei and colleagues (GSM523209, GSM523211, GSM661236 and GSM661238 (Wei et al., 2011) was quality filtered and aligned as described above. Genes significantly overexpressed in Th1 cells compared to Th2 cells, and vice-versa, were identified using Cufflinks, with $p<0.05$ and log2 FC>2 thresholds. Previously identified sets of murine Th1 and Th2 genes were also taken from Additional file 1 of (Stubbington et al., 2015).

Gene Ontology

For functional analysis, sets of genes were uploaded to DAVID (http://david.abcc.ncifcrf.gov) and enriched Biological Process categories identified. Gene sets used were genes associated with T-bet super-enhancers and a proximal T-bet binding site and genes associated with typical T-bet enhancers and a proximal binding site (Figure S2E) and genes only occupied by P-TEFb in restimulated Th1 cells at proximal sites and genes occupied by P-TEFb at both proximal sites and intergenic sites (Figure 2F).

Quantitative reverse-transcription PCR

Total RNA was purified from cells with Trizol, DNase-treated with DNA-free (Life Technologies) and the quality routinely verified using an Agilent bioanalyzer. RNA was reverse transcribed with SuperScriptIII (Life Technologies) primed with random primers. No-RT controls reactions were also performed for all eRNA qPCRs. The abundance of mRNAs and eRNAs was measured relative to Hprt by quantitative PCR using QuaniTect SYBR green (Qiagen) and a Applied Biosystems 7500 machine using the dCt method.

qPCR primers used in this study

Target	*Species*	*F primer*	*R primer*
Ifng	Mouse	GCCAAGTTTGAGGTGAGACG	GTGGACCACTCGGATGAGC
IFNG	Human	AAACGAGATGACTTCGAAAAG	ACAGTTCAGCCATCACTTGG
Med1	Mouse	GCGAGCACCCTTCTCTTCTTG	GCCTCTCTGAGTCCTCGGTT
Med17	Mouse	ACAGACATTGACTTGGATAAGAAGATAC	TGAATAGAAACCTTGATATACGCAGAC
Aff4	Mouse	AGCAAAGCACATCTCACCAA	AATGCGTCATCTCTTTAAGTATTTC
Furin	Mouse	TTGGATGGCGAGGTGACTGATG	GCTGTAGATGTGGATGTGGTTGG
Dusp5	Mouse	CTGAGTGCTGTGTGGATGTGAAG	CTGGTCATAGGCTGGTCTGTAGG
Xcl1	Mouse	AGACTTCTCCTCCTGACTTTCCT	CTTCAGTCCCCACACCTTCCAC
Csf2	Mouse	CGCTCACCCATCACTGTCACC	GACGACTTCTACCTCTTCATTCAACG
Ccl3	Mouse	CCAAGTCTTCTCAGCGCCATAT	GCCGGTTTCTCTTAGTCAGGAAAATGA
Ccl4	Mouse	GCTTTGTGATGGATTACTATGAGACC	CTCCTGAAGTGGCTCCTCCTG
Nest unspliced	Mouse	AATTGTGGTCGTTGTGTCTCC	GCCTGGGTTTCTGATACAGC
Nest spliced	Mouse	ATGCTAATTAAACAGAGTACCCGT	AACAAGAGTTACTGAAGCTGGA
Hprt	Mouse	TCAGTCAACGGGGGACATAAA	GGGGCTGTACTGCTTAACCAG
HPRT	Human	AGTCTGGCTTATATCCAACACTTG	GACTTTGCTTTCCTTGGTCAGG
Ifng eRNA -40kb	Mouse	AGCTCCCATTAATGACACACC	GTGGTAACACACACACACACC
Ifng eRNA -38.7kb	Mouse	AAAAGCCCAGAGTGTCAACC	GCTCTTCTCTTTCCAAGAAGC
Ifng eRNA -29kb	Mouse	CAAGGGTTGAGAATGGGTGC	TTGAAGATCACTCCTGCAAGT
Ifng eRNA -26kb	Mouse	TCCGTGTGACATGTCGTTTAG	AACAGAAGCCCTGCATTTTG
Ifng eRNA -10kb	Mouse	TTCTGCAGGCTCACTATTGG	GTGCGCTGCCTGTAAAGC

Experimental autoimmune uveitis and treatment with Flavopiridol and JQ1 *in vivo*

EAU induction

B10.RIII mice were housed at the Biological Service Unit, UCL Institute of Ophthalmology, in individually ventilated cabinets in specific pathogen-free conditions, according to UK Home Office Regulations. All animal studies were ethically reviewed and carried out in accordance with Animals (Scientific Procedures) Act 1986, Welfare and Treatment of Animals. B10.RIII mice aged 5-8 weeks (>20g body weight) were immunized subcutaneously in the flank with 300 μg $IRBP_{161–180}$ (SGIPYIISYLHPGNTILHVD, Cambridge Peptides) in PBS emulsified with Complete Freund's adjuvant (CFA) (Sigma) supplemented with 1.5 mg/ml *Mycobacterium tuberculosis* complete H37 Ra (Difco Microbiology) (1:1 v/v). Each mouse also received 0.4 μg *Bordetella pertussis* toxin (Sigma) intraperitoneally (Agarwal et al., 2012).

Treatments

Mice were treated with JQ1 (3, 30 mg/kg), Flavopiridol (3, 15 mg/kg) or vehicle control once daily by intraperitoneal injection for 5 consecutive days. JQ1 (kindly provided by James Bradner) and Flavopiridol (Sigma) were dissolved in DMSO and mixed with 10% hydroxypropyl-β-cyclodextrin (Sigma) in sterile water to improve solubility. Vehicle treated mice received equivalent volumes of DMSO mixed with 10% hydroxypropyl-β-cyclodextrin. A sample size of 7 mice per group was chosen based on a high reproducibility in EAU disease scores across the groups, and availability of mice within the age range from the colony used. Mice were only excluded from the study if, after 8 days post immunization, no signs of retinal inflammation were detectable by retinal imaging. This was less than 10% of the total number immunized for all experiments.

Fundus imaging

On day 8-9 post immunization, *in vivo* imaging of retinal fundus was performed (Micron III retinal imaging microscope, Phoenix Research Labs) to screen for early signs of retinal inflammation (grade 1). Only those showing positive signs were selected for use. The day before termination (day 14-15), retinal disease progression was monitored and scored by retinal fundoscopy, using an established scoring system (Agarwal et al., 2012).

Ocular dissection

Enucleated eyes were fixed in 4% glutaraldehyde for 2 hours, followed by overnight fixation in formalin. Eyes were then processed, orientated and embedded for paraffin wax sectioning and cut in 4-5 μm sections, stained with eosin and counterstained with hematoxylin. The sections were scored using an established grading system (Agarwal et al., 2012). The significance of the difference between drug treated and untreated mice was measured using an unpaired t-test with Welch's correction, two-tailed (Gardner et al., 2013). Cell counting was performed on rehydrated paraffin-embedded sections (3-4 μm), using rat anti-mouse CD45 monoclonal antibody (1:400; Serotec) or rabbit anti-mouse CD3 antibody (1:400; Santa Cruz), followed by DAB (Vector Lab, Peterborough, UK), and counterstained with hematoxylin. Positive cells were counted in a minimum of three separate retinal fields, and data shown as means ± SD (n>4 mice per group).

For retinal cells, enucleated eyes were dissected in 100 μl of cold DMEM media. Following incision at the limbus with a 29G needle, a circumferential cut was made. Iris was dissected away releasing anterior chamber infiltrating cells into the dissection media. The retina was then removed from the eye cup leaving the Sclera/RPE/choroid intact. The dissection media and retina were then pipetted up into a 1.5ml tube and mechanically disrupted by vortexing to obtain a single cell suspension followed by centrifugation through a single well of a 96 well 60 μm cell strainer plate (Millipore). The resulting cell pellets were re-suspended and stained for immunophenotyping.

Peripheral lymph node dissection

The inguinal lymph nodes were dissected asceptically and cell suspensions were prepared as previously described (Gardner et al., 2013).

Flow cytometry

Single cell suspension were prepared from the retinas and inguinal lymph nodes and cells were stimulated with PMA (50 ng/ml) and ionomycin (1 μM) in the presence of 3 μM monensin for 4 hours. Surface markers were stained in PBS with 1% BSA for 20 min on ice, then the cells were fixed with 1% formaldehyde, permeabilized with 0.1% saponin in PBS and stained with fluorophore-conjugated antibodies in permeabilization buffer. For the detection of Ctla4, the cells

were first stained on the cell surface and, after permeabilization, the staining was repeated intracellularly. Fluorophore-conjugated antibodies used were: AlexaFluor700 anti-CD4 (RM4-5, BD Biosciences), phycoerythrin-cyanine 7 anti-CD3 (145-2C11, eBiosciences), eFluor 450 anti-IFN-γ (XMG1.2, eBiosciences), Fluorescein isothiocyanate anti-TNFα (MP6-XT22, eBiosciences), phycoerythrin anti-FasL (MFL3, eBiosciences), phycoerythrin anti-Ctla4 (UC10-4F10-11), Fluorescein isothiocyanate anti-IL18R1 (112614, R&D), Fluorescein isothiocyanate anti-ICAM1 (YN1/1.7.4, eBiosciences) and Peridinin-chlorophyll-eFlour 710 anti-CD29 (HMb1-1, eBiosciences). Data were collected using an LSRFortessa (BD Biosciences) then analyzed with Flow Jo software (Treestar).

shRNA knock-downs

To express shRNAs in mouse T cells, a miR-30 based shRNA expression element was fused to the 3' end of the reporter genes Thy1.1 and TurboRFP (tRFP). These reporter-shRNA cassettes were subsequently inserted into the pMY retroviral expression vector to create pMY-Thy1.1-miR30 and pMY-tRFP-miR30. The shRNA sequences targeting Med1 and Med17 have previously been described (Van Essen et al., 2009; Kagey et al., 2010). shRNAs targeting the firefly luciferase and cre recombinase genes were used as a negative controls. All shRNA sequences used are listed below.

shMed1: CGCAAGCACAAATTCTTCTAA

shMed17: AGAGATGGTCGGGTAATCA

shAff4: GCAACATTCAAGTCAGTCTTT

shFLuc: GGTGGCTCCCGCTGAATTGGA

shCre: GTGGGAGAATGTTAATCCATA

Retroviral particle preparation and infection were performed as previously described (Kanhere et al. 2012). Thy1.1+ cells were isolated on day 4 of Th1 polarization using CD90.1 magnetic beads (Miltenyi). For Med1/Med17 double knock-down, the cells were transduced with a mixture of pMY-Thy1.1-shMed1 and pMY-tRFP-Med17 viral particles and Thy1.1+tRFP+ double positive cells were purified by flow cell sorting on day 6. On day 7 the cells were either restimulated with 2 μg/ml anti-CD3/CD28 or left unstimulated for 6 hrs. Med1, Med17 and Aff4 knockdown were

confirmed by qRT-PCR. Med1 knockdown was additionally confirmed by western blotting (Figure S4I). No suitable antibodies for western blotting were available for Med17 and Aff4.

Co-immunoprecipitation and immunoblot analysis

HEK293 cells were transiently transfected with FLAG-T-bet and HA-CyclinT1 or HA-Cdk9 expression vectors using polyethyleneimine. Cells were lysed in RIPA buffer (50 mM Tris, pH 8.0, 150 mM NaCl, 1 mM EDTA, 1% IGEPAL CA-630, 0.5% sodium-deoxycholate, 10% glycerol and Complete protease inhibitors (Roche)) 48 hrs after transfection. For co-immunoprecipitation of FLAG-T-bet with endogenous Cyclin T1 and Cdk9, EL4-FLAG-T-bet cells were stimulated for 5 hrs with PMA and ionomycin, followed by lysis in RIPA buffer described above. Whole-cell lysates were immunoprecipitated at 4 °C with anti-FLAG (M2; Sigma), anti-Cyclin T1 (H-245, Santa Cruz), anti-Cdk9 (C-20, Santa Cruz), mouse polyclonal IgG (sc-2025, Santa Cruz) or rabbit polyclonal IgG (P120-101; Bethyl) coupled to magnetic protein-G beads (Dynal). Immune complexes were washed once with wash buffer (40 mM Tris, pH 7.5, 150 mM NaCl, 0.3% IGEPAL CA-630 and 10% glycerol), five times with wash buffer containing 500 mM NaCl and once with HD buffer (20 mM Tris, pH 8.0, 150 mM NaCl and 10% glycerol), reuspended in Laemmli buffer and incubated at 95°C for 5 min. Proteins were resolved on a 8% Tris-glycine gel and transferred to a nitrocellulose membrane. Antibodies for immunoblotting were identical to those used for immunoprecipitation, in addition to anti-Suz12 (P15, Santa Cruz).

SUPPLEMENTAL REFERENCES

Cousins, D.J., Lee, T.H., and Staynov, D.Z. (2002). Cytokine coexpression during human Th1/Th2 cell differentiation: direct evidence for coordinated expression of Th2 cytokines. Journal of immunology *169*, 2498-2506.

He, N., Chan, C.K., Sobhian, B., Chou, S., Xue, Y., Liu, M., Alber, T., Benkirane, M., and Zhou, Q. (2011). Human Polymerase-Associated Factor complex (PAFc) connects the Super Elongation Complex (SEC) to RNA polymerase II on chromatin. Proc Natl Acad Sci U S A *108*, E636-645.

Jenner, R.G., Townsend, M.J., Jackson, I., Sun, K., Bouwman, R.D., Young, R.A., Glimcher, L.H., and Lord, G.M. (2009). The transcription factors T-bet and GATA-3 control alternative pathways of T-cell differentiation through a shared set of target genes. Proc Natl Acad Sci U S A *106*, 17876-17881.

Kagey, M. H., Newman, J. J., Bilodeau, S., Zhan, Y., Orlando, D. A., van Berkum, N. L., Ebmeier, C. C., Goossens, J., Rahl, P. B., Levine, S. S., Taatjes, D. J., Dekker, J., Young, R.A. (2010). Mediator and cohesin connect gene expression and chromatin architecture. Nature *467,* 430-435.

Landt, S.G., Marinov, G.K., Kundaje, A., Kheradpour, P., Pauli, F., Batzoglou, S., Bernstein, B.E., Bickel, P., Brown, J.B., Cayting, P., *et al.* (2012). ChIP-seq guidelines and practices of the ENCODE and modENCODE consortia. Genome Res *22*, 1813-1831.

Li, Q., Brown, J.B., Huang, H., Bickel, P.J. (2011). Measuring reproducibility of high-throughput experiments. Ann Appl Stat *5*, 1752-1779.

Liao, Y., Smyth, G.K., and Shi, W. (2014). featureCounts: an efficient general purpose program for assigning sequence reads to genomic features. Bioinformatics *30,* 923-930.

Lin, C., Garrett, A.S., De Kumar, B., Smith, E.R., Gogol, M., Seidel, C., Krumlauf, R., and Shilatifard, A. (2011). Dynamic transcriptional events in embryonic stem cells mediated by the super elongation complex (SEC). Genes Dev *25*, 1486-1498.

Lin, C., Garruss, A.S., Luo, Z., Guo, F., and Shilatifard, A. (2013). The RNA Pol II elongation factor Ell3 marks enhancers in ES cells and primes future gene activation. Cell *152*, 144-156.

Lin, C., Smith, E.R., Takahashi, H., Lai, K.C., Martin-Brown, S., Florens, L., Washburn, M.P., Conaway, J.W., Conaway, R.C., and Shilatifard, A. (2010). AFF4, a component of the ELL/P-TEFb elongation complex and a shared subunit of MLL chimeras, can link transcription elongation to leukemia. Mol Cell *37*, 429-437.

Luo, Z., Lin, C., Guest, E., Garrett, A.S., Mohaghegh, N., Swanson, S., Marshall, S., Florens, L., Washburn, M.P., and Shilatifard, A. (2012). The super elongation complex family of RNA polymerase II elongation factors: gene target specificity and transcriptional output. Mol Cell Biol *32*, 2608-2617.

Mueller, D., Garcia-Cuellar, M.P., Bach, C., Buhl, S., Maethner, E., and Slany, R.K. (2009). Misguided transcriptional elongation causes mixed lineage leukemia. PLoS Biol *7*, e1000249.

Rahl, P.B., Lin, C.Y., Seila, A.C., Flynn, R.A., McCuine, S., Burge, C.B., Sharp, P.A., and Young, R.A. (2010). c-Myc regulates transcriptional pause release. Cell *141*, 432-445.

Shao, Z., Zhang, Y., Yuan, G. C., Orkin, S. H., Waxman, D. J. (2012). MAnorm: a robust model for quantitative comparison of ChIP-Seq data sets. Genome Biol. *3*, R16.

Shen, L., Shao, N., Liu, X., and Nestler, E. (2014). ngs.plot: Quick mining and visualization of next-generation sequencing data by integrating genomic databases. BMC Genomics *15*, 284.

Su, A.I., Wiltshire, T., Batalov, S., Lapp, H., Ching, K.A., Block, D., Zhang, J., Soden, R., Hayakawa, M., Kreiman, G., *et al.* (2004). A gene atlas of the mouse and human protein-encoding transcriptomes. Proc Natl Acad Sci U S A *101*, 6062-6067.

Zeitlinger J., Stark A., Kellis M, Hong JW, Nechaev S, Adelman K, Levine M, Young RA. (2007). RNA polymerase stalling at developmental control genes in the Drosophila melanogaster embryo. Nat Genet *39*, 1512-1516.

Zhang, Y., Liu, T., Meyer, C.A., Eeckhoute, J., Johnson, D.S., Bernstein, B.E., Nusbaum, C., Myers, R.M., Brown, M., Li, W., and Liu, X.S. (2008). Model-based analysis of ChIP-Seq (MACS). Genome Biol *9*, R137.
