# Supplementary material for: T-bet Activates Th1 Genes through Mediator and the Super Elongation Complex
Source: Cell Rep. 2016 Jun 9;15(12):2756–70. doi: 10.1016/j.celrep.2016.05.054 (PMC4920892; doi:10.1016/j.celrep.2016.05.054)
Supplement: Document S2. Article plus Supplemental Information [file mmc9.pdf]

Cell Reports

Article

# T-bet Activates Th1 Genes through Mediator and the Super Elongation Complex

## Graphical Abstract

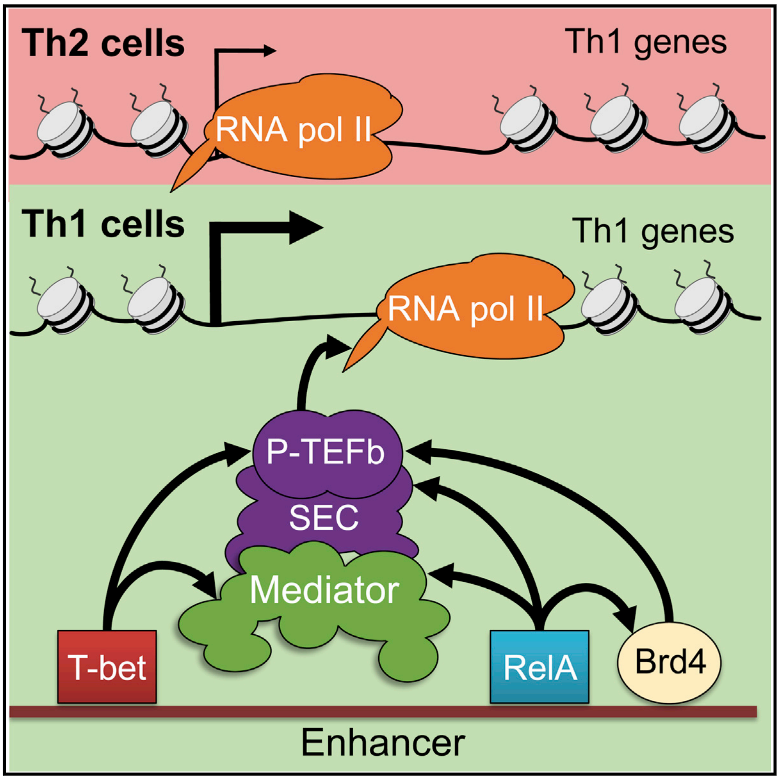


## Highlights

- Th1 genes and Th2 genes are associated with RNA pol II in the alternative lineage
- T-bet acts to recruit Mediator and the SEC to activate Th1 genes and eRNAs
- T-bet and NF-κB-dependent P-TEFb recruitment pathways converge at enhancers
- P-TEFb inhibition silences T-bet target genes and abrogates uveitis in vivo

## Authors

Arnulf Hertweck, Catherine M. Evans, Malihe Eskandarpour, ...,
Virginia L. Calder, Graham M. Lord, Richard G. Jenner

## Correspondence

graham.lord@kcl.ac.uk (G.M.L.),
r.jenner@ucl.ac.uk (R.G.J.)

## In Brief

Lineage-specifying transcription factors control T helper cell fate choice, but the mechanisms underlying this are unclear. Hertweck et al. reveal that Th1 genes undergo transcriptional initiation in the alternative Th2 lineage and that the Th1 master regulator T-bet functions to recruit Mediator and the super-elongation complex to activate transcriptional elongation.

## Accession Numbers

GSE62486

Hertweck et al., 2016, Cell Reports *15*, 2756–2770
June 21, 2016 © 2016 The Author(s).
http://dx.doi.org/10.1016/j.celrep.2016.05.054

OPEN ACCESS
CellPress

Cell Reports
Article

# T-bet Activates Th1 Genes through Mediator and the Super Elongation Complex

Arnulf Hertweck,[1,2] Catherine M. Evans,[1] Malihe Eskandarpour,[3] Jonathan C.H. Lau,[1] Kristine Oleinika,[1] Ian Jackson,[2] Audrey Kelly,[4] John Ambrose,[1] Peter Adamson,[3] David J. Cousins,[4,5] Paul Lavender,[4] Virginia L. Calder,[3] Graham M. Lord,[2,6,*] and Richard G. Jenner[1,6,*]
[1]UCL Cancer Institute, University College London, 72 Huntley Street, W1T 4JF London, UK
[2]Department of Experimental Immunobiology and NIHR Comprehensive Biomedical Research Centre, Guy's and St. Thomas' Hospital and King's College London, SE1 9RT London, UK
[3]UCL Institute of Ophthalmology, University College London, EC1V 9EL London, UK
[4]Department of Asthma, Allergy, and Respiratory Science, King's College London, SE1 9RT London, UK
[5]Leicester Institute for Lung Health and Department of Infection, Immunity, and Inflammation, NIHR Leicester Respiratory Biomedical Research Unit, University of Leicester, LE3 9QP Leicester, UK
[6]Co-senior author
*Correspondence: graham.lord@kcl.ac.uk (G.M.L.), r.jenner@ucl.ac.uk (R.G.J.)
http://dx.doi.org/10.1016/j.celrep.2016.05.054

## SUMMARY

**The transcription factor T-bet directs Th1 cell differentiation, but the molecular mechanisms that underlie this lineage-specific gene regulation are not completely understood. Here, we show that T-bet acts through enhancers to allow the recruitment of Mediator and P-TEFb in the form of the super elongation complex (SEC). Th1 genes are occupied by H3K4me3 and RNA polymerase II in Th2 cells, while T-bet-mediated recruitment of P-TEFb in Th1 cells activates transcriptional elongation. P-TEFb is recruited to both genes and enhancers, where it activates enhancer RNA transcription. P-TEFb inhibition and Mediator and SEC knockdown selectively block activation of T-bet target genes, and P-TEFb inhibition abrogates Th1-associated experimental autoimmune uveitis. T-bet activity is independent of changes in NF-κB RelA and Brd4 binding, with T-bet- and NF-κB-mediated pathways instead converging to allow P-TEFb recruitment. These data provide insight into the mechanism through which lineage-specifying factors promote differentiation of alternative T cell fates.**

## INTRODUCTION

The differentiation of T helper cells into specialized effector lineages is a powerful model for understanding how master regulator transcription factors establish cell identity. Upon encounter with antigen, naive T cells can differentiate into one of several effector lineages (Zhu et al., 2010). The paradigm for the study of the T helper cell fate choice is the differentiation into either Th1 or Th2 lineages. Th1 cells activate cell-mediated immunity, essential to combat viral and intracellular bacterial infection, while Th2 cells orchestrate a humoral response to parasites. Inappropriate Th1 and Th2 responses are associated with autoimmunity and allergy, respectively. A degree of plasticity also exists between the different lineages and this may allow the immune response to be tuned as environmental cues vary (Murphy and Stockinger, 2010; O'Shea and Paul, 2010).

CD4 T cell fate choice is governed by a set of lineage-specifying transcription factors, which are activated differentially depending on the cytokine environment (Zhu et al., 2010). T-bet is necessary and sufficient for Th1 cell differentiation (Lazarevic et al., 2013; Szabo et al., 2000). How T-bet promotes Th1 differentiation has primarily been determined through the study of *Ifng.* T-bet activates *Ifng* by binding at the gene itself and to multiple enhancer elements spanning a 146 kb region up and downstream (Balasubramani et al., 2010; Hatton et al., 2006; Schoenborn et al., 2007; Shnyreva et al., 2004). T-bet has been reported to be necessary for recruitment of the NF-κB family member RelA (Balasubramani et al., 2010), the Setd7 H3K4 methyltransferase complex, and the H3K27 demethylase Kdm6b (Jmjd3; Miller et al., 2008) to *Ifng*, but the extent to which T-bet utilizes these mechanisms across the genome is unknown. T-bet also recruits p300 to the *Ifng* locus, but across the genome, only 17% of p300 binding sites in Th1 cells are dependent on T-bet, suggesting that it has a limited role in establishing the binding pattern of this co-factor (Vahedi et al., 2012).

Analysis of T-bet binding across the mouse and human genomes has revealed hundreds of immune regulatory genes at which T-bet binds across extended *cis*-regulatory regions similar to that at *Ifng* (Kanhere et al., 2012; Nakayamada et al., 2011; Zhu et al., 2012). Although T-bet binds to the promoters of thousands of genes, it only functions at the subset of genes associated with these extended regulatory regions (Kanhere et al., 2012).

Similar regions of dense transcription factor binding have been identified in a number of cell types and termed super-enhancers (Brown et al., 2014; Chapuy et al., 2013; Di Micco et al., 2014; Hnisz et al., 2013; Lovén et al., 2013; Whyte et al., 2013), transcription initiation platforms (Koch et al., 2011), or stretch enhancers (Parker et al., 2013). Super-enhancers are defined computationally as genomic regions that display unusually high

 © 2016 The Author(s).
This is an open access article under the CC BY license (http://creativecommons.org/licenses/by/4.0/).

levels of occupancy of a transcription factor or co-activator. Super-enhancers share similarities with locus control regions characterized by functional studies (Smith and Shilatifard, 2014), and genes associated with super-enhancers tend to be cell-type specific (Hnisz et al., 2013; Koch et al., 2011; Parker et al., 2013; Whyte et al., 2013). Thus, super-enhancer definition provides a useful tool for identifying a set of candidate regulatory loci important for the identity of the cell. As such, p300 binding has been used to identify super-enhancers and genes specific to Th1, Th2, and Th17 lineages (Vahedi et al., 2015).

In embryonic stem cells (ESCs), regions defined as super-enhancers are highly occupied by the co-activators Mediator, CBP, and p300, the BET protein Brd4, cohesin, and the Lsd1-NuRD complex (Hnisz et al., 2013; Whyte et al., 2013; Di Micco et al., 2014). The high levels of Brd4 binding at activated oncogenes in cancer cells (Chapuy et al., 2013; Lovén et al., 2013) and at proinflammatory genes in endothelial cells (Brown et al., 2014) and T cells (Peeters et al., 2015) renders them hyper-sensitive to transcriptional repression by BET-inhibitors, providing a potential therapeutic route for cancer and inflammatory diseases. However, the mechanisms by which T-bet functions at enhancers to activate Th1 gene expression are still unclear.

Using a combination of primary human T cells and mouse models, we report here that Th1 genes undergo transcriptional initiation in Th1 and Th2 cells and that T-bet activates Th1 genes through recruitment of Mediator and the super elongation complex, with Brd4 instead recruited in a parallel pathway dependent on NF-κB.

## RESULTS

### Transcriptional Initiation at Th1 and Th2 Genes in the Opposing Cell Lineage

We hypothesized that profiling total RNA pol II occupancy by chromatin immunoprecipitation (ChIP) sequencing (ChIP-seq) in human Th1 and Th2 cells would provide insight into how T-bet functions to regulate gene expression. We first defined Th1 and Th2 genes as those expressed differentially across multiple samples, as done previously (see Wei et al., 2011; Hawkins et al., 2013; Stubbington et al., 2015). As expected, plotting ChIP-seq read density across Th1 genes in Th1 cells revealed occupancy of RNA pol II at transcription start sites (TSS), which increased upon restimulation (Figures 1A, 1E, and S1A–S1C; Table S1). RNA pol II was also present at Th1 genes in Th2 cells, albeit at a reduced level. Similarly, RNA pol II occupied Th2 genes in both Th1 and Th2 cells (Figures 1A, 1E, and S1A–S1C). Comparison of RNA pol II levels across Th1 genes between restimulated Th1 and Th2 cells revealed an increasing difference across the gene body, suggesting greater elongation efficiency in Th1 cells (Figure 1B). A corresponding effect was observed for Th2 genes, with RNA pol II showing increased elongation efficiency in Th2 cells (Figure 1B).

We sought to confirm transcriptional initiation of Th1 and Th2 genes in both lineages by measuring the initiation marker histone H3 trimethylated at lysine 4 (H3K4me3) in cells polarized for extended periods of time (28 days; Figures 1C, 1E, S1D, and S1E; Table S1). As we found for RNA pol II, H3K4me3 was present at Th1 and Th2 genes in both cell lineages. This was also apparent using an independent previously defined set of human Th1 and Th2 genes (Hawkins et al., 2013) (Figure S1G) and in murine Th1 and Th2 cells using our own and previously defined Th1 and Th2 gene sets (Wei et al., 2011; Stubbington et al., 2015) and our own and previously acquired H3K4me3 ChIP-seq data (Wei et al., 2009) (Figures S1H–S1J). To test whether this phenomenon also occurred in cells polarized in vivo, we performed ChIP-seq for H3K4me3 in human CCR5+ Th1 memory cells. This revealed the presence of H3K4me3 at both Th1 and Th2 genes in these cells, showing that this finding was not an artifact of in vitro polarization (Figures 1D, S1F, and S1G). Taken together with our RNA pol II binding data, these results show that transcription is initiated at Th1 and Th2 genes in both Th1 and Th2 cells and suggests that differential expression is primarily a function of differences in transcriptional elongation.

### Recruitment of P-TEFb to Th1 Genes and Super-Enhancers

We have previously shown that T-bet regulates gene expression by binding to multiple distal sites at extended *cis*-regulatory regions (Kanhere et al., 2012). We sought to determine whether these regions also fall into the definition of super-enhancers. Analysis of replicate T-bet ChIP-seq experiments using the ROSE algorithm (Hnisz et al., 2013) identified 374 super-enhancers in human Th1 cells (Figures S2A and S2B; Table S2). As expected, T-bet super-enhancers were associated with high levels of H3K27 acetylation (Figure S2C), CD4+ T cell-specific expression (Figure S2D), and functions related to the immune response (Figure S2E).

We next considered how T-bet function may be linked to lineage-specific transcriptional elongation and hypothesized that differential recruitment of the elongation factor P-TEFb may be involved. ChIP-seq for P-TEFb revealed increased recruitment in activated Th1 cells to genes with T-bet super-enhancers (Figures 2A, 2B, and S2F). In contrast, RNA pol II occupancy was similar at genes associated with super-enhancers and typical enhancers and was also comparable between Th1 and Th2 cells. Indeed, genes with super-enhancers exhibited significantly higher P-TEFb occupancy at their start sites compared to other active genes, even when considering the level of RNA pol II occupancy ($p < 2 \times 10^{-16}$ [K-S (Kolmogorov-Smirnov) test]; Figure S2G).

In addition to binding at genes, we unexpectedly found that P-TEFb also bound extensively to intergenic sites, with 35% of sites located outside of genes (Figures 2B and 2C), double that of RNA pol II (17%) and also greater than the active enhancer mark H3K27ac (24%). In intergenic regions, P-TEFb was localized to T-bet binding sites associated with H3K27ac (Figure S2H), with P-TEFb exhibiting particularly extensive binding at T-bet super-enhancers (Figures 2B and 2D). Indeed, 75% of genes associated with P-TEFb at the TSS and at an intergenic site were T-bet targets, compared with only 6% of genes where P-TEFb occupied the TSS only. The binding of P-TEFb to intergenic sites appeared to be functionally important; genes occupied by P-TEFb at the TSS and an intergenic site were overexpressed in Th1 cells relative to Th2 cells (Figure 2E). Furthermore, genes occupied by P-TEFb at the TSS and an intergenic site were enriched for functions related to the immune response, whereas, in comparison, genes only occupied by P-TEFb at the TSS had functions in cell metabolism and translation (Figure 2F).

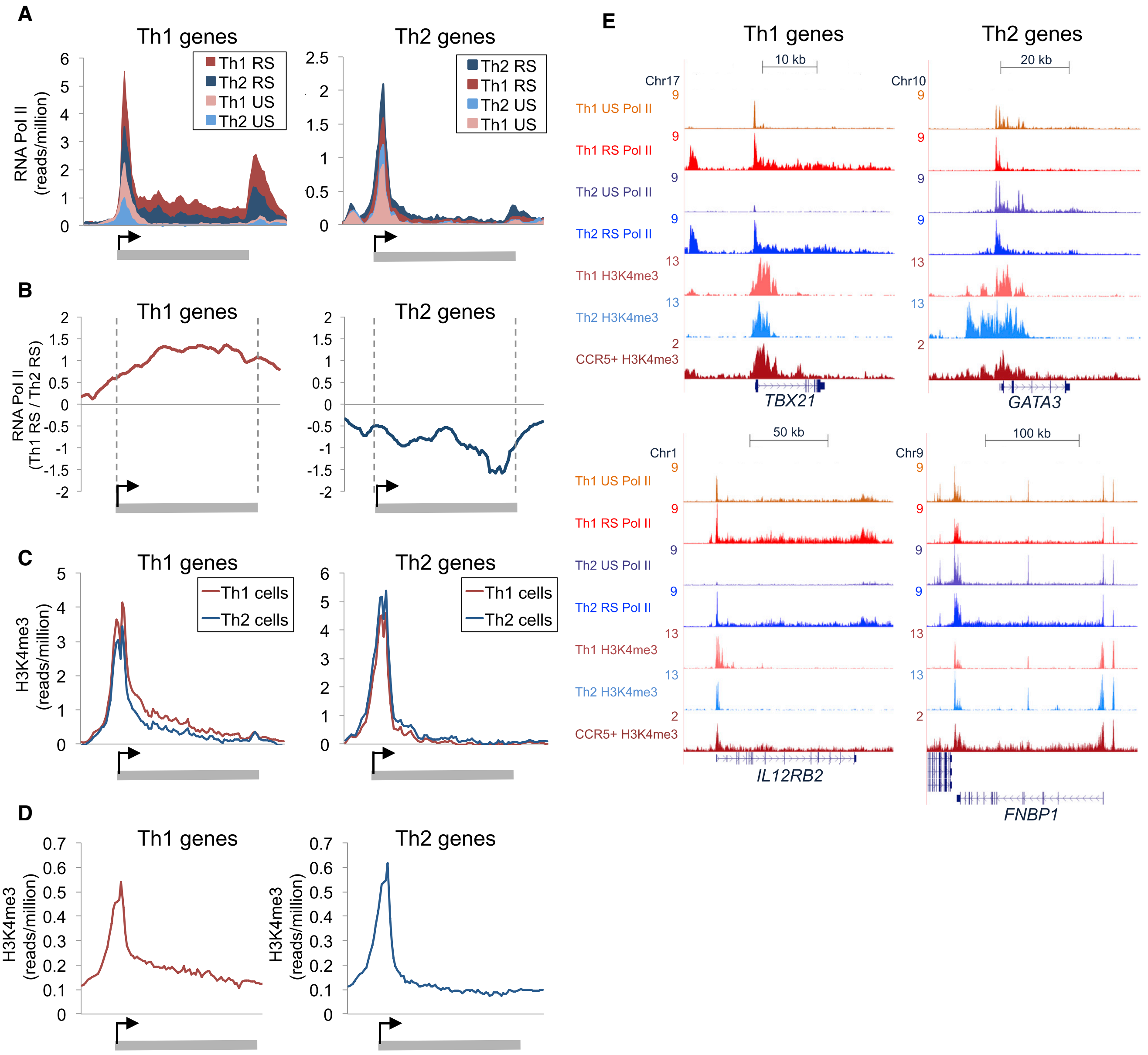


**Figure 1. Transcriptional Initiation at Th1 and Th2 Genes in Both Lineages**

(A) Average number of ChIP-seq reads for RNA pol II (reads/million) across Th1 genes (left) and Th2 genes (right) in unstimulated (US) or restimulated (RS) human Th1 and Th2 cells.

(B) Log2 ratio of RNA pol II across Th1 genes (left) and Th2 genes (right) between RS Th1 and Th2 cells. The difference in RNA pol II levels increases across the genes.

(C) As in (A), except for H3K3me3 in Th1 and Th2 cells polarized for an extended period of time.

(D) As in (A), except for H3K4me3 in CCR5+ Th1 memory cells.

(E) ChIP-seq binding profiles for RNA pol II and H3K4me3 at example Th1 genes and Th2 genes in in vitro polarized Th1 and Th2 cells and in CCR5+ Th1 memory cells. See also Figure S1.

These data suggest that Th1 genes are regulated in a distinct manner involving P-TEFb recruitment to enhancers.

## Th1 Genes Are Hyper-Sensitive to Inhibition of Elongation

The high-level of P-TEFb binding at Th1 and Th2 genes argues that the switch from transcriptional initiation to elongation is a particularly critical control point in the expression of these genes. To test this, we measured gene expression in in vitro differentiated mouse Th1 and Th2 cells in the presence of the P-TEFb inhibitor Flavopiridol or the BET inhibitor JQ1, which inhibits P-TEFb recruitment (Brown et al., 2014; Chapuy et al., 2013; Di Micco et al., 2014; Lovén et al., 2013). We found that Th1 gene expression was significantly reduced by Flavopiridol and JQ1

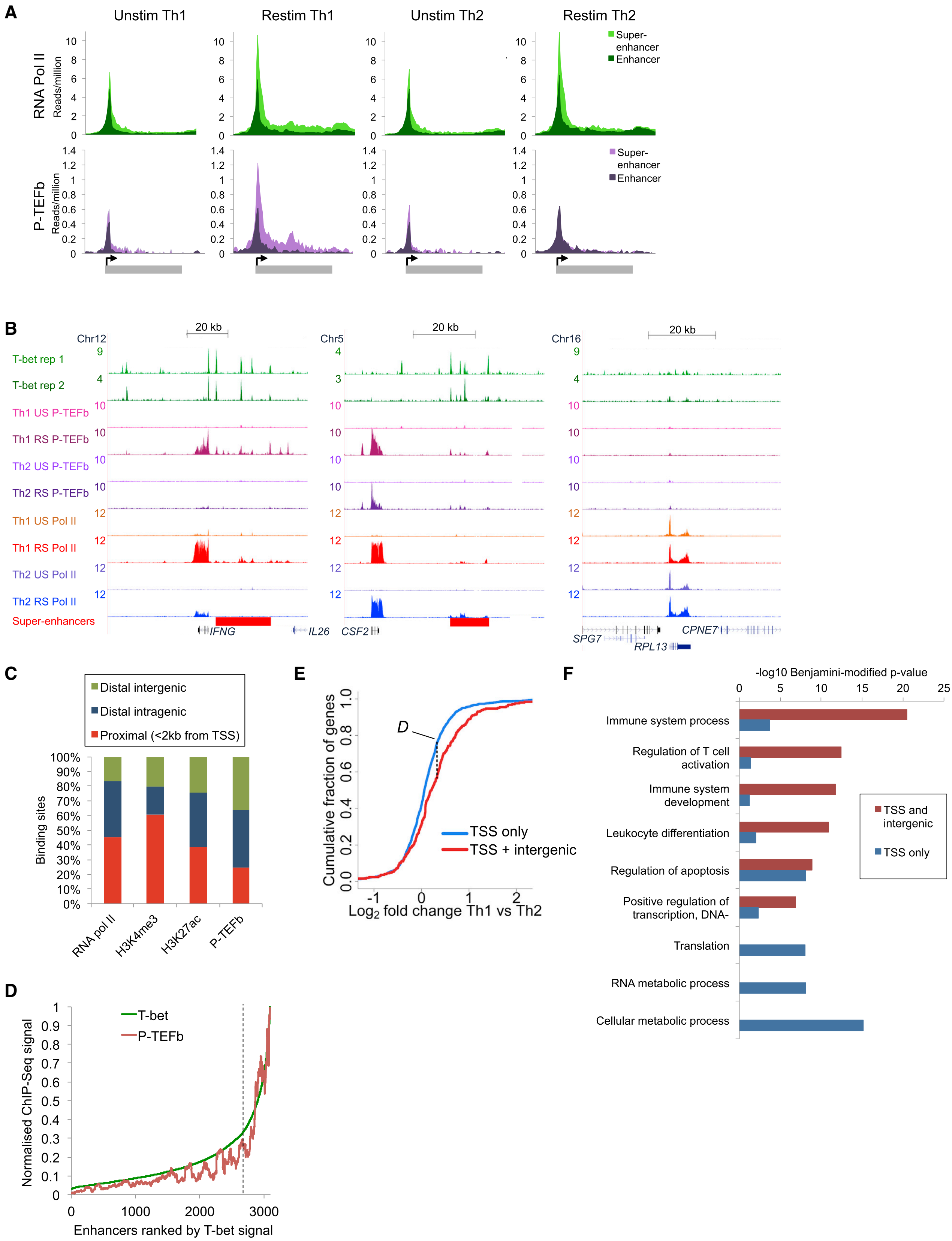


*(legend on next page)*

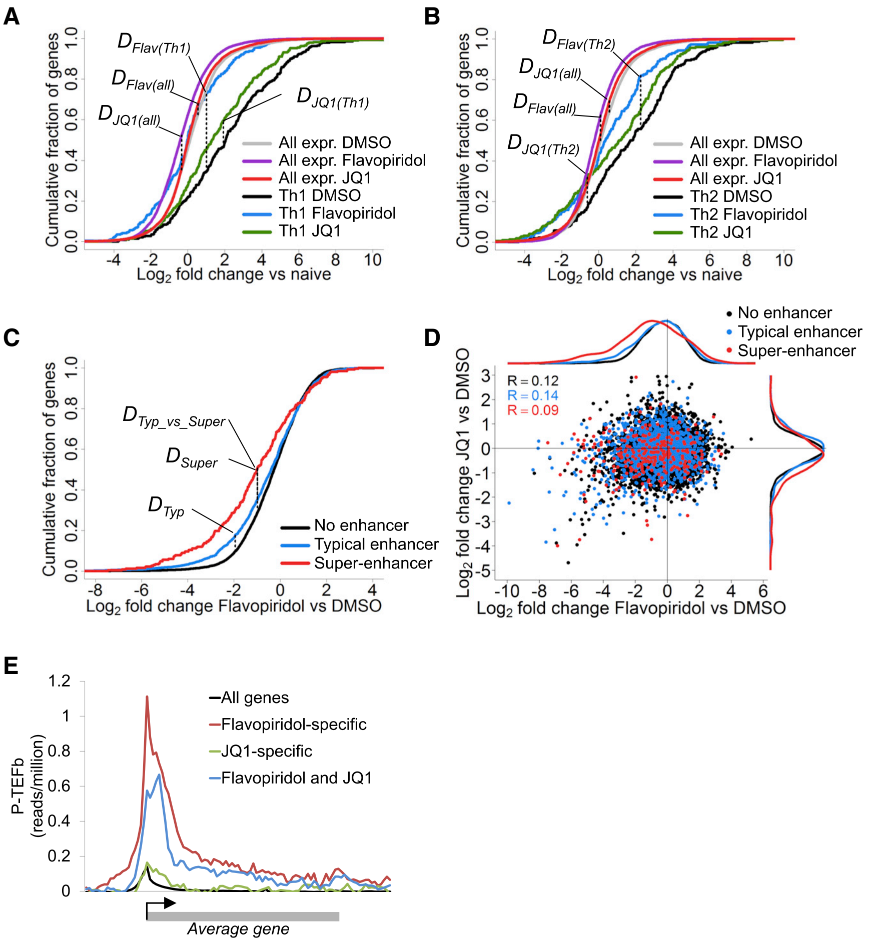


**Figure 3. Th1 Genes Are Hyper-Sensitive to Inhibition of Transcriptional Elongation**

(A) Cumulative distribution frequency of gene expression changes in Th1 cells treated with DMSO, Flavopiridol (10 μM), or JQ1 (500 nM) relative to naive T cells. The genes are divided into Th1 genes (n = 291) or all expressed genes (n = 8,095). $D_{Flav(all)}$ = 0.17, p < 2.2 × $10^{-16}$; $D_{Flav(Th1)}$ = 0.39, p < 2.2 × $10^{-16}$; $D_{JQ1(all)}$ = 0.053, p = 1.94 × $10^{-10}$; and $D_{JQ1(Th1)}$ = 0.13, p = 0.011 (K-S test).

(B) As in (A), except for Th2 genes (n = 228) or all expressed genes (n = 8,095) in Th2 cells. $D_{Flav(all)}$ = 0.13, p < 2.2 × $10^{-16}$; $D_{Flav(Th2)}$ = 0.26, p = 2.39 × $10^{-7}$; $D_{JQ1(all)}$ = 0.054, p = 1.02 × $10^{-10}$; and $D_{JQ1(Th2)}$ = 0.155, p = 0.92 × $10^{-3}$ (K-S test).

(C) Expression changes of genes in Th1 cells in response to Flavopiridol versus DMSO control. Genes with typical T-bet enhancers (blue, n = 1,561), T-bet super-enhancers (red, n = 270), or neither (black, n = 6,264) are shown. $D_{Typ}$ = 0.081, p = 1.21 × $10^{-7}$; $D_{Super}$ = 0.22, p = 9.54 × $10^{-12}$; and $D_{Typ\_vs\_Super}$ = 0.16, p = 1.55 × $10^{-5}$ (K-S test).

(D) Scatterplot of changes in gene expression in Th1 cells in response to Flavopiridol versus changes in response to JQ1. The genes are divided as in (C).

(E) Average ChIP-seq density for P-TEFb in human Th1 cells across all genes, genes repressed by both Flavopiridol and JQ1 (n = 232), and genes only repressed by either Flavopiridol (n = 109) or JQ1 (n = 128).

See also Figure S3.

(Figures 3A and S3A). The effect of Flavopiridol was much greater than that of JQ1, completely reversing the induction of Th1 genes. In contrast, other genes exhibiting the same expression levels were not substantially affected by either drug (Figures 3A and S3B). Similar results were observed for Th2 genes (Figure 3B). Thus, consistent with their high-levels of P-TEFb binding, Th1 and Th2 genes are hyper-sensitive to inhibition of transcriptional elongation compared to other expressed genes.

We next assessed whether the sensitivity of Th1 genes to elongation inhibition was related to super-enhancer function. We first identified the set of T-bet super-enhancers in mouse Th1 cells using replicate T-bet ChIP-seq data (Figure S3C; Table S2). Then, dividing genes into those associated with super-enhancers, typical enhancers, or neither, revealed that Flavopiridol blocked expression of genes associated with super-enhancers, but had little effect on those associated with typical enhancers (Figure 3C). Thus, super-enhancers show a specific requirement for P-TEFb to activate gene transcription.

Although JQ1 repressed a number of key Th1 genes (Figure S3E), the effect of JQ1 on Th1 gene expression was less striking than that of Flavopiridol (Figures 3D and S3E), suggesting that Brd4 does not play such a major role at these genes. Indeed, JQ1

**Figure 2. Extensive P-TEFb Binding at Super-Enhancers and Associated Genes**

(A) Average number of ChIP-seq reads for RNA pol II and P-TEFb in unstimulated (US) or restimulated (RS) human Th1 and Th2 cells. All genes are bound by RNA pol II in at least one condition and divided into those associated with a super-enhancer (n = 231) or a typical enhancer (n = 1,307).

(B) T-bet, P-TEFb, and RNA pol II binding at *IFNG*, *CSF2* (associated with T-bet super-enhancers), and the housekeeping gene *RPL13*.

(C) Percentage of sites for RNA pol II, H3K4me3, H3K27ac, and P-TEFb in Th1 cells that are proximal (<2 kb from TSS), distal intragenic, or distal intergenic.

(D) Distribution of T-bet and P-TEFb ChIP-seq signals across 3,191 T-bet Th1 enhancers, ranked according to T-bet signal. The enhancers classified as T-bet super-enhancers are to the right of the vertical dashed line. The ChIP signals are shown as moving averages (window size of 100 bp).

(E) Cumulative distribution frequency of gene expression in Th1 cells relative to Th2 cells for genes occupied by P-TEFb at the gene TSS only (blue, n = 970) or at the TSS and an intergenic site (red, n = 245) (*D* = 0.19, p = 7.47 × $10^{-7}$ [K-S test]).

(F) Significance of the enrichment of biological process gene ontology categories in the set of genes occupied by P-TEFb at the gene TSS only (blue) or at the gene TSS and an intergenic site (red).

See also Figure S2.

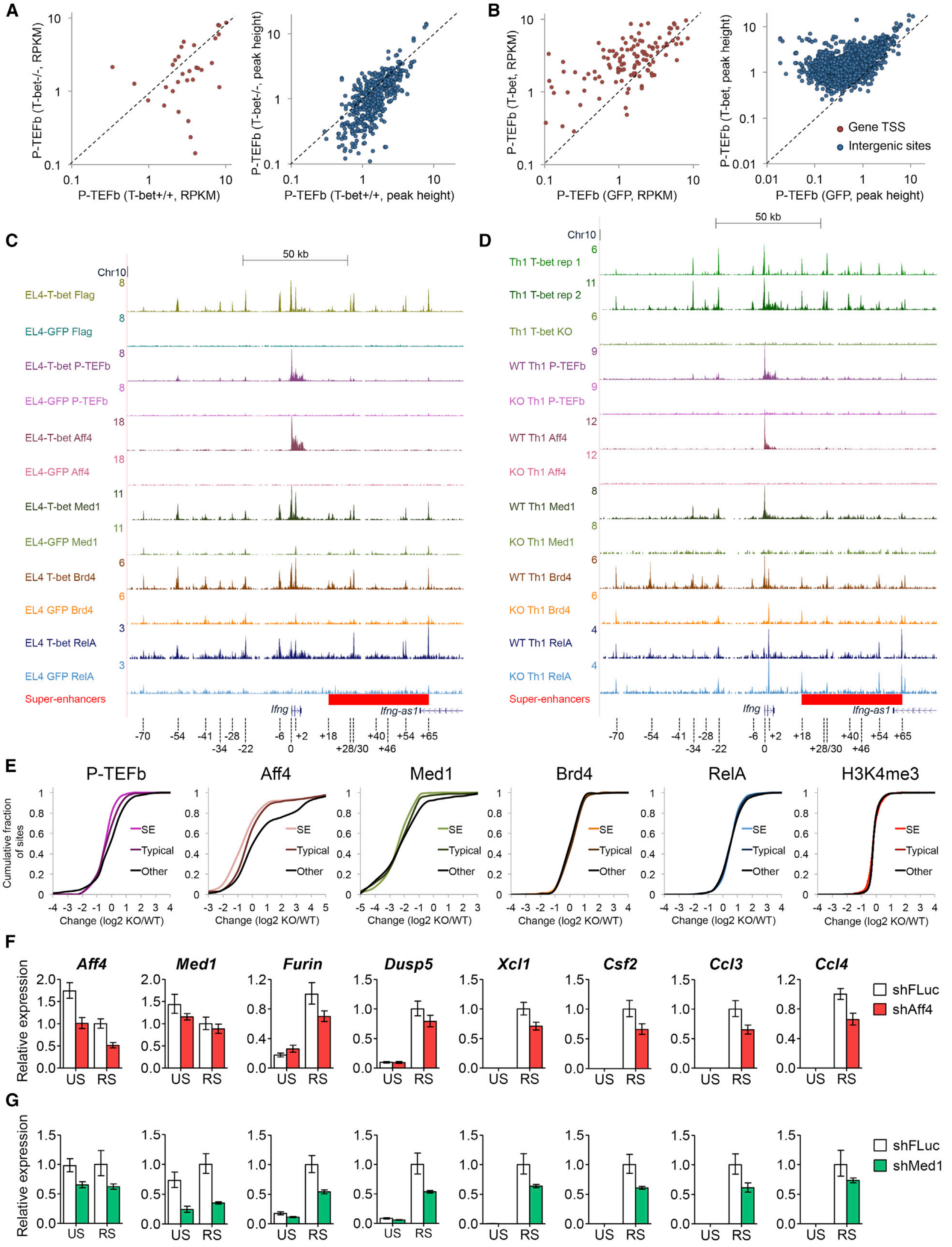


(legend on next page)

and Flavopiridol had markedly different effects in Th1 cells, with a Pearson correlation co-efficient of only 0.12 (Figure 3D). This was also apparent with lower concentrations of each drug, indicating that this was not a reflection of differential off-target effects (Figure S3D). Flavopiridol was more specific for P-TEFb target genes than JQ1, as demonstrated by higher levels of P-TEFb binding at genes specifically repressed by Flavopiridol (Figures 3E and S3E; Table S3). These data suggest that Brd4 plays a relatively minor role in the recruitment of P-TEFb to T-bet target genes.

### T-bet Functions to Recruit P-TEFb to Genes and Enhancers in Activated Th1 Cells

Given that P-TEFb bound with T-bet to super-enhancers and associated genes, we next asked whether T-bet functioned in the recruitment of P-TEFb. To test this, we first performed ChIP-seq for P-TEFb in restimulated CD4+ T cells purified from wild-type (WT) and T-bet$^{-/-}$ mice cultured under Th1 conditions. We found that in the absence of T-bet, P-TEFb showed reduced binding to super-enhancers and to their associated genes (Figure 4A). Thus, T-bet is necessary for the high levels of P-TEFb recruitment observed at Th1 genes and enhancers.

To determine if T-bet was sufficient to recruit P-TEFb, we employed two murine EL4 cell lines, one that stably expresses T-bet and GFP and a control line expressing GFP alone (Kanhere et al., 2012). ChIP-seq for P-TEFb in both cell lines showed that P-TEFb occupancy increased at super-enhancers and their associated genes when T-bet was present (Figure 4B). We could also detect an interaction between T-bet and P-TEFb by co-immunoprecipitation (Figures S4A–S4C), consistent with a role for T-bet in P-TEFb recruitment. We conclude that T-bet functions to allow recruitment of P-TEFb to super-enhancers and associated genes in activated Th1 cells.

### T-bet Is Necessary for Recruitment of Mediator and the Super Elongation Complex

We next explored how T-bet functioned in the recruitment of P-TEFb to super-enhancers and their associated genes. P-TEFb can be recruited to genes through NF-κB (Barboric et al., 2001), the BET domain protein Brd4 (Jang et al., 2005; Yang et al., 2005), and by Mediator (Donner et al., 2010; Wang et al., 2013), which recruits P-TEFb as part of the super elongation complex (SEC) (Takahashi et al., 2011).

To test whether these mechanisms were in operation at T-bet super-enhancers, we measured the effect of T-bet expression in activated EL4 cells on the binding of RelA, Brd4, the Mediator subunit Med1, and the core SEC component Aff4. First focusing on *Ifng,* we found that T-bet induced binding of each protein to the promoter and to multiple distal regulatory elements that surround the gene (Figure 4C). To quantify the effect of T-bet on the binding of each factor across the genome, we measured the change in binding upon T-bet expression at all super-enhancers and associated genes compared to the change in binding at other sites across the genome and plotted the cumulative distribution frequencies (Figures S4D and S4E; Table S4). This revealed significant increases in P-TEFb, Aff4, Med1, and Brd4 occupancy at super-enhancers and their associated genes in EL4 cells when T-bet was present ($p < 0.05$, Mann-Whitney U test). Thus, we conclude that T-bet is sufficient for the recruitment of P-TEFb, Aff4, Med1, and Brd4 in activated cells.

We then asked whether T-bet was necessary for the recruitment of Aff4, Med1, Brd4, and RelA by measuring changes in their binding between restimulated WT and T-bet$^{-/-}$ cells cultured under Th1 conditions. We also measured levels of the transcriptional initiation marker H3K4me3, to monitor this stage of gene activation. Comparing super-enhancers and their associated genes with other sites, we noted a marked deficiency in P-TEFb, Aff4, and Med1 recruitment in T-bet$^{-/-}$ cells ($p < 0.05$, Mann-Whitney U test; Figures 4D, 4E, and S4F; Table S5). In contrast, recruitment of Brd4 and RelA were unaffected by T-bet deletion ($p > 0.05$; Figures 4E and S4F). Thus, T-bet is necessary for recruitment of Mediator and the SEC, but not for Brd4 and RelA binding. Consistent with a lack of a direct role for T-bet in RelA recruitment, a mutant form of T-bet (S508A), which does not interact with RelA (Hwang et al., 2005), had no effect on RelA binding at *Ifng* in EL4 cells (Figure S4G). H3K4me3 levels at super-enhancers and associated genes were also unaffected upon T-bet loss (Figure 4E), consistent with the presence of H3K4me3 at Th1 genes in Th2 cells (Figure 1) and confirming that T-bet does not act through super-enhancers to regulate this stage of gene activation.

Although T-bet deletion did not cause significant changes to Brd4 and RelA binding at super-enhancers compared to other sites, some binding events, such as the *Ifng* −54, −34 kb, and promoter sites, were dependent on T-bet (Figure 4D). To assess whether these T-bet-dependent sites were associated with P-TEFb recruitment, we identified the T-bet-dependent sites for each factor and measured the changes in P-TEFb binding at those locations (Figure S4H). We found that

**Figure 4. T-bet Is Necessary for Recruitment of Mediator, the SEC, and P-TEFb to Genes and Super-Enhancers**

(A) Scatterplot showing the read density (RPKM) for P-TEFb at super-enhancer-associated genes occupied by P-TEFb in primary mouse T-bet$^{+/+}$ versus T-bet$^{-/-}$ Th1 cells (left). A scatterplot showing the P-TEFb peak height (reads/million) at intergenic T-bet binding sites ($p < 10^{-9}$) in primary mouse T-bet$^{+/+}$ versus T-bet$^{-/-}$ Th1 cells is on the right.

(B) As in (A), except for activated EL4 cells stably expressing GFP (x axis) versus cells stably expressing T-bet and GFP (y axis).

(C) ChIP-seq binding profiles for T-bet, P-TEFb, RelA, Brd4, Med1, and Aff4 in EL4 cells stably expressing GFP alone or EL4 cells stably expressing FLAG-T-bet and GFP, with both cell lines restimulated with PMA and ionomycin. The positions of transcription factor binding sites relative to the *Ifng* TSS are marked (to the nearest kb).

(D) As in (C), except for WT and T-bet$^{-/-}$ Th1 cells restimulated with PMA and ionomycin.

(E) Cumulative distribution frequency of the change in P-TEFb, Aff4, Med1, Brd4, RelA, and H3K4me3 occupancy between T-bet$^{-/-}$ and T-bet$^{+/+}$ cells at super-enhancers and associated genes (SE), at typical enhancers and associated genes (Typical), and at other sites (Other).

(F) Expression of *Aff4* and *Med1* and the T-bet target genes *Furin*, *Dusp5*, *Xcl1*, *Csf2*, *Ccl3*, and *Ccl4* relative to *Hprt* (mean and SD, n = 2 biological replicates) in unstimulated and restimulated Th1 cells transduced with retroviruses encoding shRNAs against luciferase (white) or Aff4 (red).

(G) As in (F), except for shRNAs to luciferase (white) or Med1 (green) (mean and SD, n = 3 technical replicates). A replicate experiment with combined shRNA knock down of Med1 and Med17 is shown in Figure S4.

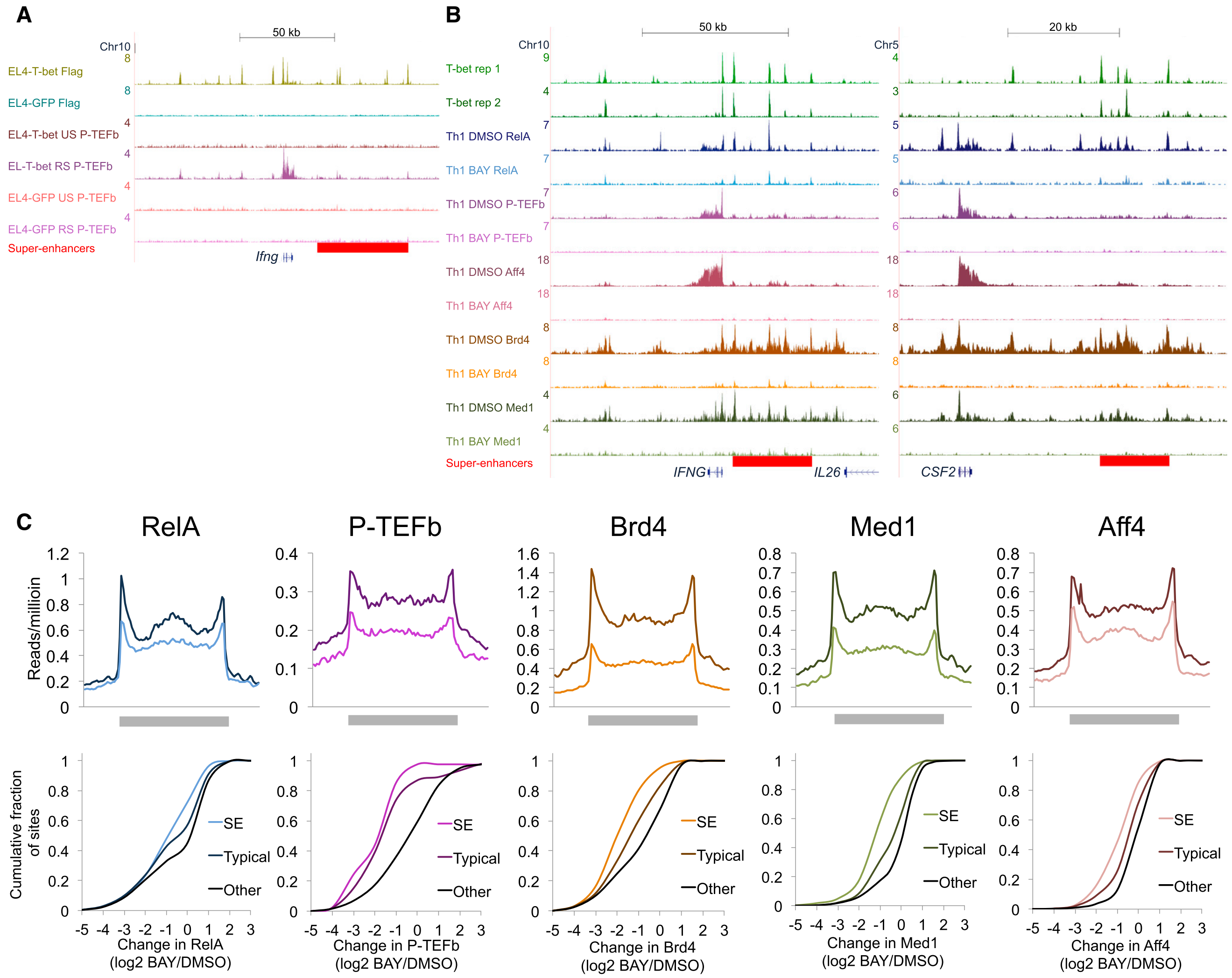


**Figure 5. NF-κB Is Necessary for P-TEFb Recruitment to T-bet Target Genes and Super-Enhancers**

(A) ChIP-seq binding profiles for FLAG-T-bet and P-TEFb at the *Ifng* locus in EL4-GFP and EL4-T-bet cells with and without restimulation.

(B) ChIP-seq binding profiles for RelA, P-TEFb, Brd4, Med1, and Aff4 at the *IFNG* locus in human Th1 cells with and without treatment with BAY 11-7082 (20 μM).

(C) Average number of ChIP-seq reads for RelA, P-TEFb, Brd4, Med1, and Aff4 at T-bet super-enhancers in human Th1 cells with and without treatment with BAY 11-7082 (20 μM) (top). Cumulative distribution frequency of the change in transcriptional regulator binding ($log_2$ BAY 11-7082 versus DMSO) for sites at super-enhancers and associated genes (SE), typical enhancers and associated genes, and at other sites bound by each factor (bottom).

See also Figure S5.

only T-bet-dependent recruitment of Med1 and Aff4 were associated with changes in P-TEFb occupancy. Thus, changes in Mediator and SEC binding, but not changes in Brd4 and RelA, are associated with T-bet-dependent P-TEFb recruitment.

To confirm whether Mediator and the SEC were important for the activation of T-bet target genes, we knocked down Med1 and Aff4 in mouse Th1 cells with small hairpin (sh)RNAs. We found that the expression of T-bet target genes such as *Ifng*, *Furin*, *Xcl1*, *Csf2, Ccl3*, and *Ccl4* were downregulated compared to the housekeeping gene *Hprt* (Figures 4F, 4G, S4I, and S4J). We conclude that T-bet operates through the Mediator-SEC pathway to allow recruitment of P-TEFb to super-enhancers and associated genes.

## RelA Is Necessary for Recruitment of P-TEFb, Mediator, Brd4, and the SEC to T-bet Target Genes

The ability of T-bet to recruit P-TEFb also requires cell restimulation (Figures 2A, 2B, 5A, and S5A), suggesting that additional factors were also necessary. In other cell types, RelA can recruit P-TEFb to genes directly (Barboric et al., 2001), through Brd4 (Brown et al., 2014; Huang et al., 2009; Sharma et al., 2007), or through Mediator (van Essen et al., 2009; Wienerroither et al., 2015). In Th1 cells, RelA binds to, and is necessary for, activation of *Ifng* (Balasubramani et al., 2010; Sica et al., 1997). However, the role of NF-κB in P-TEFb recruitment and Th1 gene activation across the genome is unknown. To address this, we first performed ChIP-seq for RelA in human Th1 cells and found that it

was associated with 75% of T-bet super-enhancers (Table S6). We then treated human Th1 cells with the IκB kinase inhibitor BAY 11-7082 and measured the change in RelA, P-TEFb, Brd4, Med1, and Aff4 recruitment by ChIP-seq. We found that IκB kinase inhibition significantly reduced the recruitment of all of these factors and that super-enhancers and their associated genes were particularly sensitive ($p < 10^{-7}$, Mann-Whitney U test; Figures 5B, 5C, and S5B). Thus, in addition to T-bet, NF-κB has a central role in recruitment of the transcriptional elongation machinery to T-bet target genes and super-enhancers across the genome. Furthermore, the lack of change in RelA and Brd4 binding upon T-bet deletion (Figure 4E) indicates that these pathways operate independently and converge at super-enhancers to allow P-TEFb recruitment.

## T-bet and P-TEFb Function at Super-Enhancers to Activate Enhancer RNA Transcription

Given that P-TEFb bound extensively at super-enhancers and this was associated with increased requirement for P-TEFb function (Figure 3C), we sought to determine whether P-TEFb plays a role at super-enhancers themselves. In other cell types, some enhancers produce enhancer (e)RNAs that contribute to enhancer function (Lam et al., 2014; Natoli and Andrau, 2012). We therefore hypothesized that P-TEFb functioned at T-bet super-enhancers in the production of eRNAs. To test this, we performed total and poly-A+ RNA-seq in human Th1 and Th2 cells and, to control for the increased size of super-enhancers, compared the numbers of sequence reads around T-bet binding sites within intergenic super-enhancers versus typical enhancers. We found that eRNA transcription was higher at T-bet binding sites within super-enhancers compared to those at typical enhancers (Figures 6A and S6A). Furthermore, eRNAs transcribed from T-bet super-enhancers tended to be Th1-specific (Figures 6B and S6B). At *IFNG*, eRNAs were transcribed in Th1 cells from the super-enhancer upstream of the gene (Figures 6C and S6F); enhancers downstream of *IFNG* exhibited lower levels of P-TEFb occupancy and eRNA production. *IFNG* eRNAs displayed features previously ascribed to eRNAs, being transcribed bidirectionally, unspliced, and non-poly-adenylated (Figures 6C and S6F). eRNAs could also clearly be observed at super-enhancers associated with other key lineage-specific genes (Figure S6H; Table S7).

These data suggested that T-bet functions at super-enhancers to induce eRNA transcription. To test this, we measured expression of *Ifng* eRNAs in Th1 and Th2 cells from T-bet$^{-/-}$ mice by quantitative (q)PCR. We found that the level of each eRNA tested was reduced in T-bet deficient cells, demonstrating that T-bet acts to induce eRNA transcription (Figure 6D). We next tested whether P-TEFb activity was required for *Ifng* eRNA production. Treatment of Th1 cells with JQ1 and Flavopiridol resulted in a marked reduction in eRNA levels, with Flavopiridol having the strongest effect (Figure 6E). Thus, P-TEFb functions at super-enhancers to activate eRNA transcription.

## Suppression of T-bet Function and Uveitis by P-TEFb Inhibition In Vivo

We sought to test the importance of P-TEFb in a Th1 response in vivo through use of a mouse experimental autoimmune uveitis (EAU) model, in which infiltration of interferon-γ-producing CD4+ T cells into the retina can be induced by immunization with interphotoreceptor retinoid-binding protein (IRBP) (Gardner et al., 2013). IRBP peptide was administered and, after 8 or 9 days, mice were treated with Flavopiridol (3 and 15 mg/kg) or JQ1 (3 and 30 mg/kg), and disease progression was scored by retinal fundoscopy and histology (Agarwal et al., 2012; Gardner et al., 2013). We found that both drugs significantly reduced disease severity. Immunized mice treated with carrier alone displayed severe EAU, characterized by disruption to retinal layers, diffuse retinal detachment, and folding, intense cellular infiltration and granulomatous lesions (histological score of 4; Figures 7A–7C and 7J). In contrast, mice treated with Flavopiridol and JQ1 showed reduced disease, with average histological scores between 1 and 2, minimal cell infiltration, and well-preserved photoreceptor layers (Figures 7D–7J, S7A, and S7B). We then sought to confirm that disease abrogation was reflected by a reduction in the expression of super-enhancer-associated Th1 genes. Flow cytometric analysis of CD4+ T cells sorted from the retina and lymph node revealed that expression of the super-enhancer-associated gene products Ifnγ, Tnf, Fasl, Il18r1, and Ctla4 were downregulated by Flavopiridol and JQ1 (Figures 7K, 7L, and S7C–S7G). We conclude that P-TEFb is required for Th1 gene expression in vivo and for Th1 cell-mediated immunopathology.

## DISCUSSION

How T-bet regulates Th1 gene expression across the genome has been unclear. We show here that Th1 and Th2 genes undergo transcriptional initiation in a lineage-independent manner and that T-bet acts through extended regulatory regions (super-enhancers) to allow recruitment of Mediator and P-TEFb in the form of the SEC to activate Th1 gene expression. T-bet is necessary for P-TEFb recruitment, not just to gene promoters, but to super-enhancers themselves, where it functions to activate eRNA transcription. P-TEFb inhibition specifically downregulates Th1 genes and alleviates pathology in a Th1 cell-dependent uveitis model. T-bet is not required for RelA or Brd4 recruitment to most sites at Th1 genes. Instead, T-bet and NF-κB-dependent pathways converge at super-enhancers to allow P-TEFb recruitment. These data thus provide insight into the mechanisms of T-bet function during Th1 lineage-specification in human and mouse.

Mediator is a large multi-subunit complex that integrates signals from multiple transcription factors to modulate transcription, chromatin modification, and looping between promoters and enhancers (Carlsten et al., 2013). It is particularly important for transcription of genes related to cell type specification, but has not previously been shown to play a role in transcription of such genes in T cells. Mediator promotes transcriptional elongation by recruiting P-TEFb via Med23 (Wang et al., 2013) and the CDK8 submodule (Donner et al., 2010) or as part of the SEC via Med26 (Takahashi et al., 2011). The SEC allows rapid gene induction in ESCs (Lin et al., 2013), thus it may perform a similar role in CD4+ T cells.

Unlike other systems (Brown et al., 2014; Chapuy et al., 2013; Di Micco et al., 2014; Lovén et al., 2013; Peeters et al., 2015), T-bet does not operate by inducing large-scale changes in

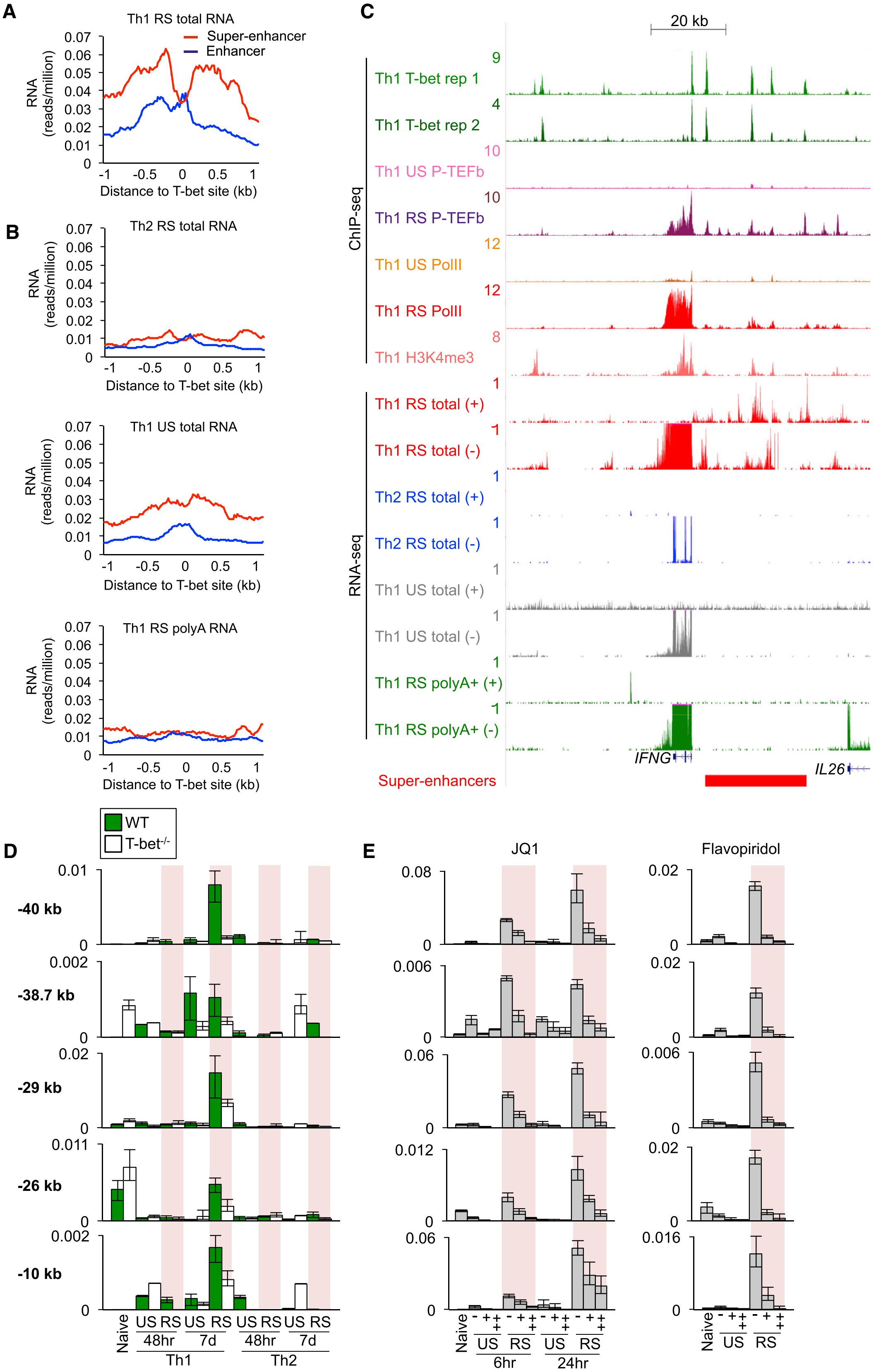


(legend on next page)

Brd4 binding. Instead, NF-κB activation is required for Brd4 recruitment in a parallel pathway. RelA has previously been shown to be required for *Ifng* expression downstream of the T cell receptor (TCR) (Balasubramani et al., 2010). We show here that RelA occupies the majority of T-bet bound super-enhancers and associated genes and, like T-bet, is necessary for recruitment of Mediator and the SEC, but is also necessary for Brd4 recruitment. However, there is no significant loss of RelA and Brd4 binding at T-bet super-enhancers and their associated genes compared to other sites in T-bet$^{-/-}$ cells. Thus, T-bet and RelA operate through separate, but co-dependent pathways, that converge at super-enhancers to allow recruitment of P-TEFb to T-bet target genes. This model is consistent with the ability of enhancer clusters to integrate inputs from multiple signaling pathways (Spitz and Furlong, 2012) and may constitute a control mechanism to ensure that Th1 gene activation only occurs when multiple immunological signals are received.

How lineage-specifying factors function to promote differentiation toward a defined lineage, but also maintain the functional plasticity observed between effector subtypes has been a key unresolved issue. Epigenetic profiling has previously suggested that the establishment of bivalent chromatin at key lineage-specific transcription factors may be important (Wei et al., 2009). Our results reveal more generally that Th1 and Th2 genes remain associated with RNA pol II and H3K4me3 in the opposing lineage, and that T-bet acts to allow recruitment of Mediator and the SEC to activate transcriptional elongation. This mechanism may contribute to the functional plasticity observed between T helper cell subtypes.

We identified extensive association of P-TEFb with enhancers. Brd4 (Brown et al., 2014; Chapuy et al., 2013; Di Micco et al., 2014; Hnisz et al., 2013; Lovén et al., 2013; Zhang et al., 2012) and the elongation factor Ell3 (Lin et al., 2013) have previously been identified at enhancers, but the extent of P-TEFb binding at these sites has not previously been observed. Our results support the notion that P-TEFb functions at super-enhancers to activate eRNA transcription. Th1 cell eRNAs share similarities with those observed previously in other cell types, being predominantly non-poly-adenylated and transcribed bidirectionally (Lam et al., 2014; Natoli and Andrau, 2012). Previous reports disagree on whether eRNA production in other cell types requires transcriptional elongation, but the loss of eRNAs upon Flavopiridol and JQ1 treatment demonstrates that P-TEFb is required for eRNA transcription in Th1 cells.

Our study into the mechanisms through which T-bet directs Th1 lineage-specification suggests potential therapeutic avenues. BET inhibitors have previously been used to repress transcriptional elongation in immune cells and protect against inflammation in a number of models (Bandukwala et al., 2012; Brown et al., 2014; Mele et al., 2013; Nicodeme et al., 2010; Peeters et al., 2015). Although our experiments reveal a requirement for BET domain proteins for Th1 gene expression, Flavopiridol was the more selective for Th1 genes, consistent with the invariance in Brd4 binding upon T-bet deletion. Thus, direct inhibition of P-TEFb or Mediator may represent a more efficacious and specific means of targeting Th1 genes for the treatment of inflammatory and autoimmune conditions.

## EXPERIMENTAL PROCEDURES

### Cells

Human and mouse naive T cells were isolated and cultured under Th1 and Th2 polarizing conditions for 13 days, as described (Kanhere et al., 2012). For H3K4me3 ChIP-seq, cells were differentiated for 28 days. Human CCR5+ Th1 memory T cells were purified as described (Messi et al., 2003). EL4-GFP and EL4-T-bet cells were described in Kanhere et al. (2012).

### Mice

C57BL/6 mice were purchased from Charles River Laboratories International. T-bet$^{-/-}$ mice were purchased from Taconic. B10.RIII mice were obtained from GlaxoSmithKline. Mice were housed at the KCL Biological Service Unit (BSU) or at the UCL Institute of Ophthalmology BSU. Animal experiments were performed in accordance with the UK Animals (Scientific Procedures) Act 1986 (Home Office License Numbers PPL: 70/6792, 70/7869 and 70/7265).

### ChIP-Seq

ChIP was performed as described (Kanhere et al., 2012), except H3K4me3 ChIP was performed on native chromatin. All antibodies used and data sets generated are listed in the Supplemental Information. Libraries were constructed using standard Illumina protocols and were sequenced with an Illumina GAIIx or HiSeq 2500. Reads were filtered to remove adapters using fastq-mcf and for quality using seqkt and aligned to hg19 or mm9 with Bowtie2 (default settings). Consistency between replicates was assessed by irreproducible discovery rate (IDR) analysis; in each case, Np/Nt was less than 2, the standard reproducibility threshold used by the ENCODE project. Significantly enriched regions were identified with MACS v1.4 using a p value threshold of $10^{-7}$ unless indicated. Super-enhancers were identified with the ROSE algorithm (Lovén et al., 2013; Whyte et al., 2013; Hnisz et al., 2013). The significance of changes in transcription factor binding upon T-bet expression was assessed with a Mann-Whitney U test.

### Microarray Analysis

Total RNA was labeled using the two-color Low Input Quick Amp Labeling Kit and hybridized to SurePrint G3 DNA microarrays (Agilent). Differentially expressed genes were identified by rank-sum test (pfp < 0.05). Murine Th1 and Th2 genes and genes repressed by Flavopiridol and/or JQ1 were identified by applying fold-change expression thresholds. The significance of differences in expression between gene sets was assessed with a K-S test.

**Figure 6. T-bet and P-TEFb-Dependent Production of Enhancer RNAs**

(A) Average total RNA-seq density (reads/million) in restimulated (RS) human Th1 cells at T-bet binding sites located within intergenic super-enhancers (n = 269) versus those within intergenic typical enhancers (n = 908).

(B) As in (A), except for Th2 cells (same scale as A) (top). As in (A), except for unstimulated Th1 cells (middle). As in (A), except for mRNA in RS Th1 cells (bottom).

(C) Total RNA and mRNA-seq data (reads/million) at *IFNG* showing production of non-poly-adenylated RNAs from the + (Watson) or − (Crick) strands in restimulated (RS) human Th1 cells. The read density at the *IFNG* gene extends beyond the maximum y axis value. The ChIP-seq binding profiles are shown above.

(D) qRT-PCR for eRNAs (relative to *Hprt*, mean and SD, and n = 3 technical replicates) in WT and T-bet$^{-/-}$ naive mouse T cells and unstimulated (US) or restimulated (RS) Th1 and Th2 cells polarized for 48 hr or 7 days. The eRNAs are labeled according to their position relative to the *Ifng* TSS.

(E) As in (D), except for WT cells treated with 50 (+) or 500 nM (++) JQ1 (left) or treated with 1 (+) or 10 μM (++) Flavopiridol for 6 hr (right).

See also Figure S6.

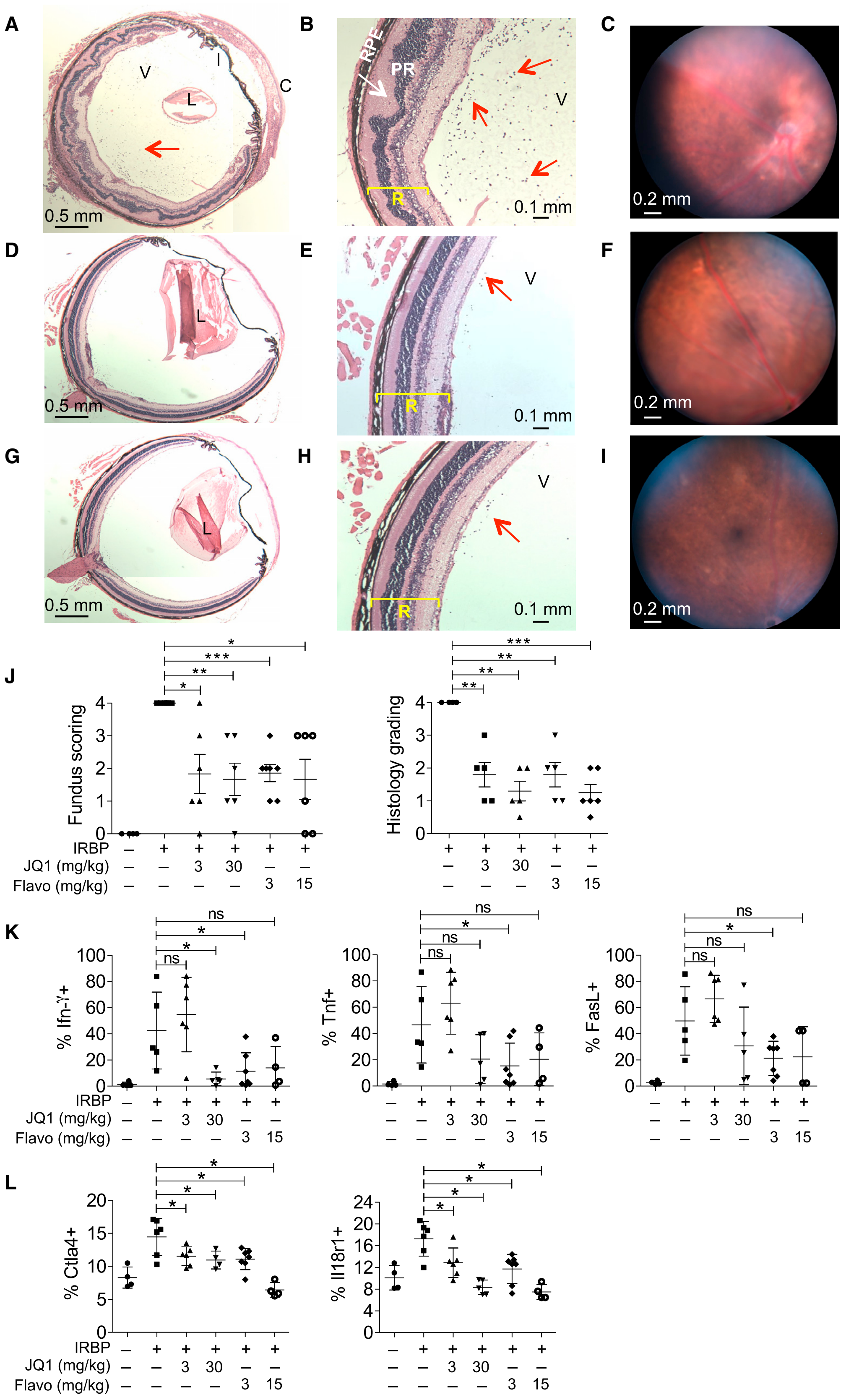
A
V
I
C
L
0.5 mm
B
RPE
PR
V
R
0.1 mm
C
0.2 mm
D
L
0.5 mm
E
V
R
0.1 mm
F
0.2 mm
G
L
0.5 mm
H
V
R
0.1 mm
I
0.2 mm
J
Fundus scoring
Histology grading
IRBP
JQ1 (mg/kg)
Flavo (mg/kg)
K
% Ifn-γ+
% Tnf+
% FasL+
ns
L
% Ctla4+
% Il18r1+

### Strand-Specific RNA-Seq

Poly-adenylated RNA was isolated with Oliogtex (QIAGEN). rRNA was depleted from total RNA with Ribo-Zero Gold (EpiCentre). Libraries were prepared using the Illumina Directional mRNA-Seq Sample Prep and the NEBNext Multiplex Small RNA Library Prep kits and then sequenced on an Illumina HiSeq 2500. RNA-seq reads were filtered for quality and to remove adapters, aligned to hg19 using TopHat2, and transcripts identified with Cufflinks v2.1.1 using default settings.

### EAU

B10.RIII mice were immunized subcutaneously with 300 μg $IRBP_{161–180}$ (Cambridge Peptides) and monitored by fundoscopy on days 8–9 (Agarwal et al., 2012; Gardner et al., 2013). Flavopiridol (3 and 15 mg/kg) and JQ1 (3 and 30 mg/kg) were administered by daily intraperitoneal injection and disease progression scored by retinal fundoscopy and histology at days 14–15. Enucleated eyes were fixed, sectioned, stained with eosin, and counterstained with hematoxylin and graded (Agarwal et al., 2012). Single cell suspensions were prepared from retinas or inguinal lymph nodes for flow cytometry. The significance of changes in disease scoring and in the number of cells expressing T-bet target genes were assessed with a t test.

### shRNA Knockdown

Naive mouse CD4+ T cells were activated under Th1 polarizing conditions and transduced with pMY-Thy1.1-miR-30 retrovirus expressing shRNAs targeting firefly luciferase, Aff4, Med1, or Med17. After 3 days, Thy1.1+ cells were purified by magnetic cell sorting. On day 7, cells were either restimulated with 2 μg/ml anti-CD3/CD28 for 6 hr or left unstimulated.

## ACCESSION NUMBERS

The accession number for the ChIP-seq, RNA-seq, and microarray data reported in this paper is GEO: GSE62486.

## SUPPLEMENTAL INFORMATION

Supplemental Information includes Supplemental Experimental Procedures, seven figures, and seven tables and can be found with this article online at http://dx.doi.org/10.1016/j.celrep.2016.05.054.

## AUTHOR CONTRIBUTIONS

R.G.J. and G.M.L. initiated the research program. A.H., C.M.E., M.E., P.A., P.L., D.J.C., V.L.C., G.M.L., and R.G.J. designed experiments. A.H., C.M.E., M.E., J.C.H.L., K.O., I.J., A.K., D.J.C., and R.G.J. performed experiments and interpreted data. R.G.J. led the bioinformatics work, with some analyses performed by A.H., C.M.E., and J.A. R.G.J. oversaw the research. A.H. and R.G.J. wrote the paper, with input from all authors.

## ACKNOWLEDGMENTS

We are grateful to Jay Bradner for the kind gift of JQ1. Sequencing was performed by UCL Genomics and the UCL Cancer Institute Genomics Core Facility, supported by the CRUK - UCL Centre. This work was funded by a Wellcome Trust grant to R.G.J. and G.M.L. (WT091009); a BBSRC grant to R.G.J. and G.M.L. (BB/L009277/1); an MRC Career Development award to R.G.J. (G0802068); and MRC grants to D.J.C. and P.L. (G0400503, G1000758, G953693) and R.G.J. and G.M.L. (MR/M003493/1). This work was supported by the NIHR UCLH Biomedical Research Centre and by the NIHR Clinical Research Facility at Guy's and St. Thomas' NHS Foundation Trust and NIHR Biomedical Research Centre based at Guy's and St. Thomas' NHS Foundation Trust and King's College London. D.J.C. also acknowledges financial support from NIHR Leicester Respiratory Biomedical Research Unit. The views expressed are those of the author(s) and not necessarily those of the NHS, the NIHR, or the Department of Health.

Received: October 24, 2014
Revised: April 21, 2016
Accepted: May 9, 2016
Published: June 9, 2016

## REFERENCES

Agarwal, R.K., Silver, P.B., and Caspi, R.R. (2012). Rodent models of experimental autoimmune uveitis. Methods Mol. Biol. *900*, 443–469.

Balasubramani, A., Shibata, Y., Crawford, G.E., Baldwin, A.S., Hatton, R.D., and Weaver, C.T. (2010). Modular utilization of distal cis-regulatory elements controls Ifng gene expression in T cells activated by distinct stimuli. Immunity *33*, 35–47.

Bandukwala, H.S., Gagnon, J., Togher, S., Greenbaum, J.A., Lamperti, E.D., Parr, N.J., Molesworth, A.M., Smithers, N., Lee, K., Witherington, J., et al. (2012). Selective inhibition of CD4+ T-cell cytokine production and autoimmunity by BET protein and c-Myc inhibitors. Proc. Natl. Acad. Sci. USA *109*, 14532–14537.

Barboric, M., Nissen, R.M., Kanazawa, S., Jabrane-Ferrat, N., and Peterlin, B.M. (2001). NF-kappaB binds P-TEFb to stimulate transcriptional elongation by RNA polymerase II. Mol. Cell *8*, 327–337.

Brown, J.D., Lin, C.Y., Duan, Q., Griffin, G., Federation, A.J., Paranal, R.M., Bair, S., Newton, G., Lichtman, A.H., Kung, A.L., et al. (2014). NF-κB directs dynamic super enhancer formation in inflammation and atherogenesis. Mol. Cell *56*, 219–231.

Carlsten, J.O., Zhu, X., and Gustafsson, C.M. (2013). The multitalented Mediator complex. Trends Biochem. Sci. *38*, 531–537.

Chapuy, B., McKeown, M.R., Lin, C.Y., Monti, S., Roemer, M.G., Qi, J., Rahl, P.B., Sun, H.H., Yeda, K.T., Doench, J.G., et al. (2013). Discovery and characterization of super-enhancer-associated dependencies in diffuse large B cell lymphoma. Cancer Cell *24*, 777–790.

**Figure 7. Alleviation of Uveitis by Inhibition of Transcriptional Elongation**

(A and B) Representative histopathology from an IRBP-immunized mouse treated with vehicle control (n = 6) showing severe EAU (grade 4) (choroid: C; iris: I; lens: L; vitreous: V, A; retinal layer: R; photoreceptors: PR; and retinal pigment epithelium: RPE, B) (yellow lines, retinal layers; red arrows, infiltrating cells; and white arrows, retina folding). Image in (A) is composed of two fields of view.

(C) Representative in vivo fundoscopy of an IRBP-immunized mouse treated with vehicle control, exhibiting large confluent lesions with retinal atrophy.

(D and E) As in (A) and (B), except for mice treated with JQ1 (30 mg/kg) for 5 days. (D) is composed of two fields of view.

(F) As in (C), except for mice treated with JQ1 (30 mg/kg) for 5 days. Only very small, peripheral focal lesions were present.

(G and H) As in (A) and (B), except for mice treated with Flavopiridol (15 mg/kg) for 5 days. (G) is composed of two fields of view.

(I) As in (C), except for mice treated with Flavopiridol (15 mg/kg) for 5 days.

(J) Mean (± SEM) fundoscopy scores for control mice and mice immunized with IRBP with and without treatment with JQ1 and Flavopiridol (left). The EAU retinal histology scores are shown (right) (*$p < 0.05$, **$p < 0.01$, and ***$p < 0.005$) (unpaired t test with Welch's correction, two-tailed).

(K) Percentage of IFNγ+, TNFα+, and FasL+ CD4+CD3+ T cells from the retina of non-immunized and IRBP-immunized mice treated with carrier, JQ1, or Flavopiridol (mean ± SD) (*$p < 0.05$ and not significant: ns) (one-tailed Student's t test).

(L) As in (K), except for Il18r1+ and Ctla4+ CD4+CD3+ populations from the inguinal lymph nodes.

See also Figure S7.

Di Micco, R., Fontanals-Cirera, B., Low, V., Ntziachristos, P., Yuen, S.K., Lovell, C.D., Dolgalev, I., Yonekubo, Y., Zhang, G., Rusinova, E., et al. (2014). Control of embryonic stem cell identity by BRD4-dependent transcriptional elongation of super-enhancer-associated pluripotency genes. Cell Rep. *9*, 234–247.

Donner, A.J., Ebmeier, C.C., Taatjes, D.J., and Espinosa, J.M. (2010). CDK8 is a positive regulator of transcriptional elongation within the serum response network. Nat. Struct. Mol. Biol. *17*, 194–201.

Gardner, P.J., Joshi, L., Lee, R.W., Dick, A.D., Adamson, P., and Calder, V.L. (2013). SIRT1 activation protects against autoimmune T cell-driven retinal disease in mice via inhibition of IL-2/Stat5 signaling. J. Autoimmun. *42*, 117–129.

Hatton, R.D., Harrington, L.E., Luther, R.J., Wakefield, T., Janowski, K.M., Oliver, J.R., Lallone, R.L., Murphy, K.M., and Weaver, C.T. (2006). A distal conserved sequence element controls Ifng gene expression by T cells and NK cells. Immunity *25*, 717–729.

Hawkins, R.D., Larjo, A., Tripathi, S.K., Wagner, U., Luu, Y., Lönnberg, T., Raghav, S.K., Lee, L.K., Lund, R., Ren, B., et al. (2013). Global chromatin state analysis reveals lineage-specific enhancers during the initiation of human T helper 1 and T helper 2 cell polarization. Immunity *38*, 1271–1284.

Hnisz, D., Abraham, B.J., Lee, T.I., Lau, A., Saint-André, V., Sigova, A.A., Hoke, H.A., and Young, R.A. (2013). Super-enhancers in the control of cell identity and disease. Cell *155*, 934–947.

Huang, B., Yang, X.D., Zhou, M.M., Ozato, K., and Chen, L.F. (2009). Brd4 coactivates transcriptional activation of NF-kappaB via specific binding to acetylated RelA. Mol. Cell. Biol. *29*, 1375–1387.

Hwang, E.S., Hong, J.H., and Glimcher, L.H. (2005). IL-2 production in developing Th1 cells is regulated by heterodimerization of RelA and T-bet and requires T-bet serine residue 508. J. Exp. Med. *202*, 1289–1300.

Jang, M.K., Mochizuki, K., Zhou, M., Jeong, H.S., Brady, J.N., and Ozato, K. (2005). The bromodomain protein Brd4 is a positive regulatory component of P-TEFb and stimulates RNA polymerase II-dependent transcription. Mol. Cell *19*, 523–534.

Kanhere, A., Hertweck, A., Bhatia, U., Gökmen, M.R., Perucha, E., Jackson, I., Lord, G.M., and Jenner, R.G. (2012). T-bet and GATA3 orchestrate Th1 and Th2 differentiation through lineage-specific targeting of distal regulatory elements. Nat. Commun. *3*, 1268.

Koch, F., Fenouil, R., Gut, M., Cauchy, P., Albert, T.K., Zacarias-Cabeza, J., Spicuglia, S., de la Chapelle, A.L., Heidemann, M., Hintermair, C., et al. (2011). Transcription initiation platforms and GTF recruitment at tissue-specific enhancers and promoters. Nat. Struct. Mol. Biol. *18*, 956–963.

Lam, M.T., Li, W., Rosenfeld, M.G., and Glass, C.K. (2014). Enhancer RNAs and regulated transcriptional programs. Trends Biochem. Sci. *39*, 170–182.

Lazarevic, V., Glimcher, L.H., and Lord, G.M. (2013). T-bet: a bridge between innate and adaptive immunity. Nat. Rev. Immunol. *13*, 777–789.

Lin, C., Garruss, A.S., Luo, Z., Guo, F., and Shilatifard, A. (2013). The RNA Pol II elongation factor Ell3 marks enhancers in ES cells and primes future gene activation. Cell *152*, 144–156.

Lovén, J., Hoke, H.A., Lin, C.Y., Lau, A., Orlando, D.A., Vakoc, C.R., Bradner, J.E., Lee, T.I., and Young, R.A. (2013). Selective inhibition of tumor oncogenes by disruption of super-enhancers. Cell *153*, 320–334.

Mele, D.A., Salmeron, A., Ghosh, S., Huang, H.R., Bryant, B.M., and Lora, J.M. (2013). BET bromodomain inhibition suppresses TH17-mediated pathology. J. Exp. Med. *210*, 2181–2190.

Messi, M., Giacchetto, I., Nagata, K., Lanzavecchia, A., Natoli, G., and Sallusto, F. (2003). Memory and flexibility of cytokine gene expression as separable properties of human T(H)1 and T(H)2 lymphocytes. Nat. Immunol. *4*, 78–86.

Miller, S.A., Huang, A.C., Miazgowicz, M.M., Brassil, M.M., and Weinmann, A.S. (2008). Coordinated but physically separable interaction with H3K27-demethylase and H3K4-methyltransferase activities are required for T-box protein-mediated activation of developmental gene expression. Genes Dev. *22*, 2980–2993.

Murphy, K.M., and Stockinger, B. (2010). Effector T cell plasticity: flexibility in the face of changing circumstances. Nat. Immunol. *11*, 674–680.

Nakayamada, S., Kanno, Y., Takahashi, H., Jankovic, D., Lu, K.T., Johnson, T.A., Sun, H.W., Vahedi, G., Hakim, O., Handon, R., et al. (2011). Early Th1 cell differentiation is marked by a Tfh cell-like transition. Immunity *35*, 919–931.

Natoli, G., and Andrau, J.C. (2012). Noncoding transcription at enhancers: general principles and functional models. Annu. Rev. Genet. *46*, 1–19.

Nicodeme, E., Jeffrey, K.L., Schaefer, U., Beinke, S., Dewell, S., Chung, C.W., Chandwani, R., Marazzi, I., Wilson, P., Coste, H., et al. (2010). Suppression of inflammation by a synthetic histone mimic. Nature *468*, 1119–1123.

O'Shea, J.J., and Paul, W.E. (2010). Mechanisms underlying lineage commitment and plasticity of helper CD4+ T cells. Science *327*, 1098–1102.

Parker, S.C., Stitzel, M.L., Taylor, D.L., Orozco, J.M., Erdos, M.R., Akiyama, J.A., van Bueren, K.L., Chines, P.S., Narisu, N., Black, B.L., et al. (2013). Chromatin stretch enhancer states drive cell-specific gene regulation and harbor human disease risk variants. Proc. Natl. Acad. Sci. USA *110*, 17921–17926.

Peeters, J.G., Vervoort, S.J., Tan, S.C., Mijnheer, G., de Roock, S., Vastert, S.J., Nieuwenhuis, E.E., van Wijk, F., Prakken, B.J., Creyghton, M.P., et al. (2015). Inhibition of super-enhancer activity in autoinflammatory site-derived T cells reduces disease-associated gene expression. Cell Rep. *12*, 1986–1996.

Schoenborn, J.R., Dorschner, M.O., Sekimata, M., Santer, D.M., Shnyreva, M., Fitzpatrick, D.R., Stamatoyannopoulos, J.A., and Wilson, C.B. (2007). Comprehensive epigenetic profiling identifies multiple distal regulatory elements directing transcription of the gene encoding interferon-gamma. Nat. Immunol. *8*, 732–742.

Sharma, M., George, A.A., Singh, B.N., Sahoo, N.C., and Rao, K.V. (2007). Regulation of transcript elongation through cooperative and ordered recruitment of cofactors. J. Biol. Chem. *282*, 20887–20896.

Shnyreva, M., Weaver, W.M., Blanchette, M., Taylor, S.L., Tompa, M., Fitzpatrick, D.R., and Wilson, C.B. (2004). Evolutionarily conserved sequence elements that positively regulate IFN-gamma expression in T cells. Proc. Natl. Acad. Sci. USA *101*, 12622–12627.

Sica, A., Dorman, L., Viggiano, V., Cippitelli, M., Ghosh, P., Rice, N., and Young, H.A. (1997). Interaction of NF-kappaB and NFAT with the interferon-gamma promoter. J. Biol. Chem. *272*, 30412–30420.

Smith, E., and Shilatifard, A. (2014). Enhancer biology and enhanceropathies. Nat. Struct. Mol. Biol. *21*, 210–219.

Spitz, F., and Furlong, E.E. (2012). Transcription factors: from enhancer binding to developmental control. Nat. Rev. Genet. *13*, 613–626.

Stubbington, M.J., Mahata, B., Svensson, V., Deonarine, A., Nissen, J.K., Betz, A.G., and Teichmann, S.A. (2015). An atlas of mouse CD4(+) T cell transcriptomes. Biol. Direct *10*, 14.

Szabo, S.J., Kim, S.T., Costa, G.L., Zhang, X., Fathman, C.G., and Glimcher, L.H. (2000). A novel transcription factor, T-bet, directs Th1 lineage commitment. Cell *100*, 655–669.

Takahashi, H., Parmely, T.J., Sato, S., Tomomori-Sato, C., Banks, C.A., Kong, S.E., Szutorisz, H., Swanson, S.K., Martin-Brown, S., Washburn, M.P., et al. (2011). Human mediator subunit MED26 functions as a docking site for transcription elongation factors. Cell *146*, 92–104.

Vahedi, G., Takahashi, H., Nakayamada, S., Sun, H.W., Sartorelli, V., Kanno, Y., and O'Shea, J.J. (2012). STATs shape the active enhancer landscape of T cell populations. Cell *151*, 981–993.

Vahedi, G., Kanno, Y., Furumoto, Y., Jiang, K., Parker, S.C., Erdos, M.R., Davis, S.R., Roychoudhuri, R., Restifo, N.P., Gadina, M., et al. (2015). Super-enhancers delineate disease-associated regulatory nodes in T cells. Nature *520*, 558–562.

van Essen, D., Engist, B., Natoli, G., and Saccani, S. (2009). Two modes of transcriptional activation at native promoters by NF-kappaB p65. PLoS Biol. *7*, e73.

Wang, W., Yao, X., Huang, Y., Hu, X., Liu, R., Hou, D., Chen, R., and Wang, G. (2013). Mediator MED23 regulates basal transcription in vivo via an interaction with P-TEFb. Transcription *4*, 39–51.

Wei, G., Wei, L., Zhu, J., Zang, C., Hu-Li, J., Yao, Z., Cui, K., Kanno, Y., Roh, T.Y., Watford, W.T., et al. (2009). Global mapping of H3K4me3 and H3K27me3 reveals specificity and plasticity in lineage fate determination of differentiating CD4+ T cells. Immunity *30*, 155–167.

Wei, G., Abraham, B.J., Yagi, R., Jothi, R., Cui, K., Sharma, S., Narlikar, L., Northrup, D.L., Tang, Q., Paul, W.E., et al. (2011). Genome-wide analyses of transcription factor GATA3-mediated gene regulation in distinct T cell types. Immunity *35*, 299–311.

Whyte, W.A., Orlando, D.A., Hnisz, D., Abraham, B.J., Lin, C.Y., Kagey, M.H., Rahl, P.B., Lee, T.I., and Young, R.A. (2013). Master transcription factors and mediator establish super-enhancers at key cell identity genes. Cell *153*, 307–319.

Wienerroither, S., Shukla, P., Farlik, M., Majoros, A., Stych, B., Vogl, C., Cheon, H., Stark, G.R., Strobl, B., Müller, M., and Decker, T. (2015). Cooperative transcriptional activation of antimicrobial genes by STAT and NF-κB pathways by concerted recruitment of the Mediator complex. Cell Rep. *12*, 300–312.

Yang, Z., Yik, J.H., Chen, R., He, N., Jang, M.K., Ozato, K., and Zhou, Q. (2005). Recruitment of P-TEFb for stimulation of transcriptional elongation by the bromodomain protein Brd4. Mol. Cell *19*, 535–545.

Zhang, W., Prakash, C., Sum, C., Gong, Y., Li, Y., Kwok, J.J., Thiessen, N., Pettersson, S., Jones, S.J., Knapp, S., et al. (2012). Bromodomain-containing protein 4 (BRD4) regulates RNA polymerase II serine 2 phosphorylation in human CD4+ T cells. J. Biol. Chem. *287*, 43137–43155.

Zhu, J., Yamane, H., and Paul, W.E. (2010). Differentiation of effector CD4 T cell populations (*). Annu. Rev. Immunol. *28*, 445–489.

Zhu, J., Jankovic, D., Oler, A.J., Wei, G., Sharma, S., Hu, G., Guo, L., Yagi, R., Yamane, H., Punkosdy, G., et al. (2012). The transcription factor T-bet is induced by multiple pathways and prevents an endogenous Th2 cell program during Th1 cell responses. Immunity *37*, 660–673.

**Cell Reports, Volume *15***

## Supplemental Information

# T-bet Activates Th1 Genes through Mediator

# and the Super Elongation Complex

**Arnulf Hertweck, Catherine M. Evans, Malihe Eskandarpour, Jonathan C.H. Lau, Kristine Oleinika, Ian Jackson, Audrey Kelly, John Ambrose, Peter Adamson, David J. Cousins, Paul Lavender, Virginia L. Calder, Graham M. Lord, and Richard G. Jenner**

# Figure S1, related to Figure 1

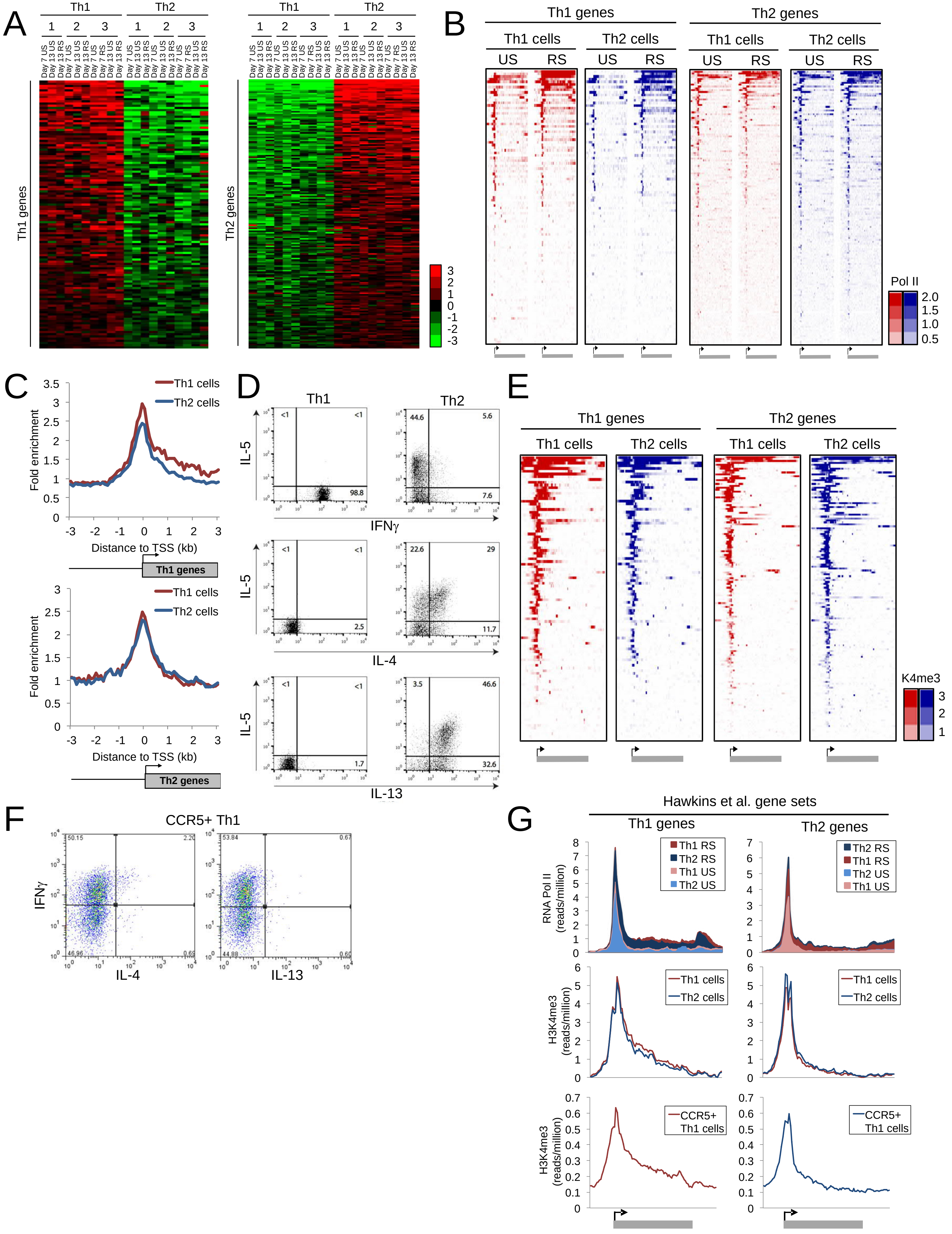

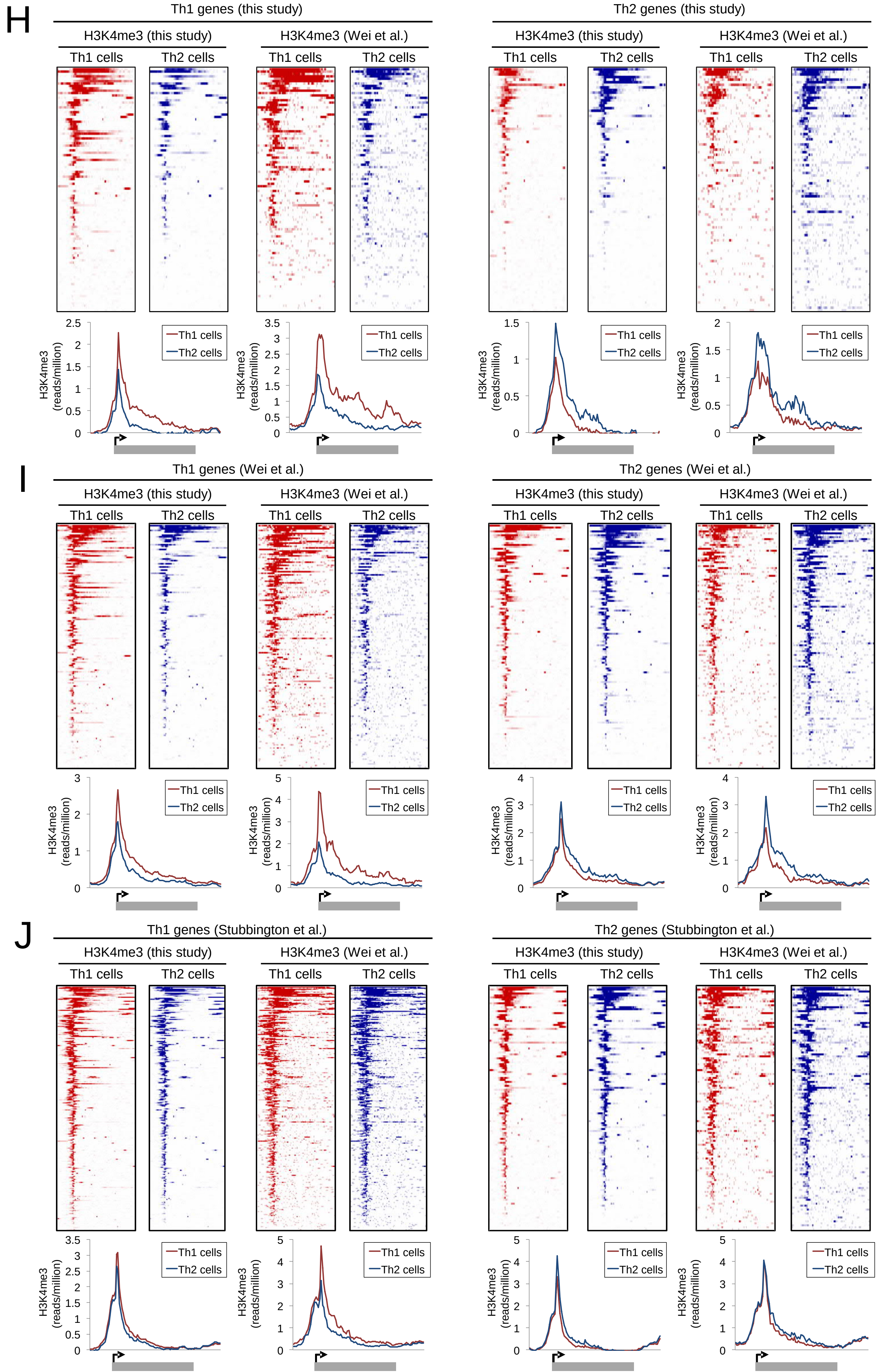
H
Th1 genes (this study)
H3K4me3 (this study)
H3K4me3 (Wei et al.)
Th1 cells
Th2 cells
H3K4me3 (reads/million)
Th2 genes (this study)
I
Th1 genes (Wei et al.)
Th2 genes (Wei et al.)
J
Th1 genes (Stubbington et al.)
Th2 genes (Stubbington et al.)

# Figure S2, related to Figure 2

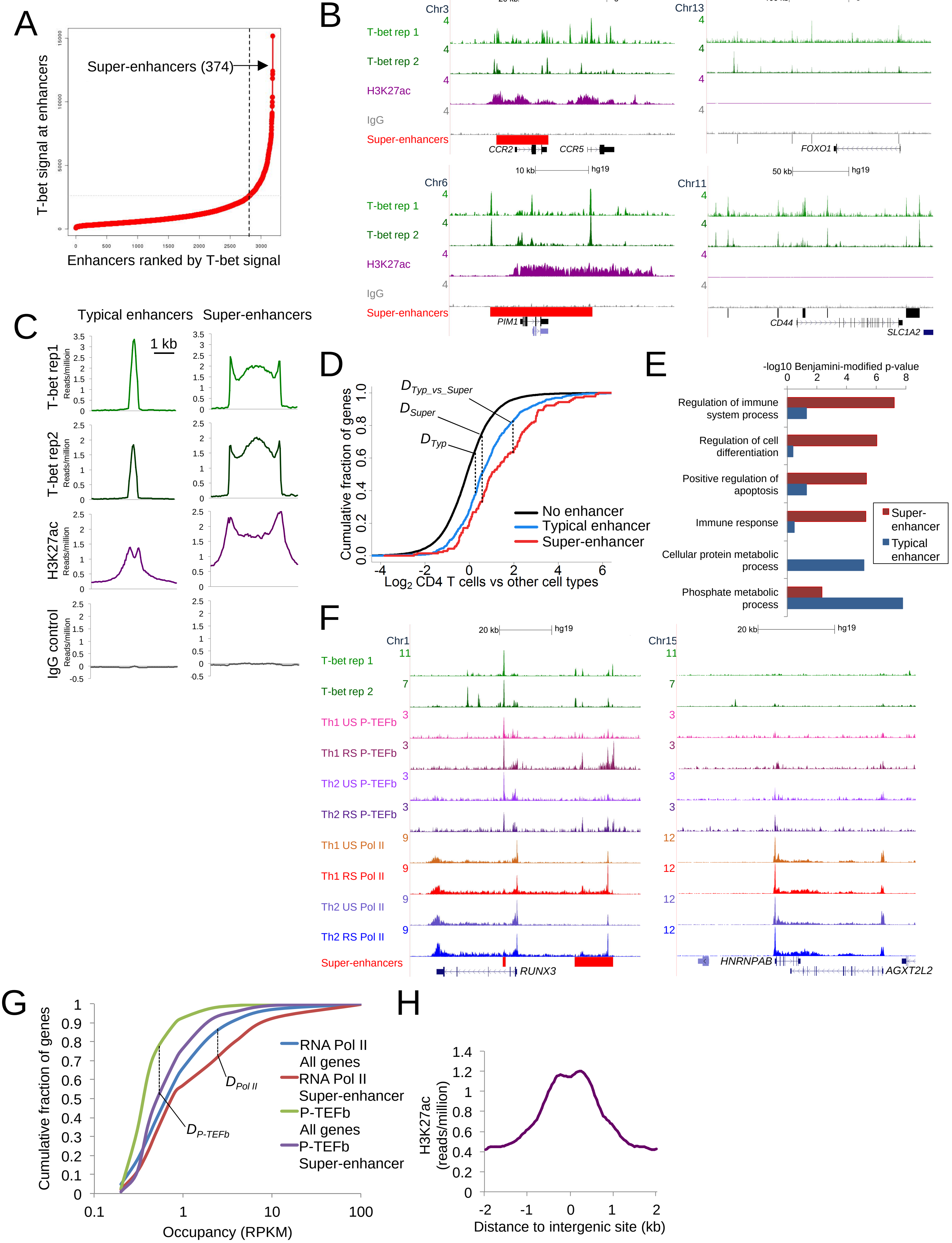

Figure S3, related to Figure 3

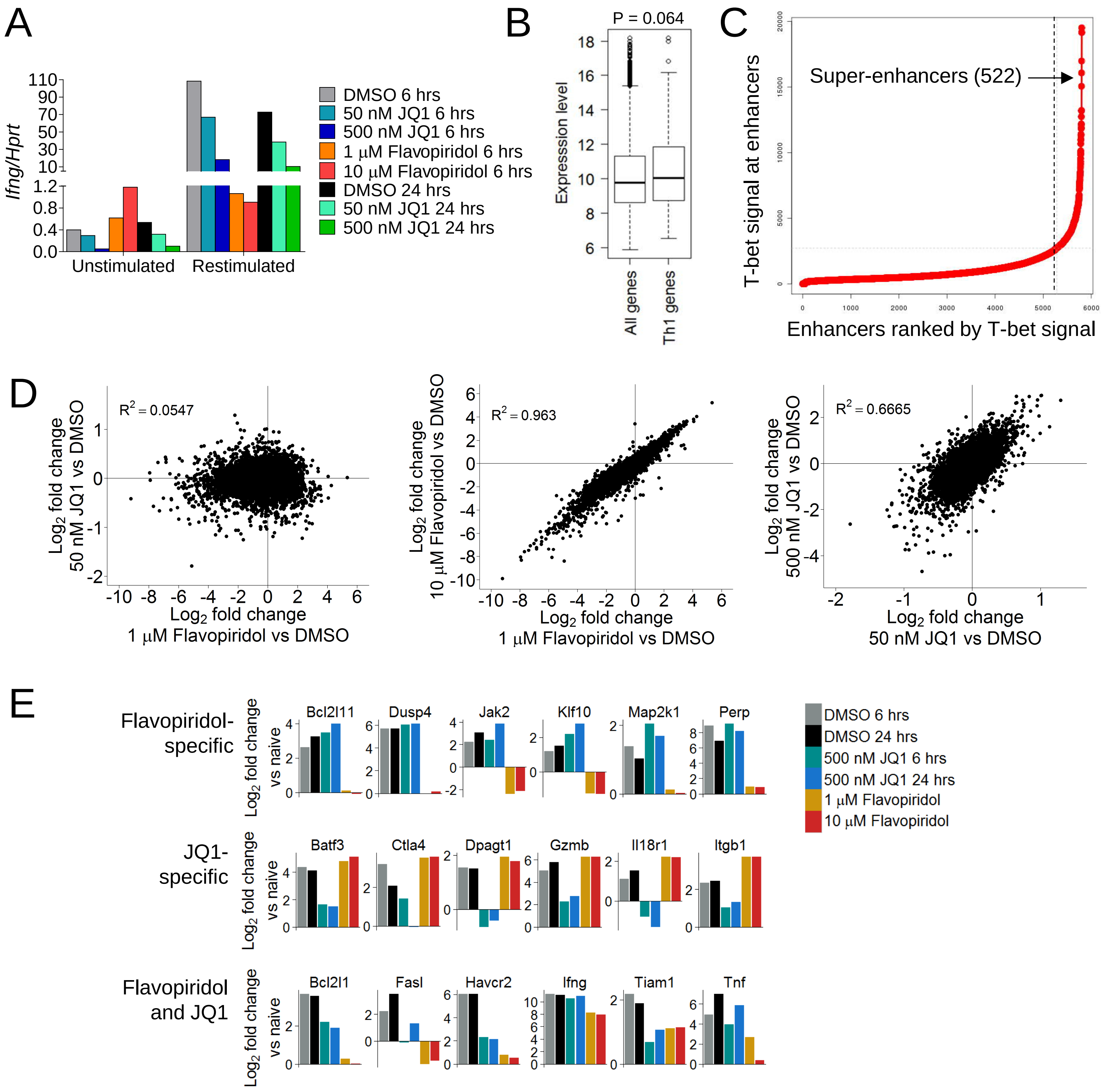

Figure S4, related to Figure 4

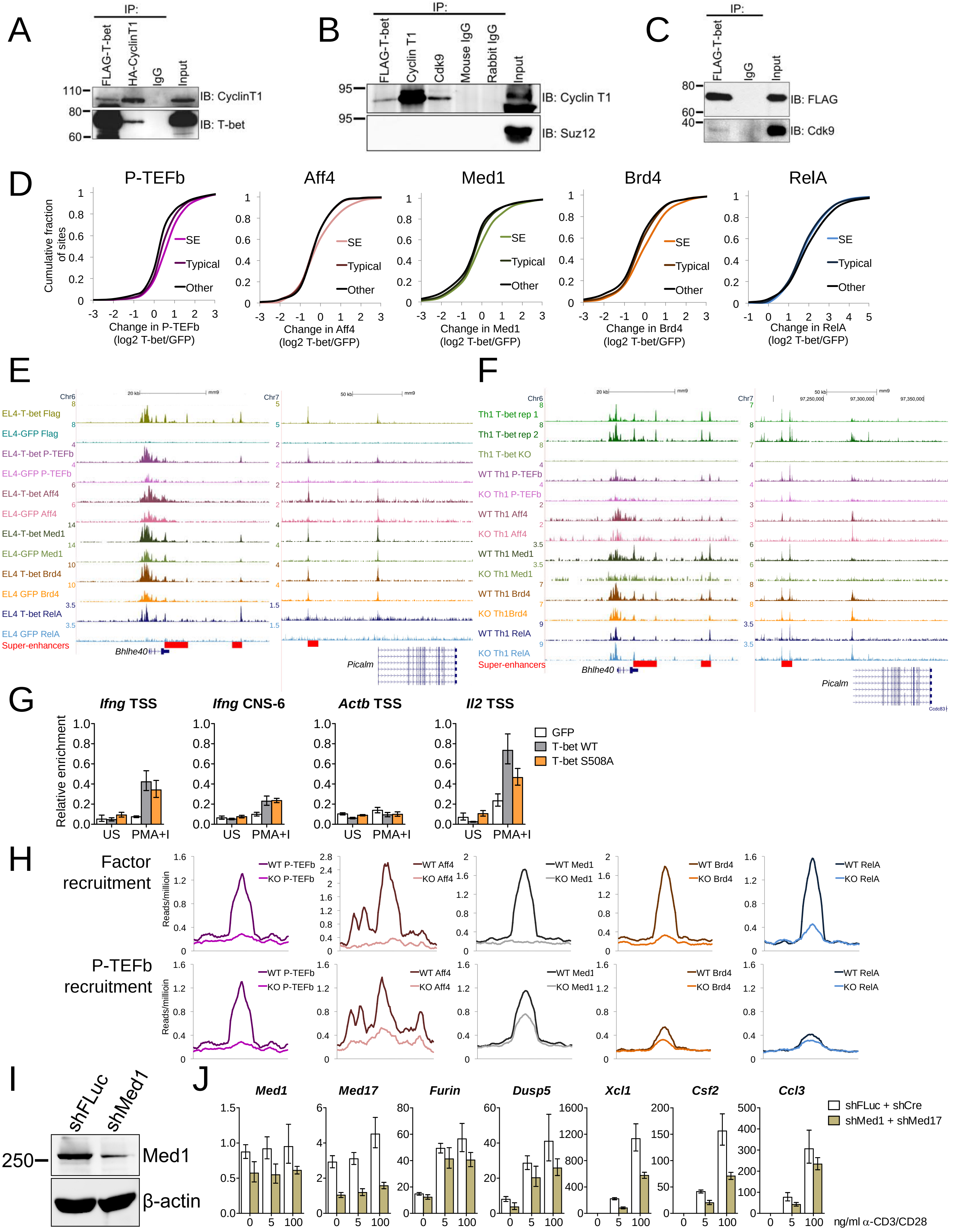

Figure S5, related to Figure 5

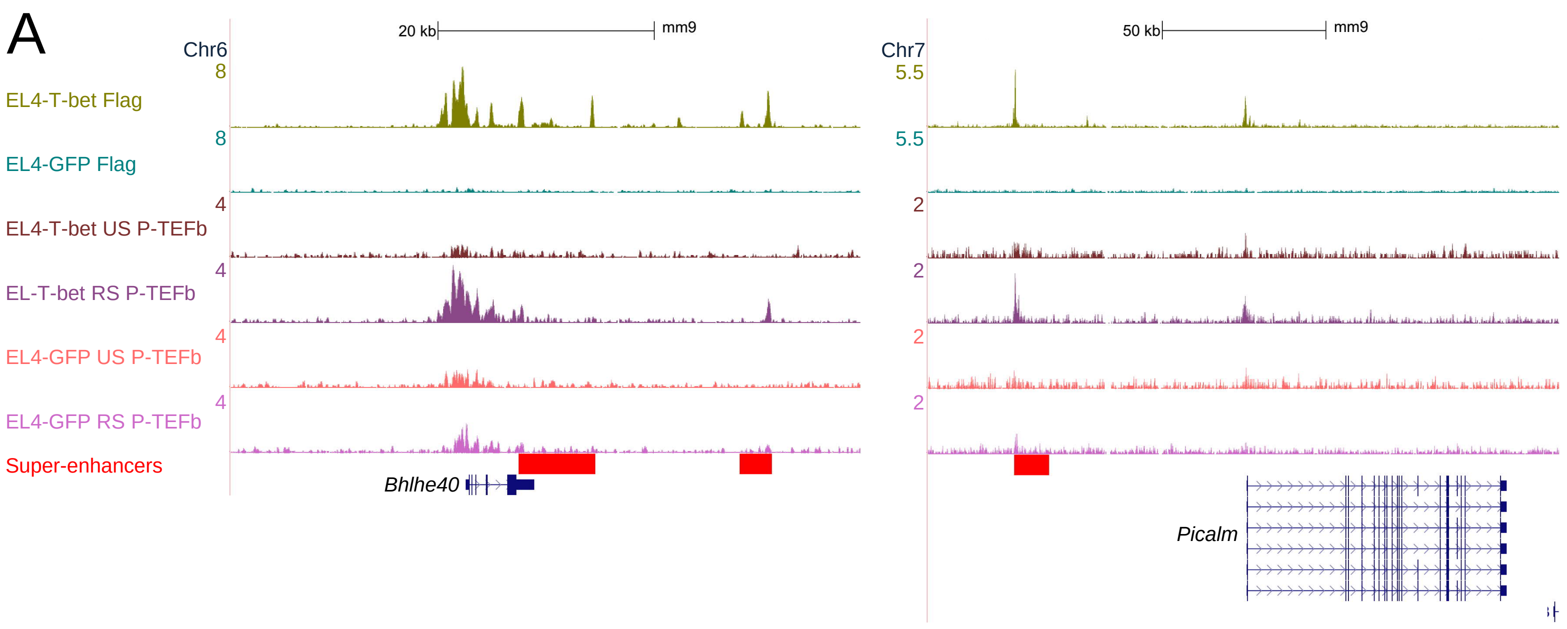


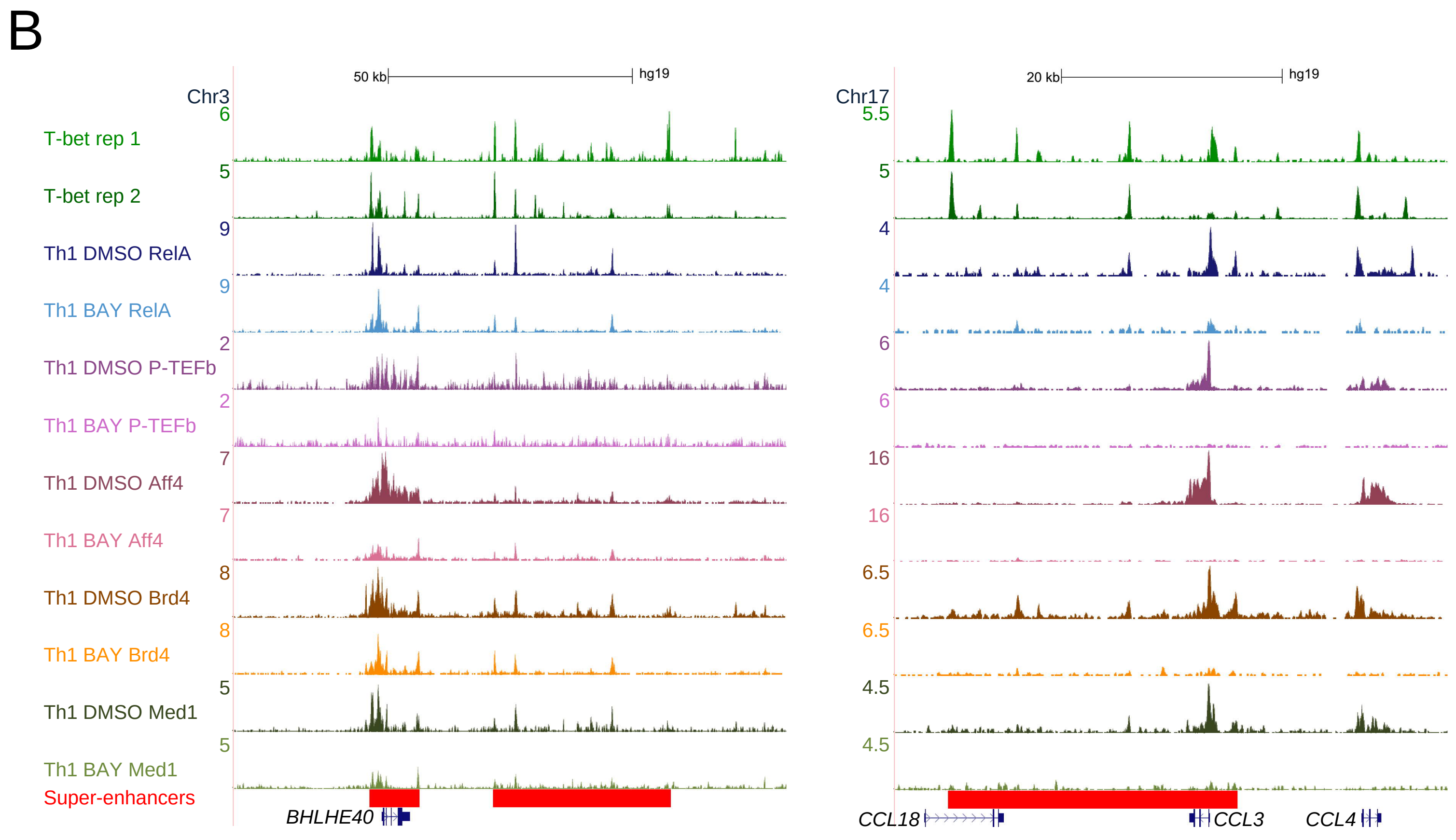

Figure S6, related to Figure 6

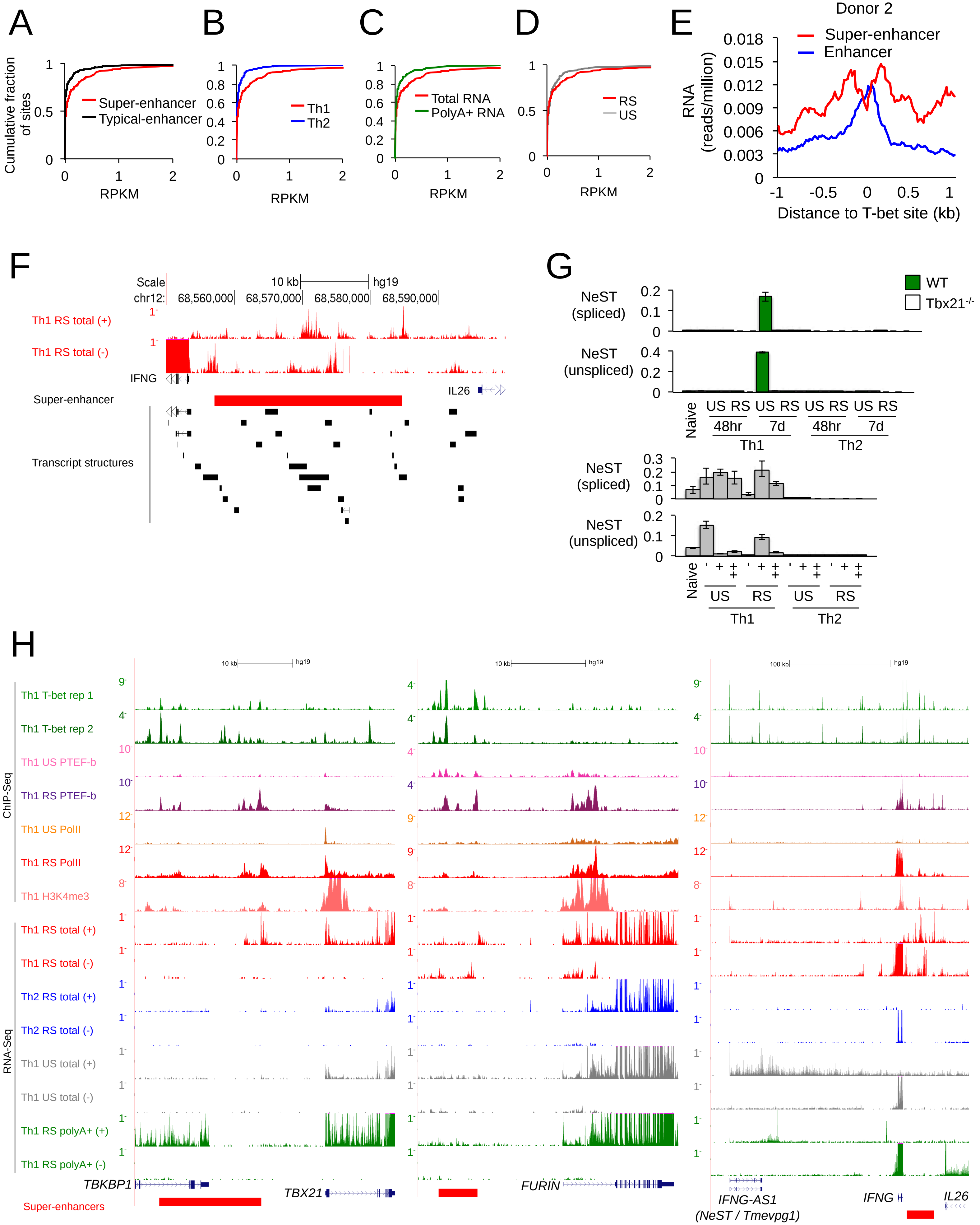

Figure S7, related to Figure 7

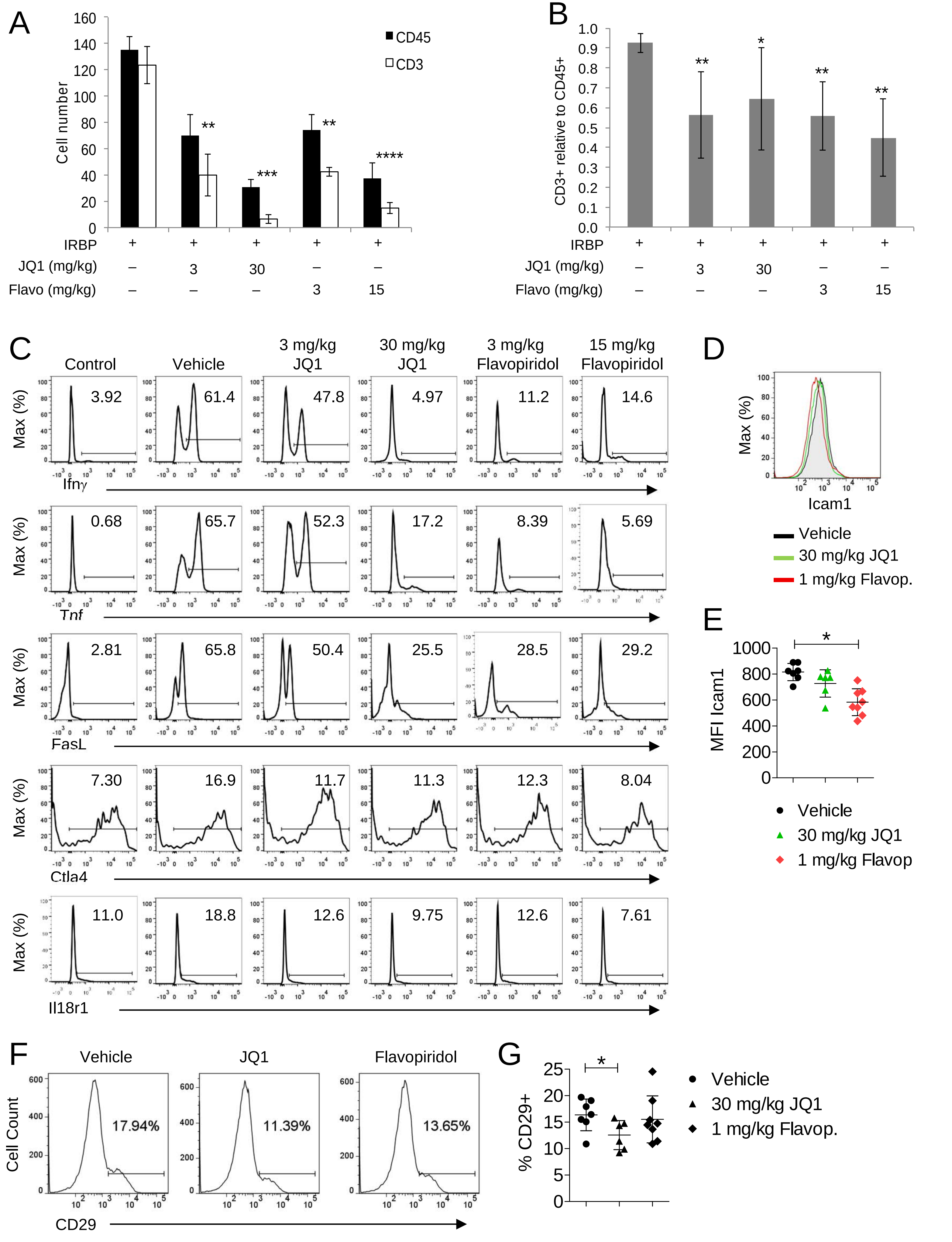

## SUPPLEMENTAL FIGURE LEGENDS

**Figure S1, related to Figure 1. RNA pol II and H3K4me3 in *in vitro* differentiated human and mouse Th1 and Th2 cells and *in vivo* polarized human CCR5+ cells.**

**A.** Gene expression relative to naïve in Th1 and Th2 cells polarised *in vitro* from naïve cells purified from 3 donors and harvested at days 7 or 13 before (US) or after restimulation (RS) with anti-CD3/CD28 antibodies (n=10 Th1 samples, 10 Th2 samples). Genes are divided into those significantly more highly expressed in Th1 cells versus Th2 cells ("Th1 genes"; left panel, pfp<0.05, Rank sum test) or significantly more highly expressed in Th2 cells versus Th1 cells "Th2 genes"; right panel). All genes are also upregulated versus naïve cells by > 2-fold. Gene expression is represented by a color, with the scale (log2 ratio to naïve) shown at the far right.

**B.** Heat maps showing RNA pol II at each individual Th1 genes (left) and Th2 genes (right) that are averaged in Figure 1A. Each row represents a gene. The arrow under graph indicates transcriptional start site and the grey bar the gene body. RNA pol II occupancy is indicated by color intensity, according to the scale on the right.

**C.** Average enrichment profile of the initiation form of RNA pol II (detected by the 8WG16 antibody) at Th1 genes (top) and Th2 genes (bottom) in Th1 cells (red) and Th2 cells (blue). The plot shows average fold-enrichment (normalized signal from ChIP-enriched DNA divided by the signal from input DNA). The start and direction of transcription of the average gene is indicated by an arrow.

**D.** Staining for IFN-γ, IL4, IL5 and IL13 in human *in vitro* polarised Th1 and Th2 cells used for H3K4me3 ChIP-seq.

**E.** As B., except for H3K3me3 in unstimulated Th1 and Th2 cells.

**F.** Staining for IFN-γ, IL4 and IL13 in human CCR5+ memory Th1 cells purified from PBMC and used for H3K4me3 ChIP-seq.

**G.** Top: Average enrichment profile for RNA pol II in unstimulated (US) and restimulated (RS) Th1 and Th2 cells at Th1 and Th2 gene sets previously defined by Hawkins and colleagues (Hawkins et al., 2013). Middle: Average enrichment profile for H3K4me3 in Th1 and Th2 cells at Hawkins *et al.* Th1 and Th2 gene sets. Bottom: Average enrichment profile for H3K4me3 in CCR5+ Th1 cells at Hawkins *et al.* Th1 and Th2 gene sets.

**H.** Heat maps (top) and average enrichment profiles (bottom) for H3K4me3 across gene bodies +/- 2 kb in murine Th1 and Th2 cells at Th1 and Th2 gene sets identified in this study. Murine Th1 and Th2 H3K4me3 ChIP-seq data is from this study or from the study of Wei and colleagues (Wei et al., 2009).

**I.** As H., except at Th1 and Th2 gene sets derived from the RNA-seq data of Wei and colleagues (We et al., 2011).

**J.** As H., except at Th1 and Th2 gene sets previously defined by Stubbington and colleagues (Stubbington et al., 2015).

**Figure S2, related to Figure 2. Identification of T-bet super-enhancers in human T cells and their association with P-TEFb.**

**A.** Distribution of T-bet ChIP-seq signal (input-subtracted total reads) across 3,191 T-bet Th1 enhancers in humans from duplicate ChIP-seq experiments, generated by ROSE (Hnisz et al., 2013). T-bet occupancy is not evenly distributed across the enhancer regions, with a subset of enhancers to the right of the point of inflection (the 374 super-enhancers) containing especially high amounts of T-bet.

**B.** ChIP-seq binding profiles (reads/million, input subtracted) for T-bet and IgG control and for H3K27ac (total H3 subtracted) at example loci associated either with a super-enhancer (left, red bars) or typical enhancer (right, black bars). Scale bars are shown above each panel.

**C.** Average ChIP-seq density (reads/million, input subtracted) for T-bet, H3K27ac and IgG control at typical T-bet enhancers and super-enhancers in human Th1 cells.

**D.** Cumulative frequency distribution of log2 gene expression ratios (CD4 T cell versus average of all other cell types) for genes bound by T-bet at the TSS and associated with a typical T-bet enhancer (n=1041), T-bet super-enhancer (n=219) or neither enhancer type (n=12,221). Distances (*D*) are marked with black dashed lines. Typical enhancer vs all genes $D_{Typ}$=0.28, p<$2.2 \times 10^{-16}$, Super-enhancers vs all genes $D_{Super}$=0.43, p<$2.2 \times 10^{-16}$, Typical enhancer vs super enhancer $D_{Typ\_vs\_Super}$=0.19, p=$3.2 \times 10^{-4}$ (Kolmogorov-Smirnov (K-S) test).

**E.** Significance (-log10 Benjamini-modified p-value) of the enrichment of biological process gene ontology categories in the set of genes associated with T-bet super-enhancers versus typical enhancers.

**F.** ChIP-seq binding profiles (reads/million, input subtracted) for T-bet, P-TEFb and RNA pol II in unstimulated (US) and restimulated (RS) Th1 and Th2 cells at the super-enhancer-associated gene *RUNX3* and the housekeeping gene *HNRNPAB.*

**G.** Cumulative frequency distributions of RNA pol II and P-TEFb occupancy (reads per kb per million total reads) in restimulated Th1 cells at genes associated with super-enhancers (n=322), compared with all genes (n=13,338). $D_{PolII}$=0.15, p=2.4x$10^{-6}$ ; $D_{P\text{-}TEFb}$=0.31, p<2.2x$10^{-16}$ (K-S test).

**H.** Average number of ChIP-seq reads for H3K27ac (reads/million, total H3 subtracted) in restimulated Th1 cells, centred on intergenic P-TEFb binding sites.

**Figure S3, related to Figure 3. Gene expression profiling of murine Th1 and Th2 cells treated with JQ1 and Flavopiridol.**

**A.** Quantitative RT-PCR for Ifng in Th1 cells treated with JQ1, Flavopiridol and DMSO control.

**B.** Absolute average expression (probe fluorescent intensity) between all expressed genes (n=8,095) and Th1-specific genes (n=291). P>0.05, unpaired Student's t-test.

**C.** Distribution of T-bet ChIP-seq signal (input-subtracted total reads) across 5,804 T-bet Th1 enhancers in mice from duplicate ChIP-seq experiments, generated by ROSE (Hnisz et al., 2013). T-bet occupancy is not evenly distributed across the enhancer regions, with a subset of enhancers to the right of the point of inflection (the 522 super-enhancers) containing especially high amounts of T-bet.

**D.** Left panel: Change in gene expression in Th1 cells treated with Flavopiridol (1 μM) versus JQ1 (50 nM). Middle panel: Change in gene expression in Th1 cells treated with 1 μM Flavopiridol versus 10 μM Flavopiridol. Right panel: Change in gene expression in restimulated Th1 cells treated with 50 nM JQ1 versus 500 nM JQ1.

**E.** Expression of selected genes (relative to naïve cells) in Th1 cells treated with DMSO, JQ1 or Flavopiridol. Genes are divided into those repressed by Flavopiridol only, JQ1 only or both Flavopiridol and JQ1.

**Figure S4, related to Figure 4. T-bet is necessary for P-TEFb, Mediator and SEC recruitment to genes and enhancers in activated cells.**

**A.** Co-immunoprecipitation of FLAG-T-bet and HA-cyclinT1 in 293 cells.

**B.** Co-immunoprecipitation of endogenous cyclinT1 with FLAG-T-bet in EL4 T cells. CyclinT1 is also precipitated by its P-TEFb partner CDK9, as expected. Suz12 is not precipitated by FLAG-T-bet or CDK9, and serves as a negative control.

**C.** Co-immunoprecipitation of endogenous cyclinT1 with FLAG-T-bet in primary mouse Th0 cells.

**D.** Cumulative distribution frequency of the change in P-TEFb, Aff4, Med1, Brd4, and RelA occupancy between EL4-T-bet and EL4-GFP cells at super-enhancers and associated genes (SE), at typical enhancers and associated genes (Typical) and at other sites (Other).

**E.** ChIP-seq binding profiles for T-bet, P-TEFb, RelA, Brd4, Med1 and Aff4 in EL4 cells stably expressing GFP alone or expressing T-bet and GFP and stimulated with PMA and ionomycin.

**F.** As B., except for WT and T-bet$^{-/-}$ Th1 cells restimulated with PMA and ionomycin.

**G.** RelA binding (versus input, mean and SD, n=3 technical replicates) at the indicated genomic locations in PMA + ionomycin stimulated EL4 cells expressing wild-type T-bet, T-bet S508A or GFP alone.

**H.** Top: Average binding profiles of P-TEFb, Aff4, Med1, Brd4 or RelA at the set of T-bet dependent sites for each factor in WT (darker colour) and KO (T-bet$^{-/-}$, lighter colour) cells. Bottom: Average P-TEFb binding profiles at the same locations shown above.

**I** Med1 and β-actin protein levels in Th1 cells transduced with retroviruses encoding shRNAs targeting luciferase and *Med1*. Due to a lack of specific antibodies, Med17 and Aff4 knockdown were confirmed at the RNA level only (Figures 4F and S4J).

**J.** Expression of *Med1* and *Med17* and the super-enhancer-associated genes *Furin, Dusp5, Xcl1, Csf2* and *Ccl3* relative to *Hprt* (mean and SD, n=3 technical replicates) in unstimulated and restimulated Th1 cells transduced with retroviruses encoding shRNAs targeting luciferase and Cre (white) or *Med1* and *Med17* (brown).

**Figure S5, related to Figure 5. Restimulation and NF-κB activity are required for recruitment of the elongation machinery to T-bet target genes.**

**A.** ChIP-seq binding profiles for P-TEFb at the *Bhlhe40* and *Picalm* loci in EL4-GFP and EL4-T-bet cells with and without restimulation with PMA and ionomycin.

**B.** ChIP-seq binding profiles for RelA, P-TEFb, Brd4, Med1 and Aff4 at the *BHLHE40* and *CCL3*/*CCL4* loci in restimulated human Th1 cells with and without treatment with BAY 11-7082 (20 µM).

**Figure S6, related to Figure 6. eRNA transcription from super-enhancers at Th1 genes.**

**A.** Cumulative frequency distribution of reads per kilobase per million total reads (RPKM) for total RNA in restimulated Th1 cells within T-bet binding sites and regions spanning 1 kb up- and downstream. T-bet binding sites are divided into those located within intergenic super-enhancers (n=269) or those within intergenic typical-enhancers (n=908). D=0.21, $P<10^{-3}$ (K-S test).

**B.** As A., except comparing total RNA reads around T-bet peaks within intergenic super-enhancers between restimulated Th1 and restimulated Th2 cells. D=0.21, $p<10^{-3}$ (K-S test).

**C.** As A., except comparing total and mRNA RNA reads around T-bet peaks within intergenic super-enhancers. D=0.17, p=0.001 (K-S test).

**D.** As A., except comparing total RNA reads around T-bet peaks within intergenic super-enhancers between unstimulated Th1 and restimulated Th1 cells. D=0.12, p=0.041 (K-S test).

**E.** As Figure 6A, except for total RNA purified from restimulated Th1 cells from a second donor.

**F.** Transcripts assembled by Cufflinks at the *IFNG* super-enhancer.

G. Top: Quantitative RT-PCR for the spliced and unspliced forms of NeST (Ifng-as1, Tmevpg1) relative to Hprt (mean and SD, n=3 technical replicates) in WT and T-bet-/- naïve mouse T cells and US and RS Th1 and Th2 cells polarised for 48 hrs or 7 days. Bottom: As Top, except for WT cells treated with 50 (+) or 500 nM (++) JQ1 (left) or treated with 1 (+) or 10 µM (++) Flavopiridol for 6 hours (right).

**H.** Total RNA and mRNA-seq data (reads/million) at *TBX21*, *FURIN* and *IFNG-AS1/IFNG* showing production of non-poly-adenylated RNAs from super-enhancers at Th1 genes. The strandedness of the RNA is indicated by + (Watson strand) or – (Crick strand). ChIP-seq binding profiles are shown above and are aligned with the RNA-seq data. The positions of super-enhancers are shown as red bars below.

**Figure S7, related to Figure 7**. **Reduction in retinal T cell infiltration and expression of inflammatory genes in CD4+ cells in mice treated with JQ1 and Flavopiridol.**

**A.** Number of CD3+ and CD45+ cells (mean and SD, n≥4) in three separate retinal fields in mice treated with carrier control, JQ1 or Flavopriridol. ** $p<0.01$, *** $p<0.\ 001$, **** $p<0.0001$ (2-sided t-test).

**B.** Number of CD3+ cells relative to the number of CD45+ cells in the samples described in A. * $p<0.05$, ** $p<0.01$ (2-sided t-test).

**C.** Representative cytofluorimetric analysis of retinal CD3+CD4+ T cells (for IFNγ, TNFα and FasL) and CD3+CD4+ T cells purified from the inguinal lymph node (for Ctla4 and Il18r1) from non-immunised and IRBP-immunised mice treated with carrier, JQ1 or Flavopiridol. Cells were permeabilized and stained intracellularly for detection of IFNγ, TNFα and Ctla4. Numbers adjacent to outlined areas indicate the percentage of cells.

**D.** Representative cytofluorimetric analysis of Icam1 expression on CD3+CD4+ T cells purified from the inguinal lymph node after treatment with vehicle only, JQ1 (30 mg/kg) or Flavopiridol (1mg /kg).

**E.** Summary and quantification of cytofluorimetric data shown in (D). Each symbol represents an individual animal; horizontal lines indicate the mean and error bars denote SD. MFI, median florescence intensity. * $p<0.05$ (2-sided t-test).

**F.** Representative cytofluorimetric analysis of CD29 surface expression on CD4+ T cells purified from the inguinal lymph node after treatment with vehicle only, JQ1 (30 mg/kg) or Flavopiridol (1mg /kg).

**G.** Summary and quantification of cytofluorimetric data shown in (F). Each symbol represents an individual animal; horizontal lines indicate the mean and error bars denote SD. * $p<0.05$ (2-sided t-test).

## SUPPLEMENTAL TABLES

**Table S1. Th1 and Th2 genes identified by gene expression profiling (xls file), related to Figure 1**

Worksheet 1: Genes significantly more highly expressed in human Th1 or Th2 cells after 13 days polarization (Agilent human G3 arrays). Worksheet 2: Genes significantly more highly expressed in human Th1 or Th2 cells after 28 days polarization (Affymetrix U133 plus 2 arrays).

**Table S2. T-bet super-enhancers in human and mouse Th1 cells (xls file), related to Figure 2**

Coordinates of human and mouse T-bet super-enhancers and the closest RefSeq gene TSS. Some super-enhancers are associated with more than one gene (gene TSS lie within super-enhancers).

**Table S3. Genes exhibiting differential responses to JQ1 and Flavopiridol (xls file), related to Figure 3**

Genes repressed in reactivated mouse Th1 cells upon by Flavopiridol but not JQ1, JQ1 but not Flavopiridol or by both compounds. Each gene set is provided in an individual tab of the table.

**Table S4. P-TEFb, RelA, Med1, Aff4, Brd4 and H3K4me3 binding sites in EL4-T-bet and EL4-GFP cells (xls file), related to Figure 4**

The number of sequencing reads at each binding site is given, along with the position of the sites relative to murine T-bet super-enhancers and their associated genes (genes with closest TSS), and typical enhancers and their associated genes.

**Table S5. P-TEFb, RelA, Med1, Aff4 and Brd4 binding sites in WT and T-bet-/- Th1 cells (xls file), related to Figure 4**

The number of sequencing reads at each binding site is given, along with the position of the sites relative to murine T-bet super-enhancers and their associated genes (genes with closest TSS), and typical enhancers and their associated genes.

**Table S6. P-TEFb, RelA, Med1, Aff4 and Brd4 binding sites in restimulated human Th1 cells treated with DMSO or BAY 11-7082 (xls file), related to Figure 5**

The number of sequencing reads at each binding site is given, along with the position of the sites relative to human T-bet super-enhancers and their associated genes (genes with closest TSS), and typical enhancers and their associated genes.

**Table S7. T-bet super-enhancer eRNAs in human Th1 and Th2 cells (xls file), related to Figure 6**

Coordinates of eRNAs identified in from total RNA-seq in restimulated Th1 cells and their fragments per kilobase per million total reads (FPKM) values from total and poly-A+ RNA-seq in unstimulated (US) and restimulated (RS) Th1 and Th2 cells. Super-enhancer IDs correspond to the nearest RefSeq gene TSS. Some super-enhancers are associated with more than one gene and some genes are associated with more than one super-enhancer (delineated A, B). eRNA transcripts within each super-enhancer are numbered individually.

## SUPPLEMENTAL EXPERIMENTAL PROCEDURES

### Cells

Purified T cells were obtained from buffy coats (National Blood Service) or blood samples from healthy volunteers. Informed consent was obtained from all subjects, and blood was collected and processed with the approval of and in accordance with the King's College Ethics Committee guidelines (06/Q0705/20). Naïve human CD4+ T-cells (CD4+CD45RA+CD45RO-CD25-CCR7+) were isolated by negative immunomagnetic selection (Miltenyi Biotec) followed by sorting using a FACSAria II (BD Bioscience). Cells were activated for 72 hours by plate bound anti-CD3 and anti-CD28 antibodies (2 µg/ml, BD Pharmingen) and were then cultured for 10 days with rhIL-2 (Biolegend, 10ng/ml). Conditions for T cell polarisation were: rhIL-12 (10 ng/ml, Biolegend) and anti-IL-4 (10 µg/ml, R&D) for Th1, rhIL-4 (10 ng/ml, Biolegend) and anti-IFN-γ (10 µg/ml, R&D) for Th2. For ChIP, cells were formaldehyde crosslinked on day 13 either before (unstimulated) or after (restimulated) treatment with PMA and ionomycin. For gene expression microarray analysis and RNA-seq, samples were taken from naïve cells and Th1 and Th2 cells before and after restimulation with anti-CD3 and anti-CD28 antibodies on days 7 and 13 after purification and this was performed for 3 separate donors (sample size standard for differential gene expression analysis). For experiments testing the role of NF-κB, cells were restimulated for 5 hours with PMA/ionomycin in the presence of 20 µM BAY 11-7082 (Calbiochem) or DMSO before fixation.

For H3K4me3 ChIP-seq, CD4+ naive T-cells were differentiated into either Th1 or Th2 cells *in vitro* for 28 days, as described (Cousins et al., 2002). Cells were activated for 4 hrs with 5 ng/ml PMA (Sigma) and 500 ng/ml ionomycin (CN Biosciences) and assessed for intracellular cytokine staining.

Naïve murine CD4+ T-cells (CD4+CD25-CD62LhighCD44low) were purified from pooled spleen and lymph node cell suspensions of WT and T-bet$^{-/-}$ mice by immunomagnetic selection (Miltenyi Biotec) followed by sorting. Cells were activated for 72 hours with plate-bound anti-CD3 and anti-CD28 monoclonal antibodies (both 2 µg/ml) and then cultured for 5 days in the presence of 20 ng/ml IL-2 (Biolegend). Conditions for T cell polarisation were: 20 ng/ml IL-12

(eBioscience) and 5 μg/ml anti-IL-4 (BioXCell) for Th1 or 20 ng/ml IL-4 (eBioscience) and 10 μg/ml anti-IFN-γ (BioXCell) for Th2. For gene expression analysis, cells were harvested before and after restimulation with anti-CD3/CD28 at 48 hours and 7 days.

Human CCR5+ memory Th1 cells were enriched with CD4 microbeads (Miltenyi Biotech) and CCR5+ Th1 (anti-CCR5, BD Biosciences) effector memory cells isolated by flow cytometry, activated with plate-bound anti-CD3 and anti-CD28 for 4 days, and expanded in media containing IL-2, as described (Messi et al., 2003).

EL4-GFP and EL4-T-bet cells were described in (Kanhere et al., 2012). EL4 cells expressing a form of T-bet insufficient in interaction with RelA were generated as described previously (Kanhere et al. 2012) using the coding region of T-bet containing a S508A point mutation (Hwang et al. 2005). Cells were either incubated with DMSO (unstimulated control) or stimulated with PMA (50 ng/ml) and ionomycin (1 μM) for 5 hours before formaldehyde crosslinking.

**Mice used to harvest cells for ChIP and *in vitro* gene expression analysis**

Wild type (WT) C57BL/6 mice were purchased from Charles River Laboratories International (Margate, UK). T-bet$^{-/-}$ mice (on a C57BL/6 background) were purchased from Taconic (Ejby, Denmark). Mice were bred in the Biological Services Unit at KCL (UK Home Office project license PPL/70/6792) or at Charles River Laboratories International (Margate, UK) analyzed between 6 and 12 weeks of age.

**ChIP-seq**

***Sample preparation***

ChIP was performed as described (Kanhere et al., 2012). Cells were crosslinked by the addition of one-tenth volume of fresh 11% formaldehyde solution for 20 minutes at room temperature before the reaction was quenched by addition of glycine. Cells were rinsed twice with 1xPBS and flash frozen in liquid nitrogen. Cells were lysed with non-ionic detergent, nuclei washed and then lysed with ionic detergent. For RNA pol II ChIP, an alternative set of lysis and wash buffers were

used (Rahl et al., 2010). Cells were sonicated on ice to solubilize and shear crosslinked DNA (24W for 10 x 30 second pulses using a Misonix Sonicator 3000). The resulting whole cell extract was cleared by centrifugation and then incubated overnight at 4°C with 100 µl of Dynal Protein G magnetic beads that had been pre-incubated with 10 µg of purified antibody or, for the case of T-bet, 10 µl of purified serum (see table below). Beads were washed 6 times with RIPA buffer and 1 time with TE containing 50 mM NaCl. Bound complexes were eluted from the beads by heating at 65°C with occasional vortexing and crosslinks then reversed in IP and input DNA by overnight incubation at 65°C. IP and input DNA were then purified by treatment with RNase A, proteinase K and phenol:chloroform extraction followed by ethanol precipitation.

H3K4me3 ChIP was performed on native chromatin. Chromatin was prepared by using the protocol of Feil and colleagues (http://www.epigenome-noe.net/researchtools/protocol.php?protid=2) with some minor modifications. Mono and dinucleosomal chromatin was recovered from nuclei treated with micrococcal nuclease (10U/µl for 7 mins) and chromatin quality assessed by agarose gel elecrophoresis and semi-quantitated using a nanodrop. ChIP for H3K4me3 and total H3 was performed with Protein G beads (Active Motif). DNA was purified by phenol/chloroform phase separation, ethanol precipitation, followed by clean up with Qiagen PCR purification columns.

Libraries were constructed from ChIP and input DNA by standard Illumina protocols, except that DNA in the range 150-350bp was gel-purified after PCR-amplification. The libraries were quantified using a Qubit and Agilent bioanalyzer, pooled and subjected to 35 or 50 bp single-end read sequencing with an Illumina GAIIx or HiSeq 2500 sequencer.

**ChIP-seq datasets used in this study.**

US – not restimulated before crosslinking, RS – restimulated with PMA/ionomycin before crosslinking.

| ***Species*** | ***Cells*** | ***Condition*** | ***Factor*** | ***Antibody*** | ***Accession (if previously published)*** |
|---|---|---|---|---|---|
| Human | Th1 | US | T-bet | 9856 (custom) (Jenner et al., 2009) | GSM776557 GSM776555 |
| Human | Th1 | US | T-bet | SY4530 (custom) | |
| Human | Th1 | US | Rabbit IgG | Abcam 46540 | |
| Human | Th1 | RS | H3K27ac | Abcam ab4729 | |
| Human | Th2 | RS | H3K27ac | Abcam ab4729 | |
| Human | Th1 | US | H3K4me3 | Abcam ab8580 | |
| Human | Th2 | US | H3K4me3 | Abcam ab8580 | |
| Human | CCR5+ memory Th1 | US | H3K4me3 | Abcam ab8580 | |
| Human | Th1 | US | RNA pol II | Santa-Cruz N-20 (sc-899) | |
| Human | Th1 | RS | RNA pol II | As above | |
| Human | Th2 | US | RNA pol II | As above | |
| Human | Th2 | RS | RNA pol II | As above | |
| Human | Th1 | US | P-TEFb | Pool of CyclinT1 T-18 (sc-8127), C-20 (sc-8128), CDK9 H-169 (sc-8338) and CDK9 C-20 (sc-484) | |
| Human | Th1 | RS | P-TEFb | As above | |
| Human | Th2 | US | P-TEFb | As above | |
| Human | Th2 | RS | P-TEFb | As above | |
| Human | Th1 | RS and DMSO | RelA | Santa Cruz C-20 (sc-372). Replicated with Abcam ab7970 | |
| Human | Th1 | RS and BAY 11-7082 | RelA | As above | |
| Human | Th1 | RS and DMSO | P-TEFb | Pool of CyclinT1 T-18 (sc-8127), C-20 (sc-8128), CDK9 H-169 (sc-8338) and CDK9 C-20 (sc-484) | |
| Human | Th1 | RS and BAY 11-7082 | P-TEFb | As above | |
| Human | Th1 | RS and DMSO | Aff4 | Bethyl Laboratories (A302-539A) | |
| Human | Th1 | RS and BAY 11-7082 | Aff4 | As above | |
| Human | Th1 | RS and DMSO | Brd4 | Bethyl Laboratories (A301-985A100) | |
| Human | Th1 | RS and BAY 11-7082 | Brd4 | As above | |
| Human | Th1 | RS and DMSO | Med1 | Bethyl Laboratories (A300-793A) | |
| Human | Th1 | RS and BAY 11-7082 | Med1 | As above | |
| Mouse | Th1 | RS | T-bet | 9856 (custom) (Jenner et al., 2009) | GSM998272 GSM998271 |

| | | | | | |
|---|---|---|---|---|---|
| Mouse | Th1 | RS | T-bet | SY4530 (custom) | GSM836124 |
| Mouse | T-bet$^{-/-}$ Th1 | RS | T-bet | 9856 (custom) (Jenner et al., 2009) | GSM998273 |
| Mouse | Th1 | ? | H3K4me3 | Abcam ab8580 | |
| Mouse | Th2 | ? | H3K4me3 | Abcam ab8580 | |
| Mouse | T-bet$^{-/-}$ Th1 | | H3K4me3 | Abcam ab8580 | |
| Mouse | Th1 | | H3K4me3 | | GSM361999 |
| Mouse | Th2 | | H3K4me3 | | GSM362001 |
| Mouse | WT Th1 | RS | P-TEFb | CDK9 H-169 (sc-8338) | |
| Mouse | WT Th2 | RS | P-TEFb | As above | |
| Mouse | T-bet$^{-/-}$ Th1 | RS | P-TEFb | As above | |
| Mouse | EL4+GFP | US | P-TEFb | Pool of CyclinT1 T-18 (sc-8127), C-20 (sc-8128), CDK9 H-169 (sc-8338) and CDK9 C-20 (sc-484) | |
| Mouse | EL4+GFP | RS | P-TEFb | As above | |
| Mouse | EL4+T-bet | US | P-TEFb | As above | |
| Mouse | EL4+T-bet | RS | P-TEFb | As above | |
| Mouse | WT Th1 | RS | Brd4 | Bethyl Laboratories (A301-985A100) | |
| Mouse | T-bet$^{-/-}$ Th1 | RS | Brd4 | As above | |
| Mouse | EL4+GFP | RS | Brd4 | As above | |
| Mouse | EL4+T-bet | RS | Brd4 | As above | |
| Mouse | WT Th1 | RS | RelA | Santa Cruz C-20 (sc-372) | |
| Mouse | T-bet$^{-/-}$ Th1 | RS | RelA | As above | |
| Mouse | EL4+GFP | RS | RelA | As above | |
| Mouse | EL4+T-bet | RS | RelA | As above | |
| Mouse | WT Th1 | RS | Med1 | Bethyl Laboratories (A300-793A) | |
| Mouse | T-bet$^{-/-}$ Th1 | RS | Med1 | As above | |
| Mouse | EL4+GFP | RS | Med1 | As above | |
| Mouse | EL4+T-bet | RS | Med1 | As above | |
| Mouse | WT Th1 | RS | Aff4 | Bethyl Laboratories (A302-539A) | |
| Mouse | T-bet$^{-/-}$ Th1 | RS | Aff4 | As above | |
| Mouse | EL4+GFP | RS | Aff4 | As above | |
| Mouse | EL4+T-bet | RS | Aff4 | As above | |
| Mouse | EL4+GFP | RS | FLAG | Sigma (M2, F1804) | |
| Mouse | EL4+T-bet | RS | FLAG | As above | |

***A note about pooling antibodies for ChIP-seq***

We used a pool of CDK9 and cyclin T1 antibodies for some P-TEFb ChIP-seq experiments. CDK9 and CyclinT1 are not known to function independently of P-TEFb and thus antibodies to both factors only detect P-TEFb with a single binding profile. Pooling antibodies to multiple subunits of the same factor maximizes the signal, and has been used previously by other investigators (eg. Zeitlinger et al., 2007). Pooled monoclonal antibodies in immunoprecipitations enable the formation of multimeric complexes, like polyclonal antibodies, but are more specific than polyclonal antibodies ("Using Antibodies" by Ed Harlow and David Lane).

***A note about the Super-elongation complex***

The super-elongation complex (SEC) has been extensively characterized (He et al., 2011; Lin et al., 2011; Lin et al., 2013; Lin et al., 2010; Luo et al., 2012; Mueller et al., 2009). Aff4 is a core, essential subunit of the complex, is not known to be present in other complexes, and has previously been used as a super-elongation complex marker in ChIP-seq (eg. Lin et al., 2011 and Luo et al., 2012).

***ChIP-seq data replication***

We assessed the quality and reproducibility of our dataset in three ways. Firstly, we performed biological replicate ChIP-seq experiments for T-bet, RNA pol II, P-TEFb, Aff4, Brd4, Med1 and RelA (Santa Cruz sc-372 and Abcam ab7970) in human Th1 cells and for T-bet and Aff4 (Bethyl A302-539A and that used in Lin et al., 2010, kind gift from Ali Shalatifard) in mouse WT and T-bet-/- Th1 cells. We assessed the consistency between the replicates by irreproducible discovery rate (IDR) analysis (Li et al., 2011) and found that, in each case, Np/Nt was less than 2, which is the standard reproducibility threshold used by the ENCODE project (Landt et al., 2012). Secondly, for every ChIP-seq experiment, the specific enrichment of binding sites, along with their T-bet or NF-kB dependence was validated by ChIP-qPCR. Finally, ChIP-seq for T-bet, P-TEFb, RelA, Brd4, Med1 and Aff4 was performed in human Th1, mouse Th1 and mouse EL4-T-bet cells, and in each case, the factors displayed the same characteristic binding profiles regardless of the cell type or species, further confirming the robustness of the data.

### *ChIP-Chip for the initiation form of RNA polymerase II*

ChIP was performed for the initiation form of RNA pol II using the antibody 8WG16 (Abcam ab817) using our standard protocol. ChIP and input DNA were then amplified, labelled and hybridised to custom oligonucleotide microarrays (Agilent) covering 8 kb (approximately 4 kb upstream and 4 kb downstream) around the transcription start site of 18,450 Ref-Seq-annotated human genes, as described (Jenner et al., 2009). Fold enrichment of DNA in ChIP versus input was calculated using a previously described analysis pipeline (Jenner et al., 2009).

### *ChIP-seq data analysis*

Reads (in fastq files) were filtered to remove adapters using fastq-mcf and for quality using seqkt and aligned to the human (hg19) or mouse genome (mm9) with Bowtie2 (default settings). Bigwig files for visualization in the UCSC genome browser were generated using a custom pipeline; duplicate reads were first removed, coverage calculated with genomeCoverageBed and tag density calculated in 10bp windows. unionBedGraphs was then used to subtract input (or total histone H3 for histone ChIPs) signals and bigwig files generated using bedGraphToBigWig. Regions of significant enrichment were identified using MACS version 1.4 (Zhang et al., 2008) using input or total histone H3 as background, with the setting --keep-dup=1. A p-value threshold of $10^{-7}$ was used, unless stated otherwise. Binding sites within 2 kb of any RefSeq gene transcription start site (TSS) were identified using closestBed and considered as proximal binding sites and associated with that gene. Other binding sites were divided into those within a RefSeq gene (intragenic) and those outside of a gene (intergenic) using intersectBed.

**Numbers of proximal, intergenic and intragenic binding sites ($p<10^{-7}$, associated with Figure 2C).** The relative distribution of P-TEFb binding sites is also maintained at more stringent MACS p-value thresholds.

| ***Factor or histone modification*** | ***Proximal (<2kb from TSS)*** | ***Distal intragenic*** | ***Distal intergenic*** | ***Total sites*** |
|---|---|---|---|---|
| **RNA pol II** | 11731 | 9813 | 4261 | 25805 |
| **H3K4me3** | 14687 | 4485 | 4936 | 24108 |
| **H3K27ac** | 11467 | 11194 | 7216 | 29877 |
| **P-TEFb** | 1865 | 2131 | 2147 | 6113 |

***Average binding profiles***

Average binding profiles (in reads/million) across sets of genes or enhancers were generated with ngsplot (https://code.google.com/p/ngsplot/ (Shen et al., 2014)) and reads from input or control total H3 ChIPs subtracted. To control for differences in ChIP efficiency when comparing average profiles between ChIPs of the same factor in different cells, average reads/million across a set of genes were then normalized by the average signal across all genes. The relative levels of RNA pol II at Th1 and Th2 genes between Th1 and Th2 cells were calculated by dividing the two ngsplot average profiles.

To determine change in P-TEFb binding at intergenic sites between WT and T-bet$^{-/-}$ cells (Figure 4A), intergenic sites were first defined by MACS as those with $p<10^{-9}$ in WT Th1 cells and located outside of RefSeq genes and satellites and simple repeats (RepeatMasker). Then, ngsplot was used to calculate P-TEFb binding profiles across each site and the maximum value (the peak height) identified. The same analyses method was used to determine change in P-TEFb binding at intergenic sites between stimulated EL4-GFP and EL4-T-bet cells (Figure 4B), except that the set of intergenic P-TEFb sites in stimulated EL4-T-bet cells was used.

***Counts at specific genes or proximal regions***

The numbers of reads at specific genes were counted using featureCounts (http://bioinf.wehi.edu.au/featureCounts/ (Liao et al., 2014)) and converted to reads per kilobase per million (RPKM) using the size of the feature and the total number of aligned reads. The number of reads in the input sample were then subtracted. To compare P-TEFb with RNA pol II (Figure S2G), reads across the longest variant of each RNA pol II or P-TEFb-bound gene were counted. To compare P-TEFb binding in the presence or absence of T-bet (Figure 4A-B), reads were counted over regions spanning -100 to +500bp relative to TSS that were considered bound by P-TEFb by MACS ($p<10^{-7}$).

***Identification of super-enhancers***

Super-enhancers are genomic regions that exhibit high levels of binding by a particular transcriptional regulator. They were first identified by Whyte and colleagues (2013) and Loven and colleagues (2013) for Med1. Super-enhancers have also been identified by the binding of single site-specific transcription factors, for example PU.1 in pro B-cells and MyoD in myotubes by Whyte et al. Super-enhancers bound by T-bet in Th1 cells were identified with the ROSE algorithm (Loven et al., 2013; Whyte et al., 2013; Hnsiz et al., 2013; https://bitbucket.org/young_computation/rose).

The ROSE algorithm seeks to identify regions of the genome with high levels of binding by a particular transcriptional regulator. All the sites bound by the factor of interest are identified by a standard tool, such as MACS, and then those sites lying within 12.5 kb of one another are grouped together to identify bound regions (not including sites that lie within 2.5 kb of gene TSS). Then the numbers of sequencing reads for each binding site (typical enhancer) or grouped binding sites (super-enhancer) are counted. The regions are then ordered by the number of ChIP sequencing reads ("binding") they contain and the number of reads plotted against rank. This produces a curve with an exponential profile whereby a minority of genomic regions have many more sequencing reads (and thus binding of the transcriptional regulator) than others. The threshold used by the ROSE algorithm to define super-enhancers is the point of inflexion in the relationship between enhancer rank and transcription regulator occupancy (measured by ChIP-seq reads), as shown in Figure S2A (for human) and S3C (for mouse).

***Human T-bet.*** For human, T-bet binding sites were identified from two biological replicate ChIPs, performed using two independent antibodies, using MACS, which identified 10,358 sites at $p<10^{-7}$ for one replicate and 18,219 sites $p<10^{-9}$ for the other (after removal of a small number of peaks at satellites using intersectBed). IntersectBed was then used to identify binding sites identified in both replicates (4,821 sites) and ROSE employed with its default settings to identify enhancers by stitching together binding sites located within 12.5 kb of each other that were at least 2.5 kb from RefSeq-annotated transcription start sites. This resulted in identification of 3191 enhancers. Super-enhancers were then identified by ROSE as those with highest number of reads in the two T-bet replicate ChIP datasets (using a merged Bam file), in comparison to input

sequencing data, as described in (Whyte et al., 2013). The plot of sequencing reads for each stitched T-bet enhancer is shown in Figure S2A. This resulted in identification of 374 T-bet super-enhancers and 2,817 typical enhancers in human Th1 cells. Enhancers were deemed to be associated with the nearest gene TSS (Whyte et al., 2013), identifying 357 genes. To increase confidence in our gene assignment further for Figures S2D and S2E, we used the closest genes that also had a proximal T-bet binding site (MACS $p<10^{-7}$, from Bam files merged from both replicate ChIPs). For humans, this identified 219 genes associated with super-enhancers and 1041 genes associated with typical enhancers.

***Mouse T-bet.*** For mouse, T-bet binding sites were identified from two biological replicate ChIPs, one from our own lab and one from (Nakayamada et al., 2011) (accession GSM836124 at GEO), each performed using different antibodies. MACS identified 13,644 ($p<10^{-7}$) and 32,770 ($p<10^{-9}$) binding sites, respectively. More sites were identified for GSM836124 because no input dataset was available for MACS to use as background. 10,094 sites were present in both datasets (after removal of satellites) and ROSE identified 5282 typical enhancers and 522 super-enhancers (Figure S3C). Mapping these to the closest TSS identified 471 genes.

***Measuring changes in transcription regulator occupancy***

Changes in the binding of transcriptional regulators between WT and T-bet-/- Th1 cells or between EL4-T-bet and EL4-GFP cells were measured using a cumulative distribution frequency analysis. For each transcriptional regulator, binding sites were identified by MACS and filtered to remove any overlapping satellites, ENCODE blacklist regions (https://sites.google.com/site/anshulkundaje/projects/blacklists) or sites for which at >25% corresponded to a simple repeat. For mouse, sites on the Y chromosome were removed because of differences between the ratio of male and female cells in the different cell lysates. MAnorm (Shao et al., 2012) was then used to merge peaks between the two samples under comparison (eg. merge the peaks for Aff4 between WT and T-bet KO). Using BEDtools, we then identified the binding sites for each factor that overlapped T-bet super-enhancers or their associated (closest) genes (“SE” sites), binding sites that didn’t fall into this category but overlapped typical T-bet enhancers (stitched enhancers not classified as super-enhancers by ROSE and their closest genes (“Typical” sites) and then any other binding site outside of these T-bet-associated regions

(“Other” sites). Read numbers under each merged peak were converted to reads per million total reads and log2 ratios calculated (T-bet$^{-/-}$ vs WT, EL4-T-bet vs EL4-GFP or BAY 11-7082 vs DMSO). The cumulative distribution frequency of these changes within each set of sites was then plotted. Thus, for the T-bet$^{-/-}$ vs WT analysis, the further the “SE” and “Typical” lines are shifted to the left relative to the “Other” line, the greater the loss of the factor at T-bet target sites compared to non-T-bet target sites (at which one expects binding to remain constant upon T-bet loss). The significance of the differences in log2 ratios between the different binding site sets (super vs other; typical vs other) were estimated with R using a Mann-Whitney U test and corrected for multiple hypothesis testing by multiplying by the number of transcriptional regulators under test for each cell type.

MAnorm was also used to identify sites at which transcriptional regulators were significantly depleted in T-bet$^{-/-}$ cells versus WT Th1 cells with a threshold of $p<0.001$ and with a fold-change of at least 3-fold. The binding profile of each factor and of P-TEFb at these sites was then plotted with ngsplot (Figure S4H).

**Gene expression microarray analysis**

***Human Th1 and Th2 cells***

Total RNA was purified with Trizol (Life Technologies) from naïve and *in vitro* polarised Th1 and Th2 cells (3 different donors) at days 7 and 13 after purification before and after restimulation. RNA was DNase-treated using DNA-free (Life Technologies) and integrity verified using an Agilent Bioanalyzer. Fifty ng of RNA was labelled with Cy3 and RNA from a common reference RNA pool (Stratagene) was labelled with Cy5 using Agilent’s Low input Quick Amp 2 Color Labelling kits and hybridized together to Agilent Human gene expression G3 DNA microarrays, following the standard protocol. Arrays were scanned on an Agilent High Resolution C scanner and images were quantified using Agilent Feature Extraction Software (version 10.7). Genes significantly higher expressed across all human Th1 cell samples versus all Th2 cell samples, or vice versa, were identified by rank sum test (pfp<0.05) using RankProdIt (http://strep-microarray.sbs.surrey.ac.uk/RankProducts/ (Laing and Smith, 2010)). Th1 and Th2

gene sets were also filtered to only include those at least 2-fold upregulated versus naïve cells in all Th1 samples or all Th2 samples, respectively.

We also polarized human CD4+ T cells for 28 days and performed gene expression microarray analysis and H3K4me3 ChIP-seq. RNA was purified from cells with and without restimulation from 3 donors and labeled and hybridized to GeneChip U133 plus 2 arrays according to the manufacturer's instructions (Affymetrix). Robust multichip average preprocessing was performed and genes significantly overexpressed in Th1 or Th2 cells were identified using the Partek ANOVA model (>2-fold difference, $p<0.05$ including FDR).

To provide an independent data set, we also plotted RNA pol II and H3K4me3 ChIP-seq data at Th1 and Th2 genes from Tables S4A in (Hawkins et al., 2013) (Figure S1G).

***In vitro treatment with Flavopiridol and JQ1 and microarray analysis***

Mouse wild-type naive CD4+ T cells were cultured under Th1 and Th2 polarizing conditions. On day 6 of polarization, $4x10^6$ cells were transferred in medium complemented with 50 nM and 500 nM JQ1 (kindly provided by Jay Bradner) or 1% DMSO vehicle control. On day 7 of polarisation previously untreated cells were cultured in the presence of 50 nM and 500 nM JQ1, 1 µM and 10 µM Flavopiridol or 1% DMSO vehicle control for 2 hrs. These cells and the cells incubated with the drugs for 22 hrs were reactivated with plate-bound αCD3/CD28 (2 µg/ml each) or left unstimulated for 4 hrs. Total RNA was purified with TRIsure (Bioline), DNase-treated using DNA-free (Life Technologies) and integrity verified using an Agilent Bioanalyzer. Two hundred ng total RNA isolated from mouse Th1 and Th2 cells treated with Flavopiridol, JQ1 or DMSO was labeled with Cy3 with the two color Low Input Quick Amp Labeling Kit (Agilent) according to the manufacturer's instructions (with naïve T cell reference RNA labeled with Cy5) and both hybridized together to SurePrint G3 Mouse GE 8x60K microarrays (Agilent). Arrays were scanned as before. Median raw intensity values were background corrected using a normal-exponential convolution model and log2 transformed expression ratios (Cy3 channel vs trimmed mean of Cy5 channel across all arrays) were loess normalised. Expression ratios were first averaged for identical probes and then for identical genes. Genes with probe signal intensities less than 40% higher than the 95th percentile of the negative control probes were removed from the analysis and the remaining 8095 genes were considered to be expressed across the data set.

Lineage-specific genes and groups of genes repressed by JQ1 and/or Flavopiridol in reactivated Th1 cells were defined using the following thresholds: Th1-specific: Th1 DMSO 6 hrs vs Th2 DMSO 6hrs >= 2 (n =291); Th2-specific: Th1 DMSO 6hrs vs Th2 DMSO 6 hrs <= 2 (n= 180); Flavopiridol-specific genes: DMSO 6 and 24 hrs vs naïve >= 2, 1 µM and 10 µM Flavopiridol < 2, 1 µM and 10 µM Flavopiridol vs DMSO 6hrs <= 2, 50 nM and 500 nM JQ1 6hrs vs DMSO 6 hrs >= 1 (n= 126); JQ1-specific genes: DMSO 6 and 24 hrs vs naïve >= 2, 500 nM JQ1 6hrs vs DMSO 6hrs <= 1.5, 500 nM JQ1 24hrs vs DMSO 24hrs <= 1.5, 1 µM and 10 µM Flavopiridol vs DMSO 6hrs >= 1 (n= 147), genes repressed by Flavopiridol and JQ1: DMSO 6 and 24 hrs vs naïve >= 2, 1 µM and 10 µM Flavopiridol vs DMSO 6hrs <= 2, 500 nM JQ1 6hrs vs DMSO 6hrs <= 1.5, 500 nM JQ1 24hrs vs DMSO 24hrs <= 1.5 (n= 247). Significance of differences in gene expression between groups of genes presented in the cumulative distribution frequency plots was determined by a two-tailed Kolmogorov-Smirnov (K-S) test.

***Comparison of CD4+ T cell gene expression to other cell-types***

Data profiling gene expression across 79 human cell and tissue types was obtained (Su et al., 2004) and gene expression in CD4+ T cells calculated relative the median level across all cell types. These values were then plotted as a cumulative distribution for genes associated with a T-bet super-enhancer and proximal binding site and genes associated with a typical T-bet enhancer and proximal binding site and the significance of the difference between them estimated using a K-S test.

**Strand-specific RNA-seq**

***Sample preparation***

Total RNA purified from naïve CD4+ cells and day 13 resting and restimulated Th1 and Th2 cells (2 donors) was obtained from samples also used for microarray analysis. Poly-adenylated RNA was purified from 1 µg of total RNA using the Qiagen Oliogtex kit and ribosomal RNA was depleted from 1 µg of total RNA using Ribo-Zero Gold (EpiCentre), as per manufacturer's instructions. 5' cap structures were removed with tobacco acid pyrophosphatase (TAP, Life Technologies) and RNA fragmented with potassium acetate (100 mM) and magnesium acetate (30 mM) at $94^{o}$C for 3 mins. RNA was then repaired with Antarctic phosphatase and PNK,

according to the instructions in the Illumina Directional mRNA-seq Sample Prep Guide. Libraries were generated using the NEBNext Multiplex Small RNA Library Prep Set for Illumina kit, with 14 cycles of PCR amplification, and products between 130bp and 350bp were gel purified. Libraries were quantified by qPCR (Library Quantification Kit, Kapa Biosystems) and average fragment size determined on an Agilent 2100 Bioanalyzer using DNA HS assays. Libraries were sequenced on an Illumina HiSeq (50bp paired-end).

***Processing and alignment of RNA-seq data***

Reads (in fastq files) were filtered to remove adapters using fastq-mcf (-S, -t 0.0001, -l 20) and for quality using seqkt trimfq (-q 0.01) and aligned to human genome (hg19) with TopHat2 (v2.0.9, --b2-very-sensitive, -g 2, -p 4, --library-type fr-secondstrand, default setting for –mate-inner-dist and --mate-std-dev). Bigwig files for visualization of the data in the UCSC Genome browser were generated from TopHat2 accepted hits bam files using Samtools to select for strand specific reads, which were then converted into Bedgraph summaries using Bedtools genomecov, prior to final conversion to BigWig format using bedGraphToBigWig from UCSC. Transcripts were reconstructed using Cufflinks v2.1.1, with default settings (masking for rRNA, snRNA, Mt_rRNA, snoRNA and miRNA listed in Gencode v19 gene annotation). Output from Cufflinks was filtered to remove transcripts overlapping known gene structures. Putative eRNAs were identified from the remaining list using Bedtools intersect to select for mono-exonic transcripts overlapping T-bet super-enhancers.

***Quantification of RNA production at enhancers***

RNA-seq read coverage at each enhancer was calculated using featureCounts (http://bioinf.wehi.edu.au/featureCounts/ (Liao et al., 2014)). Read count was normalized to the total number of aligned reads per library and size of the T-bet binding site (kb). A 1 kb region up and downstream was appended to each enhancer coordinate to capture RNA-seq reads pertaining to the enhancer but extending beyond the T-bet boundary. Significance of differences in read number between T-bet sites in super-enhancers and enhancers or between different RNA samples was estimated using a K-S test.

Average RNA-seq read profiles around T-bet binding sites (identified in both replicates) that lied within super-enhancers or typical enhancers were calculated using ngsplot (https://code.google.com/p/ngsplot/ (Shen et al., 2014)). Enhancers overlapping multi-copy non-coding RNAs (tRNA, rRNA and snRNA, coordinates from RepeatMasker) as well as those within 2 kb of known protein-coding and non-coding genes (annotated by RefSeq and Gencode v19) were removed using intersectBed to ensure that previously annotated classes of RNAs were not being measured.

For comparison of FPKMs of individual eRNAs between Th1 and Th2 conditions, eRNA coordinates were defined in the Th1 RS total RNA library using Cufflinks. Reads corresponding to cufflink transcripts from comparison conditions were included if transcripts showed at least 1% overlap. Transcripts overlapping repeats (tRNA, rRNA and snRNA) or RefSeq annotated genes were removed from analysis. Putative eRNAs overlapping Ensembl genes but not annotated by RefSeq were assessed individually and manually removed if evidence of annotated gene transcription existed in our libraries. Multi-exonic transcripts were also excluded.

**Processing of published RNA-seq data**

Data from Wei and colleagues (GSM523209, GSM523211, GSM661236 and GSM661238 (Wei et al., 2011) was quality filtered and aligned as described above. Genes significantly overexpressed in Th1 cells compared to Th2 cells, and vice-versa, were identified using Cufflinks, with $p<0.05$ and log2 FC>2 thresholds. Previously identified sets of murine Th1 and Th2 genes were also taken from Additional file 1 of (Stubbington et al., 2015).

**Gene Ontology**

For functional analysis, sets of genes were uploaded to DAVID (http://david.abcc.ncifcrf.gov) and enriched Biological Process categories identified. Gene sets used were genes associated with T-bet super-enhancers and a proximal T-bet binding site and genes associated with typical T-bet enhancers and a proximal binding site (Figure S2E) and genes only occupied by P-TEFb in restimulated Th1 cells at proximal sites and genes occupied by P-TEFb at both proximal sites and intergenic sites (Figure 2F).

## Quantitative reverse-transcription PCR

Total RNA was purified from cells with Trizol, DNase-treated with DNA-free (Life Technologies) and the quality routinely verified using an Agilent bioanalyzer. RNA was reverse transcribed with SuperScriptIII (Life Technologies) primed with random primers. No-RT controls reactions were also performed for all eRNA qPCRs. The abundance of mRNAs and eRNAs was measured relative to Hprt by quantitative PCR using QuaniTect SYBR green (Qiagen) and a Applied Biosystems 7500 machine using the dCt method.

## qPCR primers used in this study

| *Target* | *Species* | *F primer* | *R primer* |
|---|---|---|---|
| Ifng | Mouse | GCCAAGTTTGAGGTGAGACG | GTGGACCACTCGGATGAGC |
| IFNG | Human | AAACGAGATGACTTCGAAAAG | ACAGTTCAGCCATCACTTGG |
| Med1 | Mouse | GCGAGCACCCTTCTCTTCTTG | GCCTCTCTGAGTCCTCGGTT |
| Med17 | Mouse | ACAGACATTGACTTGGATAAGAAGATAC | TGAATAGAAACCTTGATATACGCAGAC |
| Aff4 | Mouse | AGCAAAGCACATCTCACCAA | AATGCGTCATCTCTTTAAGTATTTC |
| Furin | Mouse | TTGGATGGCGAGGTGACTGATG | GCTGTAGATGTGGATGTGGTTGG |
| Dusp5 | Mouse | CTGAGTGCTGTGTGGATGTGAAG | CTGGTCATAGGCTGGTCTGTAGG |
| Xcl1 | Mouse | AGACTTCTCCTCCTGACTTTCCT | CTTCAGTCCCCACACCTTCCAC |
| Csf2 | Mouse | CGCTCACCCATCACTGTCACC | GACGACTTCTACCTCTTCATTCAACG |
| Ccl3 | Mouse | CCAAGTCTTCTCAGCGCCATAT | GCCGGTTTCTCTTAGTCAGGAAAATGA |
| Ccl4 | Mouse | GCTTTGTGATGGATTACTATGAGACC | CTCCTGAAGTGGCTCCTCCTG |
| Nest unspliced | Mouse | AATTGTGGTCGTTGTGTCTCC | GCCTGGGTTTCTGATACAGC |
| Nest spliced | Mouse | ATGCTAATTAAACAGAGTACCCGT | AACAAGAGTTACTGAAGCTGGA |
| Hprt | Mouse | TCAGTCAACGGGGGACATAAA | GGGGCTGTACTGCTTAACCAG |
| HPRT | Human | AGTCTGGCTTATATCCAACACTTG | GACTTTGCTTTCCTTGGTCAGG |
| Ifng eRNA -40kb | Mouse | AGCTCCCATTAATGACACACC | GTGGTAACACACACACACACC |
| Ifng eRNA -38.7kb | Mouse | AAAAGCCCAGAGTGTCAACC | GCTCTTCTCTTTCCAAGAAGC |
| Ifng eRNA -29kb | Mouse | CAAGGGTTGAGAATGGGTGC | TTGAAGATCACTCCTGCAAGT |
| Ifng eRNA -26kb | Mouse | TCCGTGTGACATGTCGTTTAG | AACAGAAGCCCTGCATTTTG |
| Ifng eRNA -10kb | Mouse | TTCTGCAGGCTCACTATTGG | GTGCGCTGCCTGTAAAGC |

**Experimental autoimmune uveitis and treatment with Flavopiridol and JQ1 *in vivo***

***EAU induction***

B10.RIII mice were housed at the Biological Service Unit, UCL Institute of Ophthalmology, in individually ventilated cabinets in specific pathogen-free conditions, according to UK Home Office Regulations. All animal studies were ethically reviewed and carried out in accordance with Animals (Scientific Procedures) Act 1986, Welfare and Treatment of Animals. B10.RIII mice aged 5-8 weeks (>20g body weight) were immunized subcutaneously in the flank with 300 μg $IRBP_{161–180}$ (SGIPYIISYLHPGNTILHVD, Cambridge Peptides) in PBS emulsified with Complete Freund's adjuvant (CFA) (Sigma) supplemented with 1.5 mg/ml *Mycobacterium tuberculosis* complete H37 Ra (Difco Microbiology) (1:1 v/v). Each mouse also received 0.4 μg *Bordetella pertussis* toxin (Sigma) intraperitoneally (Agarwal et al., 2012).

***Treatments***

Mice were treated with JQ1 (3, 30 mg/kg), Flavopiridol (3, 15 mg/kg) or vehicle control once daily by intraperitoneal injection for 5 consecutive days. JQ1 (kindly provided by James Bradner) and Flavopiridol (Sigma) were dissolved in DMSO and mixed with 10% hydroxypropyl-β-cyclodextrin (Sigma) in sterile water to improve solubility. Vehicle treated mice received equivalent volumes of DMSO mixed with 10% hydroxypropyl-β-cyclodextrin. A sample size of 7 mice per group was chosen based on a high reproducibility in EAU disease scores across the groups, and availability of mice within the age range from the colony used. Mice were only excluded from the study if, after 8 days post immunization, no signs of retinal inflammation were detectable by retinal imaging. This was less than 10% of the total number immunized for all experiments.

***Fundus imaging***

On day 8-9 post immunization, *in vivo* imaging of retinal fundus was performed (Micron III retinal imaging microscope, Phoenix Research Labs) to screen for early signs of retinal inflammation (grade 1). Only those showing positive signs were selected for use. The day before termination (day 14-15), retinal disease progression was monitored and scored by retinal fundoscopy, using an established scoring system (Agarwal et al., 2012).

### *Ocular dissection*

Enucleated eyes were fixed in 4% glutaraldehyde for 2 hours, followed by overnight fixation in formalin. Eyes were then processed, orientated and embedded for paraffin wax sectioning and cut in 4-5 μm sections, stained with eosin and counterstained with hematoxylin. The sections were scored using an established grading system (Agarwal et al., 2012). The significance of the difference between drug treated and untreated mice was measured using an unpaired t-test with Welch's correction, two-tailed (Gardner et al., 2013). Cell counting was performed on rehydrated paraffin-embedded sections (3-4 μm), using rat anti-mouse CD45 monoclonal antibody (1:400; Serotec) or rabbit anti-mouse CD3 antibody (1:400; Santa Cruz), followed by DAB (Vector Lab, Peterborough, UK), and counterstained with hematoxylin. Positive cells were counted in a minimum of three separate retinal fields, and data shown as means ± SD (n>4 mice per group).

For retinal cells, enucleated eyes were dissected in 100 μl of cold DMEM media. Following incision at the limbus with a 29G needle, a circumferential cut was made. Iris was dissected away releasing anterior chamber infiltrating cells into the dissection media. The retina was then removed from the eye cup leaving the Sclera/RPE/choroid intact. The dissection media and retina were then pipetted up into a 1.5ml tube and mechanically disrupted by vortexing to obtain a single cell suspension followed by centrifugation through a single well of a 96 well 60 μm cell strainer plate (Millipore). The resulting cell pellets were re-suspended and stained for immunophenotyping.

### *Peripheral lymph node dissection*

The inguinal lymph nodes were dissected asceptically and cell suspensions were prepared as previously described (Gardner et al., 2013).

### *Flow cytometry*

Single cell suspension were prepared from the retinas and inguinal lymph nodes and cells were stimulated with PMA (50 ng/ml) and ionomycin (1 μM) in the presence of 3 μM monensin for 4 hours. Surface markers were stained in PBS with 1% BSA for 20 min on ice, then the cells were fixed with 1% formaldehyde, permeabilized with 0.1% saponin in PBS and stained with fluorophore-conjugated antibodies in permeabilization buffer. For the detection of Ctla4, the cells

were first stained on the cell surface and, after permeabilization, the staining was repeated intracellularly. Fluorophore-conjugated antibodies used were: AlexaFluor700 anti-CD4 (RM4-5, BD Biosciences), phycoerythrin-cyanine 7 anti-CD3 (145-2C11, eBiosciences), eFluor 450 anti-IFN-γ (XMG1.2, eBiosciences), Fluorescein isothiocyanate anti-TNFα (MP6-XT22, eBiosciences), phycoerythrin anti-FasL (MFL3, eBiosciences), phycoerythrin anti-Ctla4 (UC10-4F10-11), Fluorescein isothiocyanate anti-IL18R1 (112614, R&D), Fluorescein isothiocyanate anti-ICAM1 (YN1/1.7.4, eBiosciences) and Peridinin-chlorophyll-eFlour 710 anti-CD29 (HMb1-1, eBiosciences). Data were collected using an LSRFortessa (BD Biosciences) then analyzed with Flow Jo software (Treestar).

**shRNA knock-downs**

To express shRNAs in mouse T cells, a miR-30 based shRNA expression element was fused to the 3' end of the reporter genes Thy1.1 and TurboRFP (tRFP). These reporter-shRNA cassettes were subsequently inserted into the pMY retroviral expression vector to create pMY-Thy1.1-miR30 and pMY-tRFP-miR30. The shRNA sequences targeting Med1 and Med17 have previously been described (Van Essen et al., 2009; Kagey et al., 2010). shRNAs targeting the firefly luciferase and cre recombinase genes were used as a negative controls. All shRNA sequences used are listed below.

shMed1: CGCAAGCACAAATTCTTCTAA

shMed17: AGAGATGGTCGGGTAATCA

shAff4: GCAACATTCAAGTCAGTCTTT

shFLuc: GGTGGCTCCCGCTGAATTGGA

shCre: GTGGGAGAATGTTAATCCATA

Retroviral particle preparation and infection were performed as previously described (Kanhere et al. 2012). Thy1.1+ cells were isolated on day 4 of Th1 polarization using CD90.1 magnetic beads (Miltenyi). For Med1/Med17 double knock-down, the cells were transduced with a mixture of pMY-Thy1.1-shMed1 and pMY-tRFP-Med17 viral particles and Thy1.1+tRFP+ double positive cells were purified by flow cell sorting on day 6. On day 7 the cells were either restimulated with 2 μg/ml anti-CD3/CD28 or left unstimulated for 6 hrs. Med1, Med17 and Aff4 knockdown were

confirmed by qRT-PCR. Med1 knockdown was additionally confirmed by western blotting (Figure S4I). No suitable antibodies for western blotting were available for Med17 and Aff4.

**Co-immunoprecipitation and immunoblot analysis**

HEK293 cells were transiently transfected with FLAG-T-bet and HA-CyclinT1 or HA-Cdk9 expression vectors using polyethyleneimine. Cells were lysed in RIPA buffer (50 mM Tris, pH 8.0, 150 mM NaCl, 1 mM EDTA, 1% IGEPAL CA-630, 0.5% sodium-deoxycholate, 10% glycerol and Complete protease inhibitors (Roche)) 48 hrs after transfection. For co-immunoprecipitation of FLAG-T-bet with endogenous Cyclin T1 and Cdk9, EL4-FLAG-T-bet cells were stimulated for 5 hrs with PMA and ionomycin, followed by lysis in RIPA buffer described above. Whole-cell lysates were immunoprecipitated at 4 °C with anti-FLAG (M2; Sigma), anti-Cyclin T1 (H-245, Santa Cruz), anti-Cdk9 (C-20, Santa Cruz), mouse polyclonal IgG (sc-2025, Santa Cruz) or rabbit polyclonal IgG (P120-101; Bethyl) coupled to magnetic protein-G beads (Dynal). Immune complexes were washed once with wash buffer (40 mM Tris, pH 7.5, 150 mM NaCl, 0.3% IGEPAL CA-630 and 10% glycerol), five times with wash buffer containing 500 mM NaCl and once with HD buffer (20 mM Tris, pH 8.0, 150 mM NaCl and 10% glycerol), reuspended in Laemmli buffer and incubated at 95°C for 5 min. Proteins were resolved on a 8% Tris-glycine gel and transferred to a nitrocellulose membrane. Antibodies for immunoblotting were identical to those used for immunoprecipitation, in addition to anti-Suz12 (P15, Santa Cruz).

## SUPPLEMENTAL REFERENCES


Cousins, D.J., Lee, T.H., and Staynov, D.Z. (2002). Cytokine coexpression during human Th1/Th2 cell differentiation: direct evidence for coordinated expression of Th2 cytokines. Journal of immunology *169*, 2498-2506.

He, N., Chan, C.K., Sobhian, B., Chou, S., Xue, Y., Liu, M., Alber, T., Benkirane, M., and Zhou, Q. (2011). Human Polymerase-Associated Factor complex (PAFc) connects the Super Elongation Complex (SEC) to RNA polymerase II on chromatin. Proc Natl Acad Sci U S A *108*, E636-645.

Jenner, R.G., Townsend, M.J., Jackson, I., Sun, K., Bouwman, R.D., Young, R.A., Glimcher, L.H., and Lord, G.M. (2009). The transcription factors T-bet and GATA-3 control alternative pathways of T-cell differentiation through a shared set of target genes. Proc Natl Acad Sci U S A *106*, 17876-17881.

Kagey, M. H., Newman, J. J., Bilodeau, S., Zhan, Y., Orlando, D. A., van Berkum, N. L., Ebmeier, C. C., Goossens, J., Rahl, P. B., Levine, S. S., Taatjes, D. J., Dekker, J., Young, R.A. (2010). Mediator and cohesin connect gene expression and chromatin architecture. Nature *467,* 430-435.

Landt, S.G., Marinov, G.K., Kundaje, A., Kheradpour, P., Pauli, F., Batzoglou, S., Bernstein, B.E., Bickel, P., Brown, J.B., Cayting, P., *et al.* (2012). ChIP-seq guidelines and practices of the ENCODE and modENCODE consortia. Genome Res *22*, 1813-1831.

Li, Q., Brown, J.B., Huang, H., Bickel, P.J. (2011). Measuring reproducibility of high-throughput experiments. Ann Appl Stat *5*, 1752-1779.

Liao, Y., Smyth, G.K., and Shi, W. (2014). featureCounts: an efficient general purpose program for assigning sequence reads to genomic features. Bioinformatics *30,* 923-930.

Lin, C., Garrett, A.S., De Kumar, B., Smith, E.R., Gogol, M., Seidel, C., Krumlauf, R., and Shilatifard, A. (2011). Dynamic transcriptional events in embryonic stem cells mediated by the super elongation complex (SEC). Genes Dev *25*, 1486-1498.

Lin, C., Garruss, A.S., Luo, Z., Guo, F., and Shilatifard, A. (2013). The RNA Pol II elongation factor Ell3 marks enhancers in ES cells and primes future gene activation. Cell *152*, 144-156.

Lin, C., Smith, E.R., Takahashi, H., Lai, K.C., Martin-Brown, S., Florens, L., Washburn, M.P., Conaway, J.W., Conaway, R.C., and Shilatifard, A. (2010). AFF4, a component of the ELL/P-TEFb elongation complex and a shared subunit of MLL chimeras, can link transcription elongation to leukemia. Mol Cell *37*, 429-437.

Luo, Z., Lin, C., Guest, E., Garrett, A.S., Mohaghegh, N., Swanson, S., Marshall, S., Florens, L., Washburn, M.P., and Shilatifard, A. (2012). The super elongation complex family of RNA polymerase II elongation factors: gene target specificity and transcriptional output. Mol Cell Biol *32*, 2608-2617.

Mueller, D., Garcia-Cuellar, M.P., Bach, C., Buhl, S., Maethner, E., and Slany, R.K. (2009). Misguided transcriptional elongation causes mixed lineage leukemia. PLoS Biol *7*, e1000249.

Rahl, P.B., Lin, C.Y., Seila, A.C., Flynn, R.A., McCuine, S., Burge, C.B., Sharp, P.A., and Young, R.A. (2010). c-Myc regulates transcriptional pause release. Cell *141*, 432-445.

Shao, Z., Zhang, Y., Yuan, G. C., Orkin, S. H., Waxman, D. J. (2012). MAnorm: a robust model for quantitative comparison of ChIP-Seq data sets. Genome Biol. *3*, R16.

Shen, L., Shao, N., Liu, X., and Nestler, E. (2014). ngs.plot: Quick mining and visualization of next-generation sequencing data by integrating genomic databases. BMC Genomics *15*, 284.

Su, A.I., Wiltshire, T., Batalov, S., Lapp, H., Ching, K.A., Block, D., Zhang, J., Soden, R., Hayakawa, M., Kreiman, G., *et al.* (2004). A gene atlas of the mouse and human protein-encoding transcriptomes. Proc Natl Acad Sci U S A *101*, 6062-6067.

Zeitlinger J., Stark A., Kellis M, Hong JW, Nechaev S, Adelman K, Levine M, Young RA. (2007). RNA polymerase stalling at developmental control genes in the Drosophila melanogaster embryo. Nat Genet *39*, 1512-1516.

Zhang, Y., Liu, T., Meyer, C.A., Eeckhoute, J., Johnson, D.S., Bernstein, B.E., Nusbaum, C., Myers, R.M., Brown, M., Li, W., and Liu, X.S. (2008). Model-based analysis of ChIP-Seq (MACS). Genome Biol *9*, R137.
